# Performance of a Prostate-Specific Membrane Antigen Positron Emission Tomography/Computed Tomography–Derived Risk-Stratification Tool for High-risk and Very High-risk Prostate Cancer

**DOI:** 10.1001/jamanetworkopen.2021.38550

**Published:** 2021-12-13

**Authors:** Michael Xiang, Ting Martin Ma, Ricky Savjani, Erqi L. Pollom, R. Jeffrey Karnes, Tristan Grogan, Jessica K. Wong, Giovanni Motterle, Jeffrey J. Tosoian, Bruce J. Trock, Eric A. Klein, Bradley J. Stish, Robert T. Dess, Daniel E. Spratt, Avinash Pilar, Chandana Reddy, Rebecca Levin-Epstein, Trude B. Wedde, Wolfgang A. Lilleby, Ryan Fiano, Gregory S. Merrick, Richard G. Stock, D. Jeffrey Demanes, Brian J. Moran, Hartwig Huland, Phuoc T. Tran, Santiago Martin, Rafael Martinez-Monge, Daniel J. Krauss, Eyad I. Abu-Isa, Ridwan Alam, Zeyad Schwen, Thomas M. Pisansky, C. Richard Choo, Daniel Y. Song, Stephen Greco, Curtiland Deville, Todd McNutt, Theodore L. DeWeese, Ashley E. Ross, Jay P. Ciezki, Paul C. Boutros, Nicholas G. Nickols, Prashant Bhat, David Shabsovich, Jesus E. Juarez, Natalie Chong, Patrick A. Kupelian, Matthew B. Rettig, Nicholas G. Zaorsky, Alejandro Berlin, Jonathan D. Tward, Brian J. Davis, Robert E. Reiter, Michael L. Steinberg, David Elashoff, Eric M. Horwitz, Rahul D. Tendulkar, Derya Tilki, Johannes Czernin, Andrei Gafita, Tahmineh Romero, Jeremie Calais, Amar U. Kishan

**Affiliations:** 1 Department of Radiation Oncology, University of California, Los Angeles; 2 Department of Radiation Oncology, Stanford University, Stanford, California; 3 Department of Urology, Mayo Clinic, Rochester, Minnesota; 4 Department of Medicine Statistics Core, David Geffen School of Medicine at UCLA, Los Angeles, California; 5 Department of Radiation Oncology, Fox Chase Cancer Center, Philadelphia, Pennsylvania; 6 Department of Urology, University of Michigan, Ann Arbor; 7 Department of Urology, Brady Urological Institute, Johns Hopkins University, Baltimore, Maryland; 8 Department of Urology, Glickman Urological and Kidney Institute, Cleveland Clinic, Cleveland, Ohio; 9 Department of Radiation Oncology, Mayo Clinic, Rochester, Minnesota; 10 Department of Radiation Oncology, University of Michigan, Ann Arbor; 11 Department of Radiation Oncology, University of Toronto, Toronto, Ontario, Canada; 12 Department of Radiation Oncology, Taussig Cancer Institute, Cleveland Clinic, Cleveland, Ohio; 13 Department of Oncology, Oslo University Hospital, Norwegian Radium Hospital, Oslo, Norway; 14 Schiffler Cancer Center, Wheeling Hospital, Wheeling Jesuit University, Wheeling, West Virginia; 15 Department of Radiation Oncology, Icahn School of Medicine at Mount Sinai, New York City, New York; 16 Prostate Cancer Foundation of Chicago, Westmont, Illinois; 17 Martini-Klinik Prostate Cancer Center, University Hospital Hamburg Eppendorf, Hamburg, Germany; 18 Department of Radiation Oncology and Molecular Radiation Sciences, Johns Hopkins University School of Medicine, Baltimore, Maryland; 19 Department of Oncology, Clínica Universitaria de Navarra, University of Navarra, Pamplona, Spain; 20 Oakland University William Beaumont School of Medicine, Royal Oak, Michigan; 21 Department of Urology, Northwestern University Feinberg School of Medicine, Chicago, Illinois; 22 Department of Human Genetics, University of California, Los Angeles; 23 Department of Radiation Oncology, Veterans Affairs (VA) Greater Los Angeles Healthcare System, Los Angeles, California; 24 Division of Hematology and Oncology, Department of Medicine, University of California, Los Angeles; 25 Department of Hematology and Oncology, Veterans Affairs (VA) Greater Los Angeles Healthcare System, Los Angeles, California; 26 Department of Radiation Oncology, Penn State Cancer Institute, Hershey, Pennsylvania; 27 Department of Radiation Oncology, Huntsman Cancer Institute, University of Utah, Salt Lake City; 28 Department of Urology, University of California, Los Angeles; 29 Department of Urology, University Hospital Hamburg-Eppendorf, Hamburg, Germany; 30 Ahmanson Translational Theranostics Division, Department of Molecular and Medical Pharmacology, UCLA Medical Center, Los Angeles, California

## Abstract

**Importance:**

Prostate-specific membrane antigen (PSMA) positron emission tomography/computed tomography (PET/CT) can detect low-volume, nonlocalized (ie, regional or metastatic) prostate cancer that was occult on conventional imaging. However, the long-term clinical implications of PSMA PET/CT upstaging remain unclear.

**Objectives:**

To evaluate the prognostic significance of a nomogram that models an individual’s risk of nonlocalized upstaging on PSMA PET/CT and to compare its performance with existing risk-stratification tools.

**Design, Setting, and Participants:**

This cohort study included patients diagnosed with high-risk or very high-risk prostate cancer (ie, prostate-specific antigen [PSA] level >20 ng/mL, Gleason score 8-10, and/or clinical stage T3-T4, without evidence of nodal or metastatic disease by conventional workup) from April 1995 to August 2018. This multinational study was conducted at 15 centers. Data were analyzed from December 2020 to March 2021.

**Exposures:**

Curative-intent radical prostatectomy (RP), external beam radiotherapy (EBRT), or EBRT plus brachytherapy (BT), with or without androgen deprivation therapy.

**Main Outcomes and Measures:**

PSMA upstage probability was calculated from a nomogram using the biopsy Gleason score, percentage positive systematic biopsy cores, clinical T category, and PSA level. Biochemical recurrence (BCR), distant metastasis (DM), prostate cancer–specific mortality (PCSM), and overall survival (OS) were analyzed using Fine-Gray and Cox regressions. Model performance was quantified with the concordance (C) index.

**Results:**

Of 5275 patients, the median (IQR) age was 66 (60-72) years; 2883 (55%) were treated with RP, 1669 (32%) with EBRT, and 723 (14%) with EBRT plus BT; median (IQR) PSA level was 10.5 (5.9-23.2) ng/mL; 3987 (76%) had Gleason grade 8 to 10 disease; and 750 (14%) had stage T3 to T4 disease. Median (IQR) follow-up was 5.1 (3.1-7.9) years; 1221 (23%) were followed up for at least 8 years. Overall, 1895 (36%) had BCR, 851 (16%) developed DM, and 242 (5%) died of prostate cancer. PSMA upstage probability was significantly prognostic of all clinical end points, with 8-year C indices of 0.63 (95% CI, 0.61-0.65) for BCR, 0.69 (95% CI, 0.66-0.71) for DM, 0.71 (95% CI, 0.67-0.75) for PCSM, and 0.60 (95% CI, 0.57-0.62) for PCSM (*P* < .001). The PSMA nomogram outperformed existing risk-stratification tools, except for similar performance to Staging Collaboration for Cancer of the Prostate (STAR-CAP) for PCSM (eg, DM: PSMA, 0.69 [95% CI, 0.66-0.71] vs STAR-CAP, 0.65 [95% CI, 0.62-0.68]; *P* < .001; Memorial Sloan Kettering Cancer Center nomogram, 0.57 [95% CI, 0.54-0.60]; *P* < .001; Cancer of the Prostate Risk Assessment groups, 0.53 [95% CI, 0.51-0.56]; *P* < .001). Results were validated in secondary cohorts from the Surveillance, Epidemiology, and End Results database and the National Cancer Database.

**Conclusions and Relevance:**

These findings suggest that PSMA upstage probability is associated with long-term, clinically meaningful end points. Furthermore, PSMA upstaging had superior risk discrimination compared with existing tools. Formerly occult, PSMA PET/CT–detectable nonlocalized disease may be the main driver of outcomes in high-risk patients.

## Key Points

**Question:**

Is previously occult, nonlocalized (regional or metastatic) disease detected on prostate-specific membrane antigen (PSMA) positron emission tomography/computed tomography (PET/CT) associated with clinically significant outcomes in patients with high-risk and very high-risk prostate cancer?

**Findings:**

In this cohort study of 5275 patients, a nomogram estimative of an individual’s risk of nonlocalized disease on PSMA PET/CT was significantly associated with long-term outcomes, including distant metastasis and prostate cancer–specific mortality. The nomogram performed favorably compared with other risk-stratification tools.

**Meaning:**

These findings suggest that previously occult disease may be associated with outcomes in this patient population.

## Teaser

This cohort study evaluates the prognostic significance of a nomogram that models an individual’s risk of nonlocalized upstaging on prostate-specific membrane antigen (PSMA) positron emission tomography/computed tomography (PET/CT) and compares its performance with existing risk-stratification tools.

## Introduction

High- and very high-risk prostate cancer has a heterogeneous disease course, perhaps due to a wide spectrum of intrinsic aggressiveness and an inability to evaluate the true extent of disease at initial diagnosis.^1,2,3,4^ Metastatic failures occur more commonly than local failures, suggesting that a substantial number of patients harbor occult spread at presentation.^5^ Prostate-specific membrane antigen (PSMA) positron emission tomography/computed tomography (PET/CT) has superior sensitivity and specificity compared with conventional imaging, potentially dramatically altering initial stage, management, and outcomes.^6,7^ However, given that nonlocalized (nodal or metastatic) disease detected only by PSMA PET/CT is generally low volume, it is unclear whether lesions detected only on PSMA PET/CT are associated with disease lethality and other long-term, clinically meaningful end points.

Recently, we developed a PSMA nomogram to calculate the probability of finding nonlocalized disease on PSMA PET/CT in patients who appear to have cN0M0 disease on conventional imaging.^8,9^ In this study, we evaluated the significance of the PSMA nomogram (and, by proxy, PSMA PET/CT itself) on long-term, clinically meaningful end points (including distant metastasis [DM], prostate cancer–specific mortality [PCSM], and overall survival [OS]) using internal validation, external validation, and comparison with existing risk-stratification models. We hypothesized that the PSMA nomogram would provide more accurate prognostication than existing risk-stratification tools in high-risk patients based on the underlying hypothesis that previously occult, PSMA PET/CT–detectable nonlocalized disease may be the primary reason for treatment failures and underlie the heterogeneity in clinical outcomes.

## Methods

### Data Sources, Patient Selection, and Variables

The multi-institutional cohort consisted of patients treated at 15 tertiary referral centers between April 1995 and August 2018 with high- and very high-risk prostate cancer (per National Comprehensive Cancer Network criteria), defined as prostate-specific antigen (PSA) level greater than 20 ng/mL (to convert to micrograms per liter, multiply by 1.0), Gleason score 8 to 10, or clinical stage T3 to T4.^10^ Inclusion criteria were as follows: absence of nodal or metastatic disease on conventional imaging and curative-intent treatment with radical prostatectomy (RP), external beam radiation therapy (EBRT), or EBRT plus brachytherapy (BT), with or without androgen deprivation therapy (ADT). Institutional review board approval was obtained for all contributing centers, with the requirement for informed consent waived due to the retrospective nature of the study. The Strengthening the Reporting of Observational Studies in Epidemiology (STROBE) reporting guideline for cohort studies and the Transparent Reporting of a Multivariable Prediction Model for Individual Prognosis or Diagnosis (TRIPOD) reporting guideline for prediction model development/validation were followed.

The Surveillance, Epidemiology, and End Results (SEER) database is a population-based cancer registry covering approximately 28% of the US population.^11^ Patients diagnosed with high- and very high-risk prostate adenocarcinoma were selected from January 2010 (when biopsy percent positive cores became available) to December 2016 (the latest year with treatment data at the time this study was conducted). In SEER, patients who undergo surgery have their pathological N category supersede the clinical N category, which precludes identification of patients with clinical node-negative disease; thus, treatment with RP was excluded.

The National Cancer Database (NCDB) is a hospital-based cancer registry representing more than 70% of cancer diagnoses in the United States.^12^ While there is moderate overlap in the patients found in NCDB and SEER, each database has patients not found in the other.^13^ Patients diagnosed with high- and very high-risk prostate adenocarcinoma were selected from January 2010 to December 2016 as was done in SEER. In NCDB (unlike in SEER), clinical N category is preserved in patients who undergo surgery, and patients who underwent RP were included.

Given the aim of prognostication based solely on information available at initial diagnosis, patients in SEER and NCDB were included regardless of subsequent treatment (except for patients who underwent RP in SEER, as described earlier). Age at diagnosis, PSA level, clinical T stage, Gleason score, number of positive and total number of biopsy scores (to calculate percentage of positive cores), and treatment type were extracted directly from the registries. PCSM is available in SEER, and overall survival (OS) is available in SEER and NCDB; biochemical recurrence (BCR) and DM are not available. Patients missing any data required to calculate their PSMA nomogram score or missing vital status, cause of death (in SEER), or follow-up duration were excluded. For comparison, patients with known (ie, clinically overt) node-positive (N1) or metastatic (M1) disease at diagnosis were also identified from both databases. Analyses involving SEER and NCDB were deemed exempt by the University of California, Los Angeles, institutional review board.

### Nomograms and Other Risk-Stratification Tools

PSMA nomograms were derived from 262 men with cN0M0 (by conventional imaging) high- or very high-risk prostate cancer who underwent PSMA PET/CT during 2 prospective clinical trials, as previously described.^8^ Briefly, a nomogram to estimate the probability of any nonlocalized PSMA-based upstaging was built using logistic regression on 4 core variables: initial PSA level, biopsy Gleason grade group, percentage positive cores (based on systematic cores), and clinical T category. The accuracy of this nomogram for correctly identifying nonlocalized disease on PSMA PET/CT was area under the curve (AUC) 0.75 (95% CI, 0.67-0.83).^8^ The nomogram is available as an online calculator.^9^

Comparison models were chosen as previously validated tools used for risk-stratification and prognostication.^14^ These were the Cancer of the Prostate Risk Assessment (CAPRA) groups,^15^ the Staging Collaboration for Cancer of the Prostate (STAR-CAP) stage groups,^16^ and the Memorial Sloan Kettering Cancer Center (MSKCC) preprostatectomy nomogram for 5-year risk of disease progression.^17^

### Statistical Analysis

In the multi-institutional cohort, BCR was defined as initiation of salvage ADT, PSA level greater than 0.2 ng/mL in patients treated with RP, or PSA greater than the lowest value plus 2 ng/mL in patients treated with EBRT and EBRT plus BT. DM was defined as a clinical diagnosis of metastatic disease (eg, imaging); pathological confirmation of DM was not required. PCSM was defined as either clinical documentation or inclusion of prostate cancer as a primary cause of death on the death certificate. In the SEER database, PCSM was defined as cause of death coded to prostate cancer. In SEER and NCDB, vital status and length of OS were extracted directly from the database for each case.

Model performance was evaluated using time-dependent receiver operating characteristic (ROC) curves and concordance (C) indices, allowing for censoring and competing risks. Confidence intervals were computed using the independent, identically distributed representation of the time-dependent AUC estimators. For the NCDB cohort, because of the much larger cohort size, confidence intervals could not be obtained analytically; thus, variances were estimated using 10 000 bootstrapped samples. Models were also evaluated using decision curve analysis,^18^ indices of predictive accuracy (IPAs),^16^ and calibration plots.^8^

Associations between nomogram risk and clinical end points were modeled with nomogram risk as a continuous variable, with age as a covariate to control for the risk of competing events or death, using Cox and Fine-Gray regression to obtain age-adjusted hazard ratios and subdistribution hazard ratios, respectively. In the Fine-Gray regressions, death in the absence of the end point of interest was treated as a competing risk. Additional subgroup analyses were performed, stratified by treatment type.

PSMA nomogram cut points were selected by a stepwise method using the end point of 8-year DM in the multi-institutional cohort. Details of this method are provided in the eMethods in the Supplement. The cut points were then used to partition patients into 4 nomogram-defined risk groups. Time-to-event outcomes were compared using Gray test or the log-rank test.

Subgroup and sensitivity analyses were performed stratified by type of treatment. Calculations were performed using MATLAB version R2020a (MathWorks, Inc); R version 4.0.3 (R Project for Statistical Computing); R packages cmprsk (version 2.2-10), timeROC (version 0.4), and riskRegression (version 2020.12.08), R function *stdca*,^19^ Python version 3.8.2 (Python Software Foundation), and Python module scikit-survival (version 0.14.0). All tests were 2-sided and considered significant at *P* < .05.

## Results

**Figure 1.**
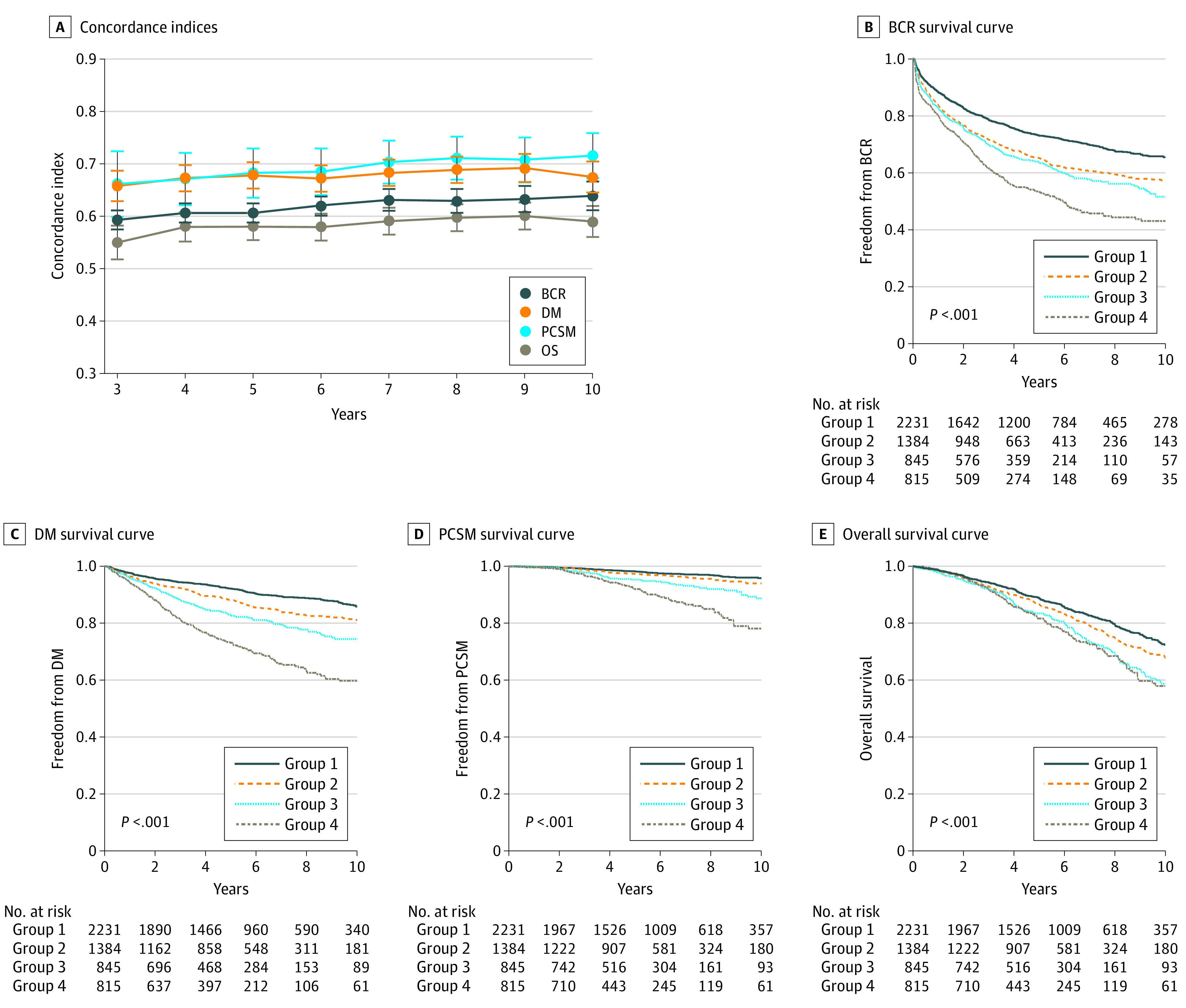
Prognostic Significance of the Prostate-Specific Membrane Antigen Nomogram in the Multi-institutional Cohort A, Concordance indices of the nomogram for biochemical recurrence (BCR), distant metastasis (DM), prostate cancer-specific mortality (PCSM), and overall survival (OS) at 3 to 10 years. Error bars are 95% CIs. B-E, Survival curves (freedom from BCR, freedom from DM, freedom from PCSM, and OS) according to nomogram-defined risk groups, corresponding to upstaging risks of 14% or less (group 1), 14.1% to 27% (group 2), 27.1% to 41% (group 3), and greater than 41% (group 4).

In the multi-institutional cohort of 5275 patients (Table), the median (IQR) age was 66 (60-72); 2883 (55%) received RP, 1669 (32%) received EBRT, and 723 (14%) received EBRT plus BT; the median PSA level was 10.5 (5.9-23.2) ng/mL; 3987 (76%) had Gleason grade 8 to 10 disease; 750 (14%) had T3 or T4 disease; and median (IQR) follow-up was 5.1 (3.1-7.9) years. A total of 1221 (23%) were followed up for at least 8 years. The selection procedure used to identify patients in the multi-institutional cohort appears in eTable 1 in the Supplement. Overall, 1895 (36%) experienced BCR, 851 (16%) developed DM, and 242 (5%) died of prostate cancer. Based on the C indices, the PSMA nomogram was significantly prognostic of all clinical end points, with 8-year C-indices of 0.63 (95% CI, 0.61-0.65) for BCR, 0.69 (95% CI, 0.66-0.71) for DM, 0.71 (95% CI, 0.67-0.75) for PCSM, and 0.60 (95% CI, 0.57-0.62) for OS (*P* < .001) (Figure 1A). Similar results were obtained in subgroup analyses stratified by treatment type, except performance was lower for patients treated with EBRT plus BT (eFigure 1 in the Supplement). Model informativeness was also evaluated using decision curve analysis (eFigure 2 in the Supplement).

**Table.** Baseline Patient Characteristics of the Multi-institutional High-risk Cohort, the NCDB High-risk Cohort, and the SEER High-risk Cohort

Characteristic	Patients, No. (%)		
	Multi-institutional cohort (n = 5275)	SEER cohort (n = 23 989)	NCDB cohort (n = 88 909)
Follow-up, median (IQR), y	5.1 (3.1-7.9)	2.7 (1.2-4.5)	3.8 (2.5-5.7)
Age, median (IQR)	66 (60-72)	71 (65-77)	67 (61-73)
Gleason grade group			
1	402 (8)	1528 (6)	6387 (7)
2	460 (9)	2253 (9)	9389 (11)
3	426 (8)	2125 (9)	7058 (8)
4	2512 (48)	10 298 (43)	38 348 (43)
5	1475 (28)	7785 (32)	27 727 (31)
Clinical T category			
T1	2582 (49)	12922 (54)	52907 (60)
T2	1943 (37)	8395 (35)	24871 (28)
T3	684 (13)	2401 (10)	10543 (12)
T4	66 (1)	271 (1)	588 (1)
PSA, median (IQR), ng/mL	10.5 (5.9-23.2)	13.8 (7.4-27.4)	10.9 (6.2-25.9)
Positive cores, median (IQR), %	50 (33-83)	50 (33-83)	50 (30-80)
Primary therapy			
Surgery	2883 (55)	0 (0)	37017 (42)
EBRT	1669 (32)	14087 (59)	34529 (39)
EBRT plus BT	723 (14)	2065 (9)	3744 (4)
Unknown or other	0 (0)	7837 (33)	13619 (15)
PSMA upstage risk, median (IQR)			
Any non-localized (N+M)	16.7 (8.5-31.8)	18.2 (9.3-35.4)	17.3 (8.5-32.1)
Regional nodal (N)	15.0 (8.0-27.3)	16.2 (8.6-30.6)	15.4 (8.0-27.7)
Distant metastatic (M)	5.1 (2.1-12.2)	5.4 (2.1-13.0)	4.7 (1.9-11.7)
PSMA nomogram risk group			
Group 1, ≤14% upstage risk	2231 (42)	9285 (39)	36 694 (41)
Group 2, 14.1%-27% upstage risk	1384 (26)	6087 (25)	23 418 (26)
Group 3, 27.1%-41% upstage risk	845 (16)	3922 (16)	14 153 (16)
Group 4, >41% upstage risk)	815 (16)	4695 (20)	14 644 (16)

Abbreviations: BT, brachytherapy; EBRT, external beam radiotherapy; NCDB, National Cancer Database; PSA, prostate-specific antigen; PSMA, prostate-specific membrane antigen; SEER, Surveillance, Epidemiology, and End Results.

SI conversion: To convert PSA to micrograms per liter, multiply by 1.0.

In age-adjusted regression analyses, PSMA nomogram risk was significantly prognostic for all clinical end points (eFigure 3 in the Supplement). Results remained consistent in subgroup analyses stratified by treatment type, except the nomogram was not significantly prognostic for EBRT plus BT for PCSM and OS. Furthermore, nomogram risk was discretized into 4 groups (as described in the Methods), corresponding to PSMA PET/CT upstaging risks of 14% or less (2231 patients [42%]), 14.1% to 27% (1384 patients [26%]), 27.1% to 41% (845 patients [16%]), and greater than 41% (815 [16%]). A 10-fold cross-validation repeated 100 times and a 1000-repeat bootstrap validation both yielded similar cut points (eTable 2 and eTable 3 in the Supplement). The 4 nomogram-defined risk groups were also significantly prognostic in time-to-event analyses (*P* < .001) (Figure 1B-E).

**Figure 2.**
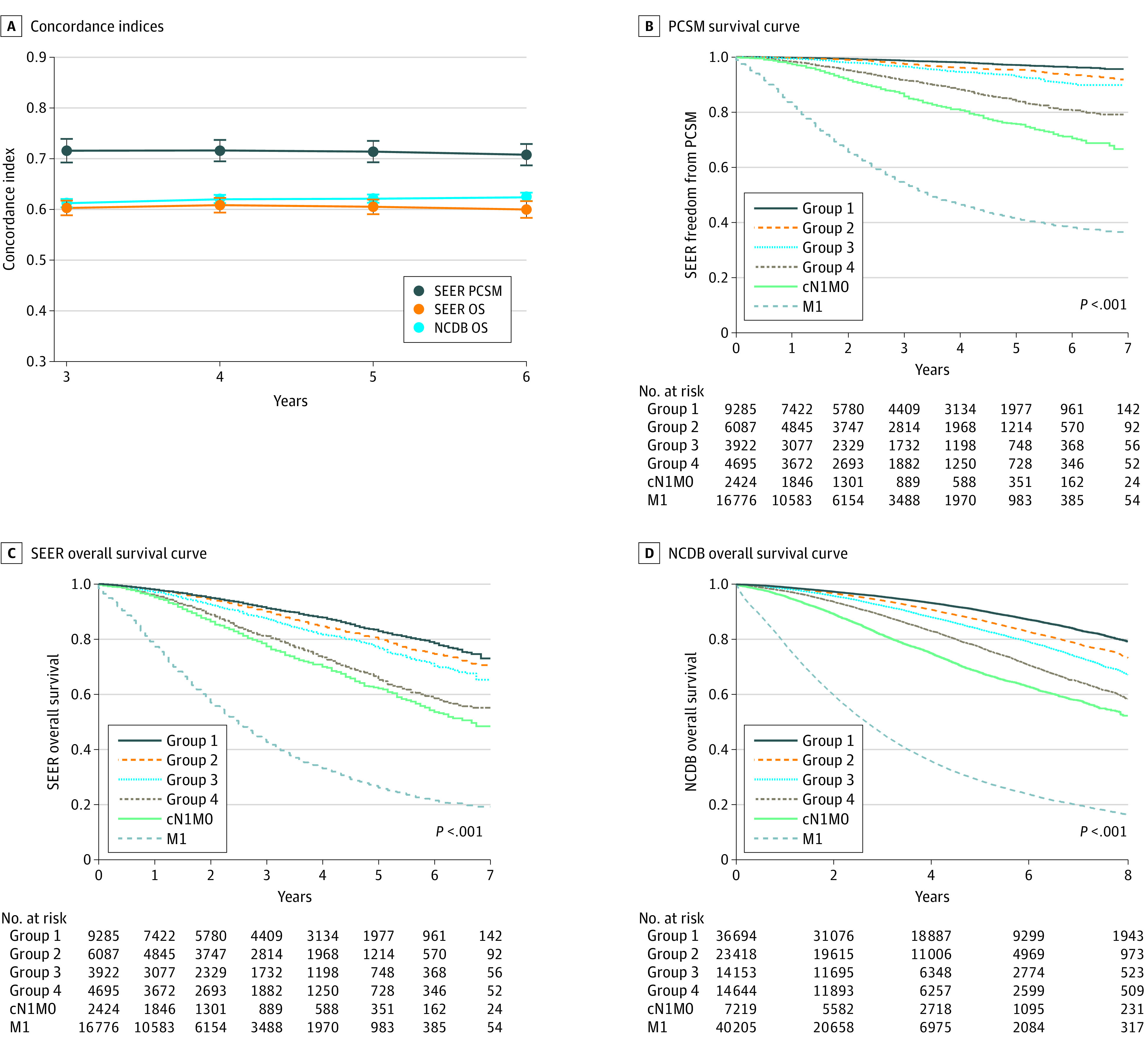
Prognostic Significance of the Prostate-Specific Membrane Antigen Nomogram in the Registry-Based Cohorts A, Concordance indices of the nomogram for prostate cancer-specific mortality (PCSM) in Surveillance, Epidemiology, and End Results (SEER) and overall survival (OS) in SEER and the National Cancer Database (NCDB) at 3 to 6 years. Error bars are 95% CIs. B-D, Survival curves (freedom from PCSM in SEER, OS in SEER, and OS in NCDB) according to nomogram-defined risk groups, corresponding to upstaging risks of 14% or less (group 1), 14.1% to 27% (group 2), 27.1% to 41% (group 3), and greater than 41% (group 4). *P* values are for groups 1-4 only, while outcomes of patients with cN1M0 (group 5) and M1 (group 6) disease are graphed for comparison.

To externally validate these findings, we used 2 large registry-based cohorts: the SEER database involving 23 989 patients, and the NCDB involving 88 909 patients (Table). The selection procedures used to identify patients for these cohorts are included in eTable 4 and eTable 5 in the Supplement). The 5-year C indices were 0.71 (95% CI, 0.69-0.74) for PCSM in SEER, 0.61 (95% CI, 0.59-0.62) for OS in SEER, and 0.62 (95% CI, 0.61-0.63) for OS in NCDB (*P* < .001) (Figure 2A). As before, nomogram risk was significantly prognostic in age-adjusted regression analyses (eFigure 4 in the Supplement) and if nomogram risk was discretized into 4 nomogram-defined risk groups (Figure 2B-D). For both databases, outcomes of patients who had cN1M0 or M1 disease at diagnosis are shown for comparison.

Lastly, we compared the PSMA nomogram with other risk-stratification tools. For this analysis, 139 patients (3%) were excluded from the multi-institutional cohort because of missing information required by the other models: absolute number of positive and negative cores (127 patients) and/or primary Gleason grade (32 patients). Similarly, 3730 patients (16%) were excluded from the SEER cohort for missing T subcategory (3600 patients) and/or primary Gleason grade (145 patients). As the STAR-CAP staging system was recently shown to outperform existing risk-stratification tools, we analyzed the association between PSMA nomogram upstaging risk and STAR-CAP stage and found a significant correlation (eFigure 5 in the Supplement).

**Figure 3.**
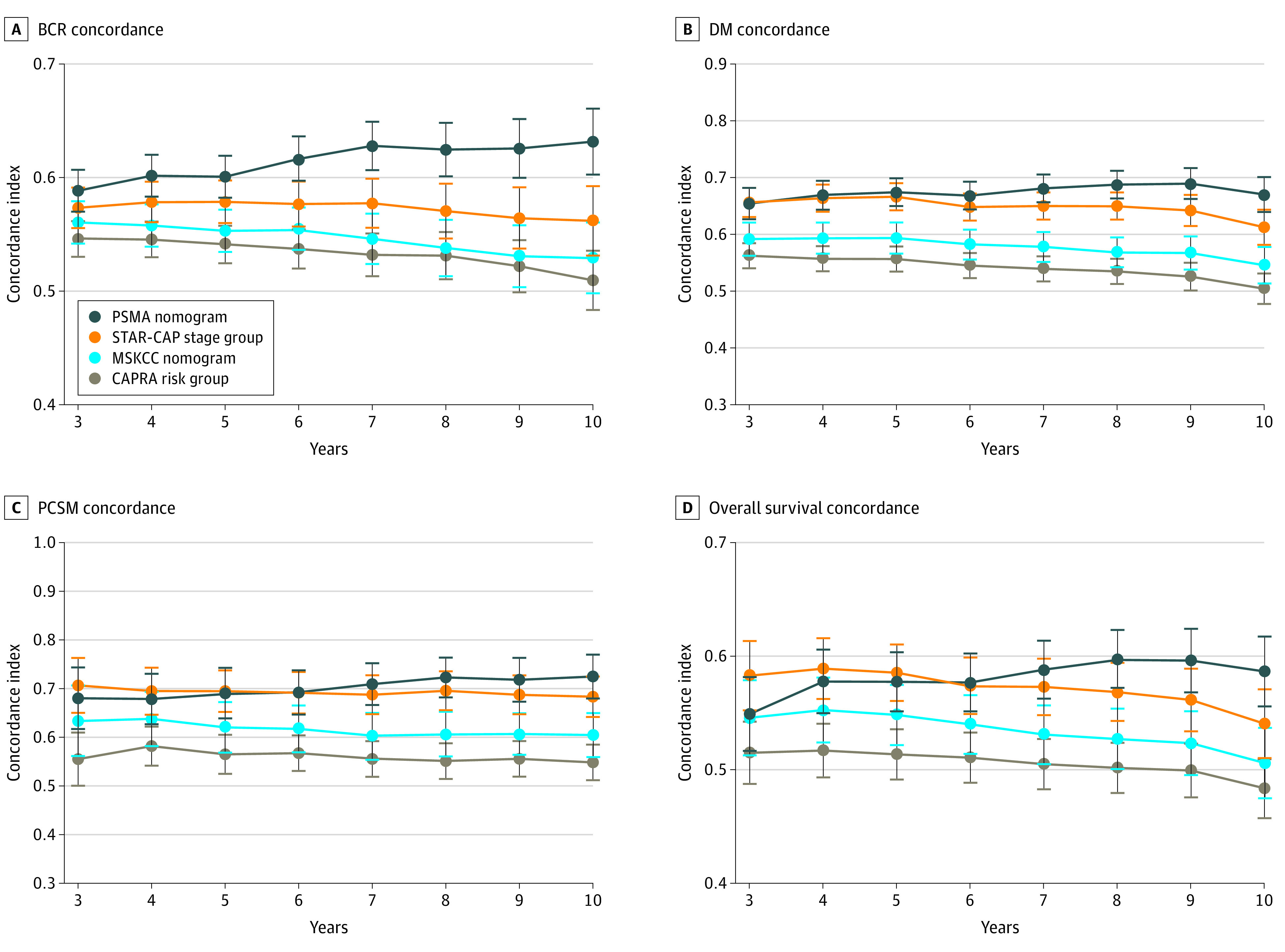
Performance of the Prostate-Specific Membrane Antigen (PSMA) Nomogram, Staging Collaboration for Cancer of the Prostate (STAR-CAP) Stage Groups, Cancer of the Prostate Risk Assessment (CAPRA) Risk Groups, and Memorial Sloan Kettering Cancer Center (MSKCC) Nomogram in the Multi-institutional Cohort, as Assessed by the Concordance Indices End points are biochemical recurrence (BCR) (A), distant metastasis (DM) (B), prostate cancer–specific mortality (PCSM) (C), and overall survival (OS) (D). Error bars represent 95% CIs.

**Figure 4.**
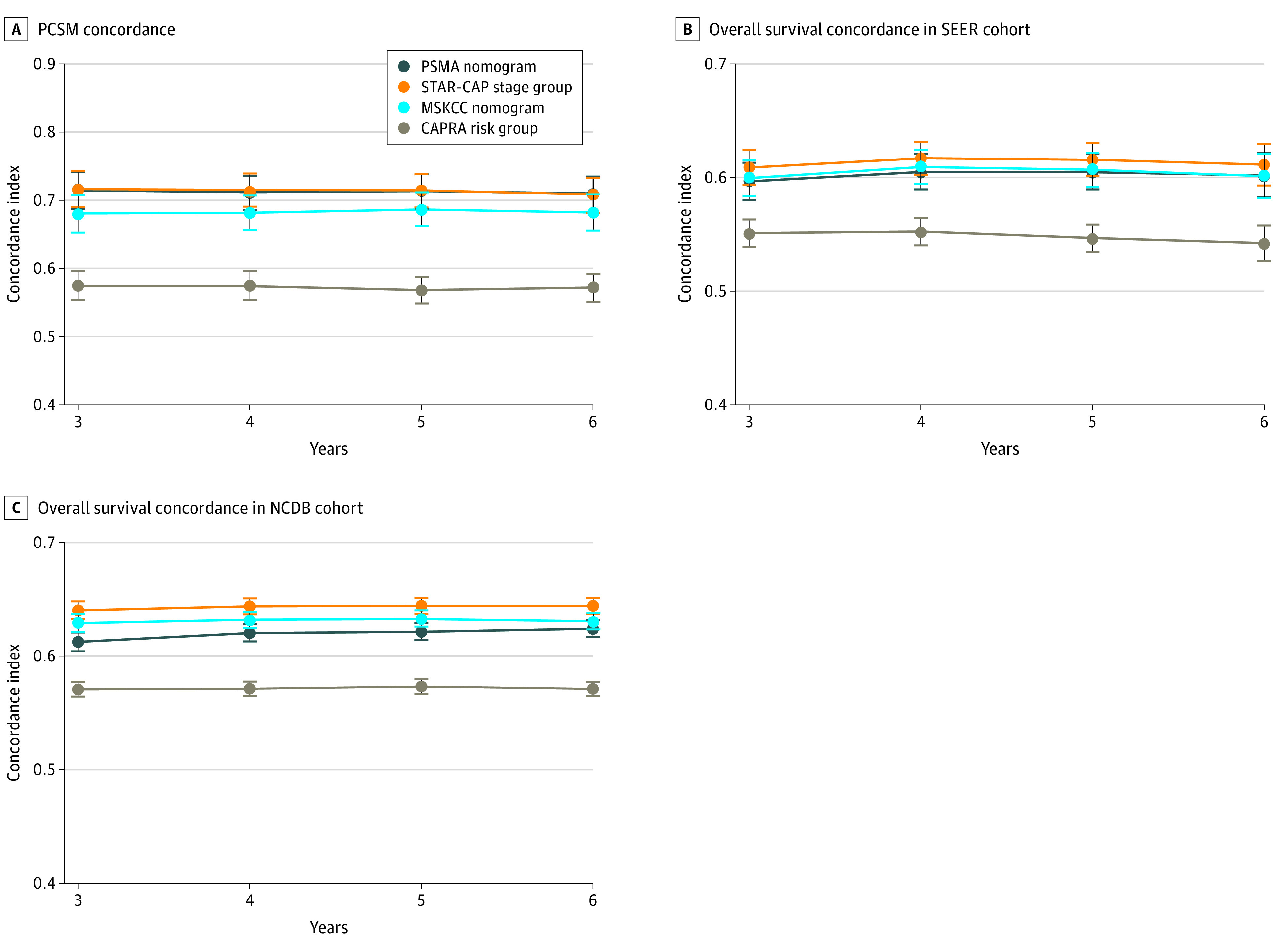
Performance of the Prostate-Specific Membrane Antigen (PSMA) Nomogram, Staging Collaboration for Cancer of the Prostate (STAR-CAP) Stage Groups, Cancer of the Prostate Risk Assessment (CAPRA) Risk Groups, and Memorial Sloan Kettering Cancer Center (MSKCC) Nomogram, as Assessed by the Concordance Indices NCDB indicates National Cancer Database; PCSM, prostate cancer–specific mortality; and SEER, Surveillance, Epidemiology, and End Results. Error bars represent 95% CIs.

Based on the C indices, the PSMA nomogram outperformed STAR-CAP, CAPRA, and the MSKCC nomograms for all end points except PCSM, for which performance was similar to STAR-CAP (Figure 3). PCSM was the end point STAR-CAP was specifically designed to predict. For example, for DM, the 8-year C index for PSMA was 0.69 (95% CI, 0.66-0.71) compared with 0.65 (95% CI, 0.62-0.68) for STAR-CAP (*P* < .001), 0.57 (95% CI, 0.54-0.60) for MSKCC (*P* < .001), and 0.53 (95% CI, 0.51-0.56) for CAPRA (*P* < .001). The PSMA nomogram also performed favorably in the validation cohorts (eg, 5-year C indices for PCSM: PSMA, 0.71 [95% CI, 0.69-0.74] vs STAR-CAP, 0.72 [95% CI, 0.69-0.74]; *P* = .88; vs MSKCC, 0.69 [95% CI, 0.66-0.71]; *P* = .01; vs CAPRA, 0.57 [95% CI, 0.55-0.59]; *P* < .001) (Figure 4) and if the IPAs were used (eFigure 6 and eFigure 7 in the Supplement). In subgroup analyses stratified by treatment type, performance of the PSMA nomogram was generally comparable with STAR-CAP and superior to the other models (eFigures 8-12 in the Supplement). Model performance was also evaluated using calibration plots (eFigure 13 in the Supplement).

## Discussion

To our knowledge, this is the first study to evaluate the association of estimated risk of upstaging on PSMA PET/CT with long-term clinical outcomes (DM and PCSM) in patients with localized disease by conventional imaging. Direct, prospective evidence between PSMA PET/CT and clinically important end points is still at least several years away. Until such data become available, our PSMA nomogram serves as a proxy for the long-term prognostic and clinical significance of PSMA PET/CT findings. Furthermore, as the nomogram was specifically tuned to nonlocalized disease detected on PSMA PET/CT that was occult on conventional imaging, the superior prognostic discrimination of the nomogram suggests that such previously occult, nonlocalized disease may be the primary driver of outcomes in this high-risk patient population. The external validity of our results is supported by the inclusion of 15 institutions in the primary cohort and by the external validation in the registry-based cohorts.

Additionally, the PSMA nomogram showed improved risk stratification, outperforming all other models for all end points, except PCSM, for which it had similar performance as STAR-CAP. Although the C indices of 0.69 to 0.71 for DM and PCSM may appear to be middling, they were nonetheless superior to the other models, and prognostication in high-risk patients is especially challenging because of the heterogeneity in this population.^1,2^ Importantly, the PSMA nomogram was trained purely on radiographic findings on PSMA PET/CT at initial diagnosis, with no input from downstream clinical outcomes. By contrast, the comparison models were trained directly on actual clinical end points, such as PCSM in the case of STAR-CAP. It is noteworthy that the PSMA nomogram provided superior risk discrimination vs existing risk-stratification tools that were designed specifically to predict clinical outcomes, which may speak to the significance of PSMA PET/CT findings as a marker of overall disease course.

Our results support growing data for the clinical utility of PSMA PET/CT in the initial evaluation and management of patients with high-risk prostate cancer. Recently, the landmark proPSMA randomized trial showed that PSMA PET/CT was more sensitive and specific than conventional imaging for nodal and distant metastatic disease in patients with high- and very high-risk prostate cancer.^7^ Furthermore, 2 recent studies^20,21^ reported an association between lymph node positivity on preoperative PSMA PET/CT and persistently elevated PSA level or BCR after RP, although no studies to date that we are aware of have reported on the association between PSMA PET/CT and long-term outcomes such as DM or PCSM. Although the utility of PSMA PET/CT is increasingly apparent, access (logistical and financial) remains a barrier to many patients. Thus, we implemented the PSMA nomogram as an online calculator.^9^

Interestingly, the PSMA nomogram was more prognostic for DM and PCSM than BCR, perhaps because the nomogram was trained on regional and distant PET/CT findings, whereas BCR could be due to any combination of local, regional, or distant failure. All models were less accurate for OS, given that most deaths are not due to prostate cancer but rather competing causes for mortality, such as cardiovascular disease.^22^ Performance of all models was lower for patients receiving EBRT plus BT, possibly reflecting smaller sample sizes and fewer events, as seen by the wider confidence intervals. Alternatively, outcomes after this treatment modality may be influenced by as-yet unmeasured variables, or EBRT plus BT may be associated with patterns of failure in a way that is not yet accurately modeled by current information.

More precise risk stratification may enable more personalized treatment. In the proPSMA trial, PSMA PET/CT led to more changes in management compared with conventional imaging (28% vs 15%; *P* = .008). In our study, the prognosis of patients with high PSMA nomogram risk was intermediate between patients with more favorable (lower nomogram risk) disease and patients with overt N1 or M1 disease. These findings again suggest that occult, nonlocalized disease may be the main driver of outcomes, and treatment intensification may be warranted in such patients. As proof of concept, escalated therapy has been associated with improved outcomes in very high-risk patients, eg, combination EBRT plus BT and ADT in men with Gleason score 9 to 10 prostate cancer^23^ and use of longer ADT durations in higher-grade disease.^24^ It is logical to consider ways of intensifying therapy (eg, advanced ADT as in the Systemic Therapy in Advancing or Metastatic Prostate Cancer: Evaluation of Drug Efficacy trial)^25^ for patients with high PSMA nomogram risk, but prospective validation is required.

### Limitations

This study has limitations. First, the PSMA nomogram is a proxy for upstaging on PSMA PET/CT; it does not represent the patients’ actual findings on PSMA PET/CT, although the accuracy of the nomogram (AUC, 0.75) suggests PSMA PET/CT is itself prognostic. Second, our study is retrospective; specific procedures were not standardized, and there was no central pathology review. Despite these limitations, our results reflect actual patterns of care, and the PSMA nomogram was significantly prognostic across multiple institutional and real-world settings. Third, the number of patients with follow-up longer than 10 years (in the multi-institutional cohort) or 5 years (in SEER and NCDB) was low. Fourth, molecular and genomic data were not available. Integration of such information with the PSMA nomogram may further improve risk discrimination, and this is an area of future study.

## Conclusions

In this study, the estimated probability of nonlocalized upstaging on PSMA PET/CT was significantly prognostic of long-term, clinically meaningful end points, and the nomogram performed favorably vs existing risk-stratification tools. This was true despite the PSMA nomogram being trained purely on radiographic findings at initial diagnosis, while the comparison models were specifically designed to predict clinical end points. Previously occult, PSMA PET/CT–detected disease may be the main driver of outcomes in high-risk patients. Prospective investigation centered on making use of the PSMA nomogram as part of risk-adapted treatment strategy is warranted.

## Notes

Supplement. **eMethods.** Description of Stepwise Method to Identify Nomogram Cut Points **eTable 1.** Selection Procedure to Identify Patients for the Multi-institutional Cohort **eTable 2.** Repeated 10-fold Cross-validation of Nomogram Cut Points With 100 Repeats **eTable 3.** Bootstrap Validation of Nomogram Cut Points with 1000 Repeats **eTable 4.** Selection Procedure to Identify Patients for the SEER Cohort **eTable 5.** Selection Procedure to Identify Patients for the NCDB Cohort **eFigure 1.** Concordance Indices of the PSMA Nomogram in the Multi-institutional Cohort Stratified by Treatment Modality **eFigure 2.** Time-Dependent Decision Curve Analysis Graphs **eFigure 3.** Forest Plot of Age-Adjusted Hazard Ratios and Subdistribution Hazard Ratios per 10% Increase in Nomogram Risk in the Multi-institutional Cohort **eFigure 4.** Forest Plot of Age-Adjusted Hazard Ratios and Subdistribution Hazard Ratios per 10% Increase in Nomogram Risk in the Registry-Based (SEER and NCDB) Cohorts **eFigure 5.** Distribution of PSMA Nomogram Upstage Risk According to STAR-CAP Stage Groups **eFigure 6.** Index of Prediction Accuracy (IPA) for the PSMA Nomogram and Other Models (STAR-CAP, CAPRA, and MSKCC Nomogram) in the Multi-institutional Cohort **eFigure 7.** Index of Prediction Accuracy (IPA) for the PSMA Nomogram and Other Models in the Registry-Based (SEER and NCDB) Cohorts **eFigure 8.** Performance of the PSMA Nomogram and Other Models in the Multi-institutional Cohort for Patients Treated With Radical Prostatectomy **eFigure 9.** Performance of the PSMA Nomogram and Other Models in the Multi-institutional Cohort for Patients Treated With External Beam Radiation **eFigure 10.** Performance of the PSMA Nomogram and Other Models in the Multi-institutional Cohort for Patients Treated With External Beam Radiation Plus Brachytherapy **eFigure 11.** Performance of the PSMA Nomogram and Other Models in the SEER Cohort, Further Stratified by Type of Treatment **eFigure 12.** Performance of the PSMA Nomogram and Other Models in the NCDB Cohort, Further Stratified by Type of Treatment **eFigure 13.** Calibration Plots for the PSMA Nomogram and the Other Models
