# Supplementary material for: Performance of a Prostate-Specific Membrane Antigen Positron Emission Tomography/Computed Tomography–Derived Risk-Stratification Tool for High-risk and Very High-risk Prostate Cancer
Source: JAMA Netw Open. 2021 Dec 13;4(12):e2138550. doi: 10.1001/jamanetworkopen.2021.38550 (PMC8669522; doi:10.1001/jamanetworkopen.2021.38550)
Supplement: Supplement. — eMethods. Description of Stepwise Method to Identify Nomogram Cut Points eTable 1. Selection Procedure to Identify Patients for the Multi-institutional Cohort eTable 2. Repeated 10-fold Cross-validation of Nomogram Cut Points With 100 Repeats eTable 3. Bootstrap Validation of Nomogram Cut Points with 1000 Repeats eTable 4. Selection Procedure to Identify Patients for the SEER Cohort eTable 5. Selection Procedure to Identify Patients for the NCDB Cohort eFigure 1. Concordance Indices of the PSMA Nomogram in the Multi-institutional Cohort Stratified by Treatment Modality eFigure 2. Time-Dependent Decision Curve Analysis Graphs eFigure 3. Forest Plot of Age-Adjusted Hazard Ratios and Subdistribution Hazard Ratios per 10% Increase in Nomogram Risk in the Multi-institutional Cohort eFigure 4. Forest Plot of Age-Adjusted Hazard Ratios and Subdistribution Hazard Ratios per 10% Increase in Nomogram Risk in the Registry-Based (SEER and NCDB) Cohorts eFigure 5. Distribution of PSMA Nomogram Upstage Risk According to STAR-CAP Stage Groups eFigure 6. Index of Prediction Accuracy (IPA) for the PSMA Nomogram and Other Models (STAR-CAP, CAPRA, and MSKCC Nomogram) in the Multi-institutional Cohort eFigure 7. Index of Prediction Accuracy (IPA) for the PSMA Nomogram and Other Models in the Registry-Based (SEER and NCDB) Cohorts eFigure 8. Performance of the PSMA Nomogram and Other Models in the Multi-institutional Cohort for Patients Treated With Radical Prostatectomy eFigure 9. Performance of the PSMA Nomogram and Other Models in the Multi-institutional Cohort for Patients Treated With External Beam Radiation eFigure 10. Performance of the PSMA Nomogram and Other Models in the Multi-institutional Cohort for Patients Treated With External Beam Radiation Plus Brachytherapy eFigure 11. Performance of the PSMA Nomogram and Other Models in the SEER Cohort, Further Stratified by Type of Treatment eFigure 12. Performance of the PSMA Nomogram and Other Models in the NCDB Cohort, Further Str [file jamanetwopen-e2138550-s001.pdf]

Supplemental Online Content

Xiang M, Ma TM, Savjani R, et al. Performance of a prostate-specific membrane antigen positron emission tomography/computed tomography–derived risk-stratification tool for high-risk and very high-risk prostate cancer. *JAMA Netw Open.* 2021;4(12):e2138550. doi:10.1001/jamanetworkopen.2021.38550

eMethods. Description of Stepwise Method to Identify Nomogram Cut Points

eTable 1. Selection Procedure to Identify Patients for the Multi-institutional Cohort

eTable 2. Repeated 10-fold Cross-validation of Nomogram Cut Points With 100 Repeats

eTable 3. Bootstrap Validation of Nomogram Cut Points with 1000 Repeats

eTable 4. Selection Procedure to Identify Patients for the SEER Cohort

eTable 5. Selection Procedure to Identify Patients for the NCDB Cohort

eFigure 1. Concordance Indices of the PSMA Nomogram in the Multi-institutional Cohort Stratified by Treatment Modality

eFigure 2. Time-Dependent Decision Curve Analysis Graphs

eFigure 3. Forest Plot of Age-Adjusted Hazard Ratios and Subdistribution Hazard Ratios per 10% Increase in Nomogram Risk in the Multi-institutional Cohort

eFigure 4. Forest Plot of Age-Adjusted Hazard Ratios and Subdistribution Hazard Ratios per 10% Increase in Nomogram Risk in the Registry-Based (SEER and NCDB) Cohorts

eFigure 5. Distribution of PSMA Nomogram Upstage Risk According to STAR-CAP Stage Groups

eFigure 6. Index of Prediction Accuracy (IPA) for the PSMA Nomogram and Other Models (STAR-CAP, CAPRA, and MSKCC Nomogram) in the Multi-institutional Cohort

eFigure 7. Index of Prediction Accuracy (IPA) for the PSMA Nomogram and Other Models in the Registry-Based (SEER and NCDB) Cohorts

eFigure 8. Performance of the PSMA Nomogram and Other Models in the Multi-institutional Cohort for Patients Treated With Radical Prostatectomy

eFigure 9. Performance of the PSMA Nomogram and Other Models in the Multi-institutional Cohort for Patients Treated With External Beam Radiation

eFigure 10. Performance of the PSMA Nomogram and Other Models in the Multi-institutional Cohort for Patients Treated With External Beam Radiation Plus Brachytherapy

eFigure 11. Performance of the PSMA Nomogram and Other Models in the SEER Cohort, Further Stratified by Type of Treatment

eFigure 12. Performance of the PSMA Nomogram and Other Models in the NCDB Cohort, Further Stratified by Type of Treatment

eFigure 13. Calibration Plots for the PSMA Nomogram and the Other Models

© 2021 Xiang M et al. *JAMA Network Open.*

This supplemental material has been provided by the authors to give readers additional information about their work.

© 2021 Xiang M et al. *JAMA Network Open.*

eMethods. *Description of Stepwise Method to Identify Nomogram Cut Points*

Since the nomogram outputs a continuous score between 0 and 1, we sought to identify cutpoint(s) that could be used to partition patients into discrete risk groups based on their nomogram score. There are several advantages to identifying cutpoints. First, clinicians may find it helpful to use the cutpoints as a tangible gauge of a patient's level of risk. Second, time-to-event outcomes may be compared between the different risk groups (i.e., Figures 1-2). Third, discrete risk stratification may aid in the design of future studies or clinical trials.

To identify cutpoints, we utilized the endpoint of 8-year distant metastasis (DM). This was chosen based on the median follow-up duration, the clinical implications of DM, and the greater number of DM events compared to prostate cancer-specific mortality (PCSM). The first cutpoint was identified as the point on the receiver operating characteristic (ROC) curve that minimized the distance to the upper left-hand corner. The ROC curve was determined using the R package *timeROC* (version 0.4), and then smoothed in MATLAB using the Loess method in the built-in function *smooth*. The first cutpoint (i.e., 0.14) was used to partition the cohort into two subdivisions: patients whose nomogram score was less than or equal to the cutpoint comprised the lower subdivision, and patients whose nomogram score was greater than the cutpoint comprised the upper subdivision.

This process was then continued iteratively on each subdivision to identify additional cutpoints until the nomogram was no longer prognostic, as defined by the 95% confidence interval of the C-index overlapping with 0.50 (i.e., not significantly different from chance). At each step, within the lower subdivision, the nomogram was not significantly prognostic, indicating it was not productive to further subdivide it. On the other hand, within the upper subdivision (comprising patients whose nomogram score was greater than the latest cutpoint), the nomogram continued to be significantly prognostic, and additional cutpoints were added, until finally the nomogram was no longer prognostic after the 3rd cutpoint (i.e., 0.41), indicating it was not productive to add further cutpoints. Thus, the procedure self-

© 2021 Xiang M et al. *JAMA Network Open.*

terminated after a total of 3 iterations, identifying 3 cutpoints (0.14, 0.27, 0.41) that partitioned patients into 4 nomogram-defined risk groups.

Finally, the robustness of the cutpoints identified using this stepwise method was analyzed using repeated tenfold cross-validation with 100 repeats, and bootstrap validation with 1,000 repeats, as described in the Methods and eTables 2-3.

© 2021 Xiang M et al. *JAMA Network Open.*

eTable 1. Selection Procedure to Identify Patients for the Multi-institutional Cohort

	**Number of patients**
All patients with NCCN high-risk prostate adenocarcinoma collated from 15 tertiary referral centers	6808
With known PSA, Gleason score, T stage, percent positive cores	6808
With known follow-up duration and vital status	6797
With known status of biochemical recurrence	6597
With known status of distant metastasis	5395
Alive at last follow-up, or with known status of prostate-cancer specific mortality if deceased	5275

© 2021 Xiang M et al. *JAMA Network Open.*

eTable 2. Repeated 10-fold Cross-validation of Nomogram Cut Points With 100 Repeats

Repeated tenfold cross-validation of nomogram cutpoint determination using the endpoint of 8-year distant metastasis (DM). We performed 100 repeats, for a total of 1,000 training and validation sets. Concordance (C-) index is in the validation sets using cutpoints derived from the training sets. Mean and empirical confidence intervals (CI) are across all folds across all repeats.

	**Cutpoint 1**	**Cutpoint 2**	**Cutpoint 3**	**8-yr C-index for DM**
**Mean**	13.7	26.7	40.6	0.67
**95% CI**	13.0-14.0	25.8-27.5	38.9-42.1	0.61-0.74

© 2021 Xiang M et al. *JAMA Network Open.*

eTable 3. Bootstrap Validation of Nomogram Cut Points with 1000 Repeats

Bootstrap validation of nomogram cutpoint determination using the endpoint of 8-year distant metastasis (DM). Results are for 1,000 bootstrapped training and validation sets. Bootstrapped training samples were the same size as the original dataset, and validation samples were the out-of-bag cases for each training sample. Concordance (C-) index is in the validation sets using cutpoints derived from the training sets. Mean and empirical confidence intervals (CI) are across all 1,000 bootstrapped samples.

	**Cutpoint 1**	**Cutpoint 2**	**Cutpoint 3**	**8-yr C-index for DM**
**Mean**	13.9	27.2	40.7	0.67
**95% CI**	12.4-16.3	24.4-30.6	37.0-44.4	0.65-0.70

© 2021 Xiang M et al. *JAMA Network Open.*

eTable 4. Selection Procedure to Identify Patients for the SEER Cohort

	**Number of patients**
All prostate cancer patients in SEER diagnosed 2010-2016	368287
With known follow-up duration and vital status	368287
Adenocarcinoma histology	353859
Node-negative and non-metastatic (N0, M0)	294022
With known PSA	253861
With known Gleason score	247384
With known T stage	243393
NCCN high-risk based on PSA, Gleason, T stage	63887
With known number of positive biopsy cores and total biopsy cores	41776
Alive at last follow-up, or with known status of prostate-cancer specific mortality if deceased	41776
Not treated with radical prostatectomy (see text for rationale for exclusion)	23989

© 2021 Xiang M et al. *JAMA Network Open.*

eTable 5. Selection Procedure to Identify Patients for the NCDB Cohort

	**Number of patients**
All prostate cancer patients in the NCDB diagnosed 2010-2016	769256
With known follow-up duration and vital status	769172
Adenocarcinoma histology	749508
Node-negative and non-metastatic (N0, M0)	626120
With known PSA	561977
With known Gleason score	535891
With known T stage	509937
NCCN high-risk based on PSA, Gleason, T stage	118924
With known number of positive biopsy cores and total biopsy cores	88909

© 2021 Xiang M et al. *JAMA Network Open.*

eFigure 1. Concordance Indices of the PSMA Nomogram in the Multi-institutional Cohort Stratified by Treatment Modality

Concordance indices (C-indices) of the PSMA nomogram in the multi-institutional cohort for the endpoints of A) biochemical recurrence (BCR), B) distant metastasis (DM), C) prostate cancer-specific mortality (PCSM), and D) overall survival (OS), stratified by treatment modality: radical prostatectomy (RP), external beam radiation (EBRT), or EBRT and brachytherapy (EBRT+BT). Error bars represent 95% confidence intervals (CI).

A)

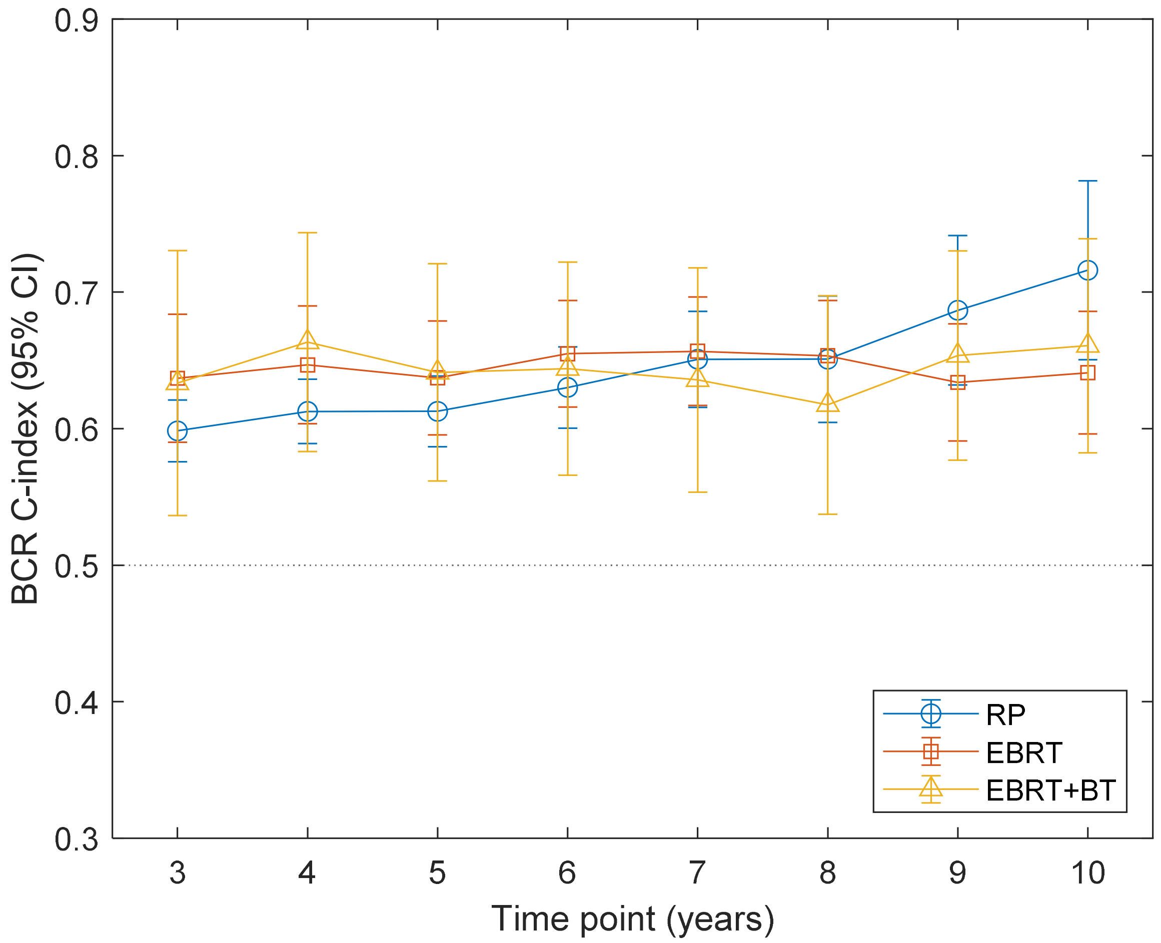

© 2021 Xiang M et al. *JAMA Network Open.*

B)

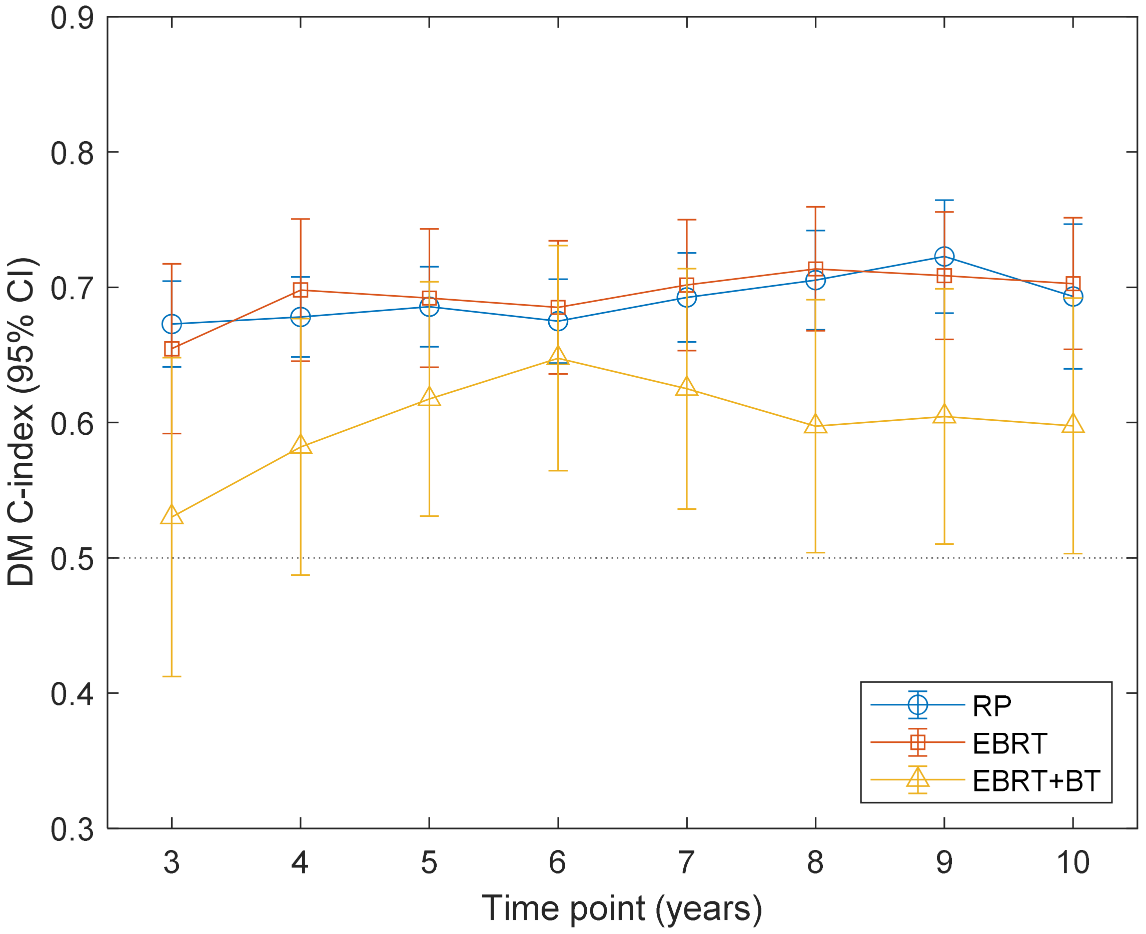

© 2021 Xiang M et al. *JAMA Network Open.*

C)

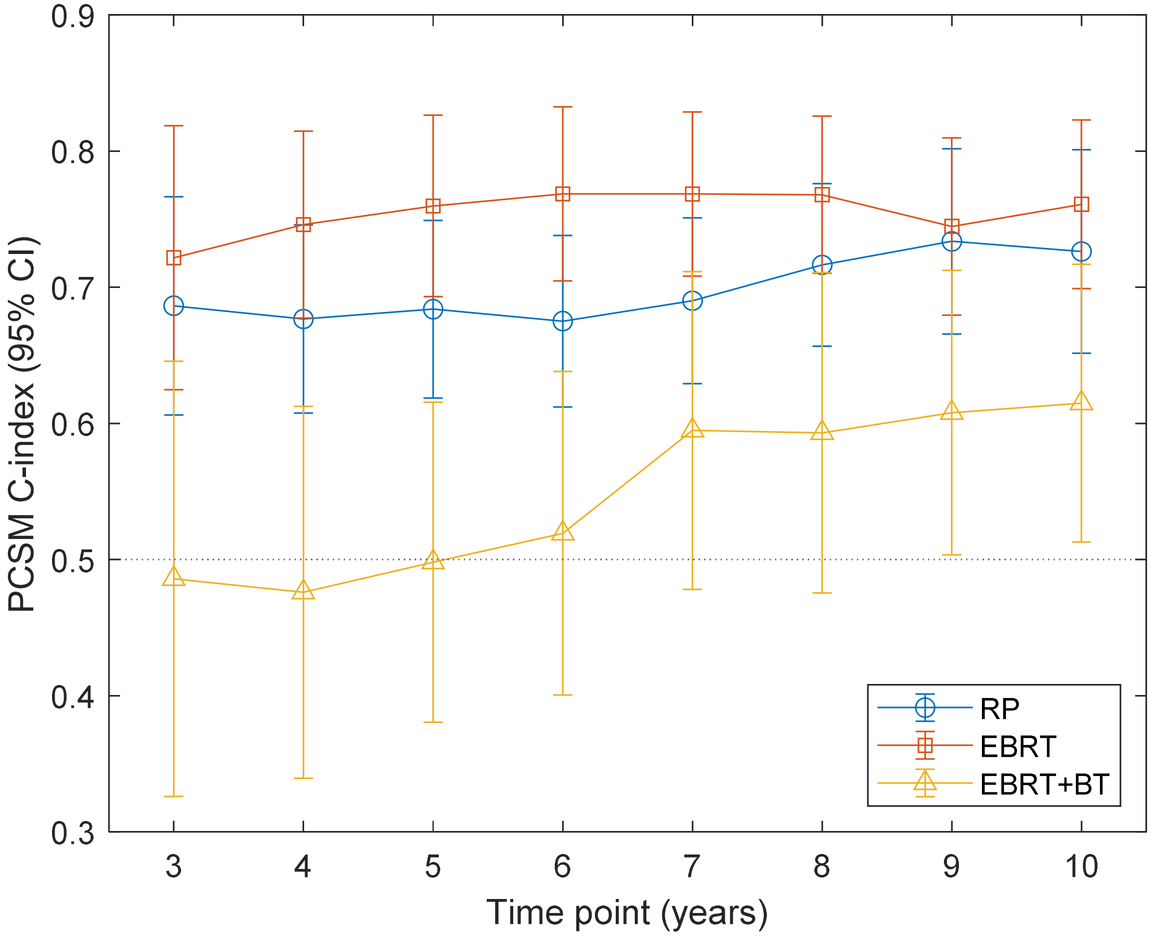

© 2021 Xiang M et al. *JAMA Network Open.*

D)

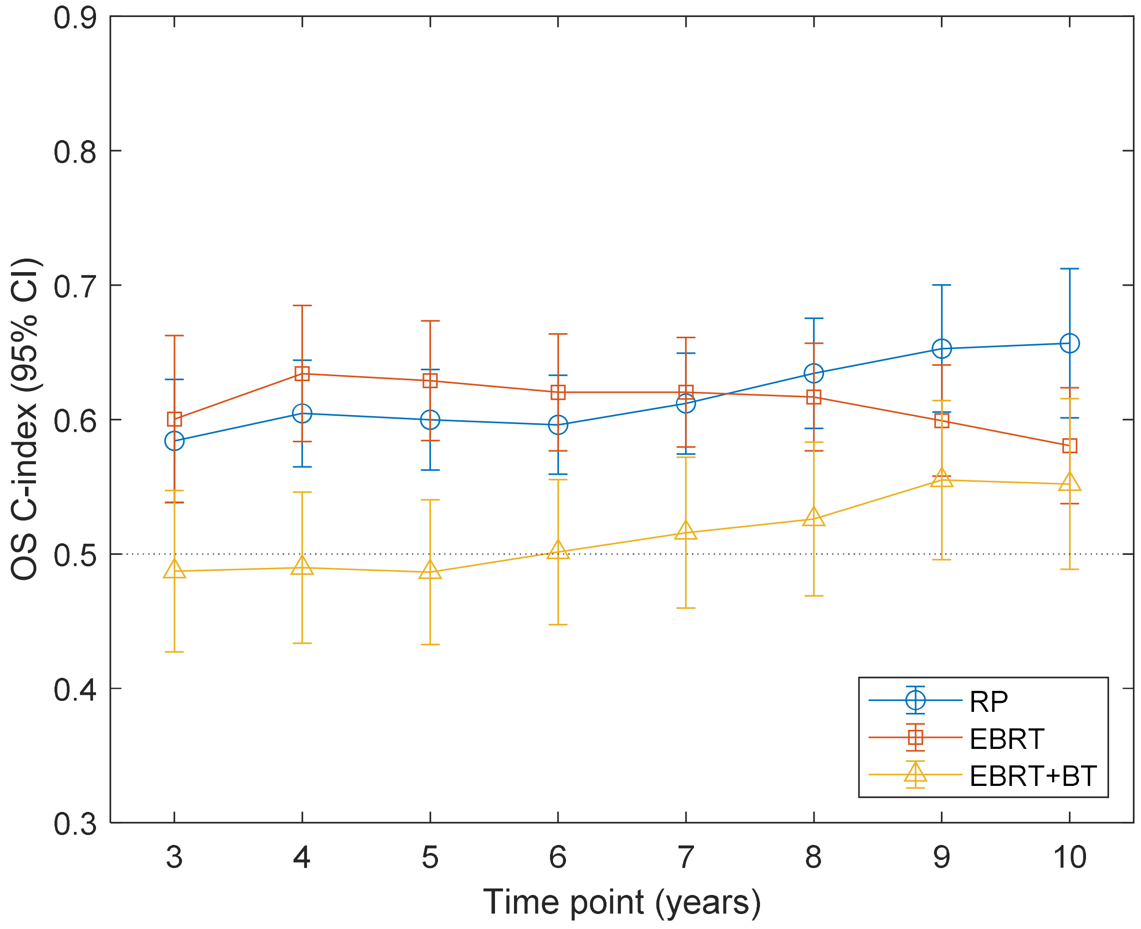

© 2021 Xiang M et al. *JAMA Network Open.*

eFigure 2. Time-Dependent Decision Curve Analysis Graphs

Time-dependent decision curve analysis graphs of the PSMA nomogram for the outcomes of A) biochemical recurrence (BCR), B) distant metastasis (DM), C) prostate cancer-specific mortality (PCSM), and D) overall survival (OS).

A)

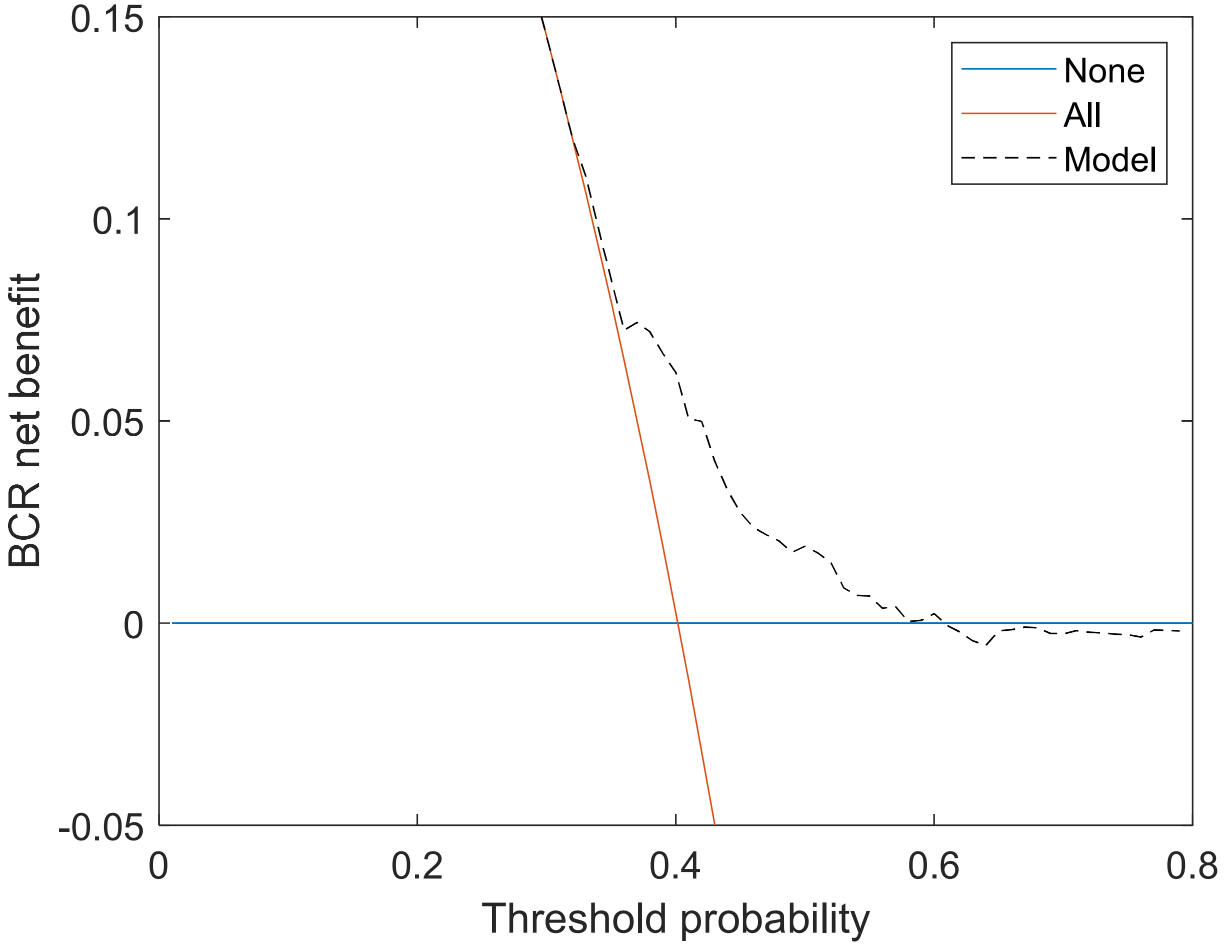

© 2021 Xiang M et al. *JAMA Network Open.*

B)

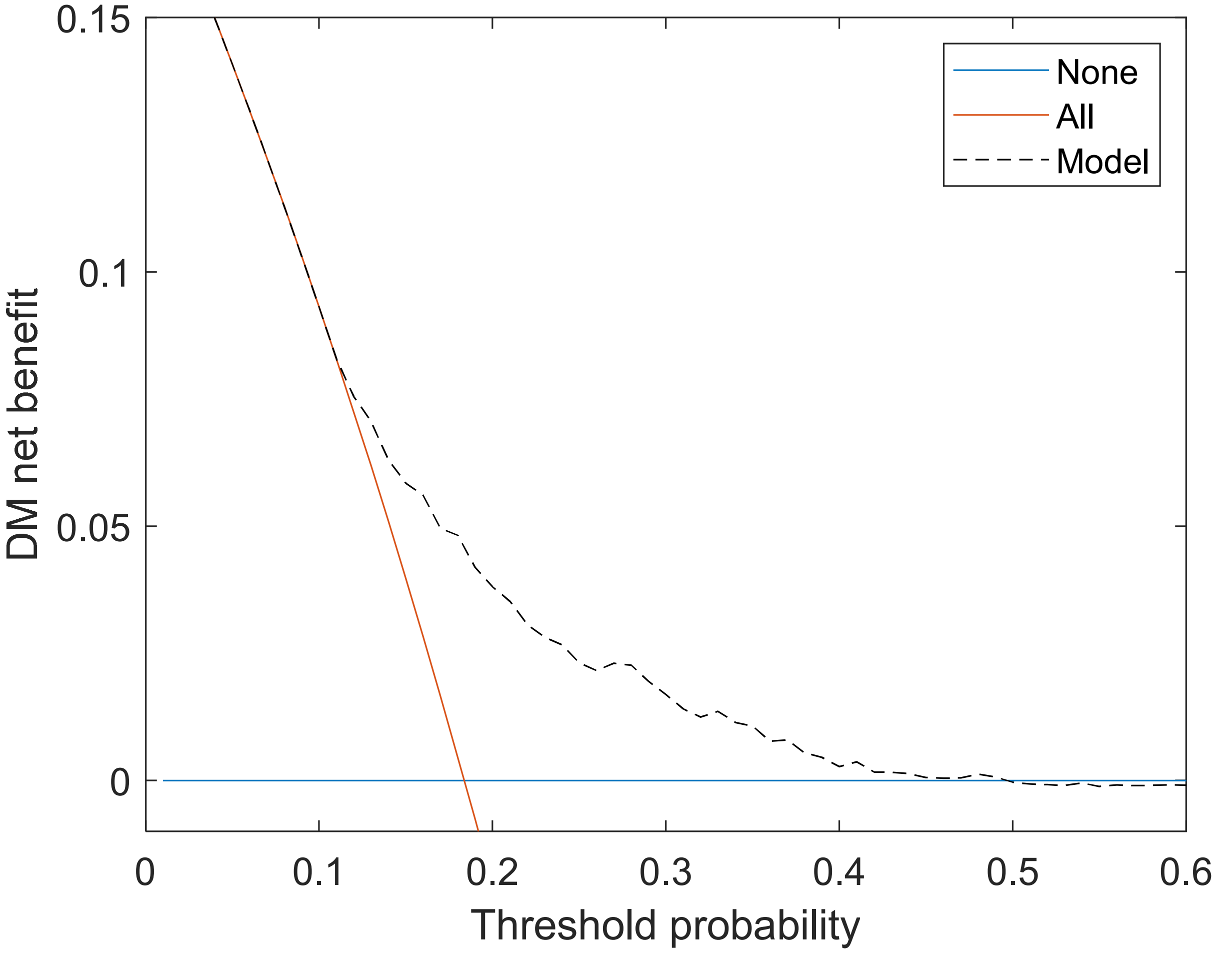

© 2021 Xiang M et al. *JAMA Network Open.*

C)

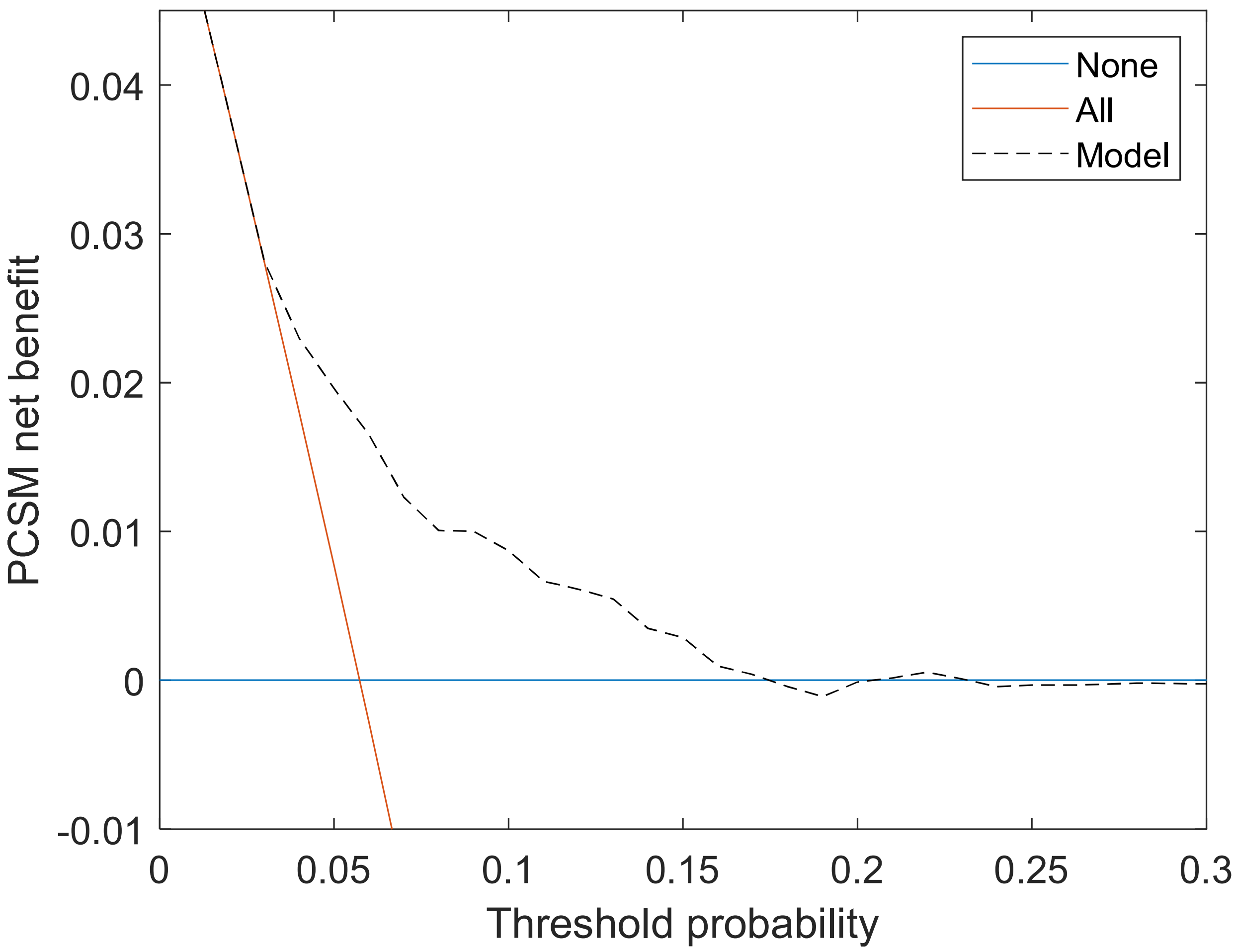

© 2021 Xiang M et al. *JAMA Network Open.*

D)

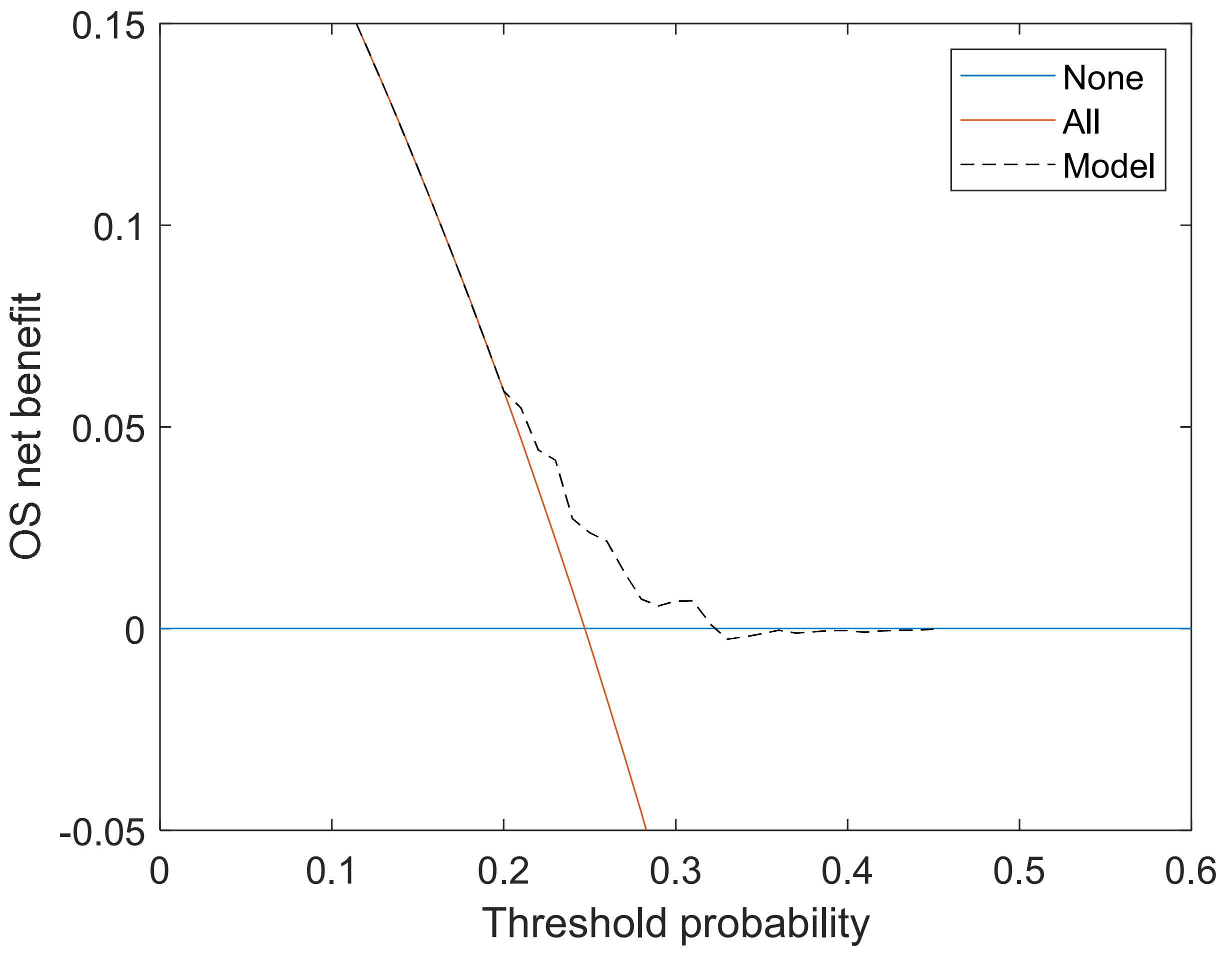

© 2021 Xiang M et al. *JAMA Network Open.*

eFigure 3. Forest Plot of Age-Adjusted Hazard Ratios and Subdistribution Hazard Ratios per 10% Increase in Nomogram Risk in the Multi-institutional Cohort

Forest plot of age-adjusted hazard ratios (HR) and subdistribution hazard ratios (sHR) for A) biochemical recurrence (BCR), B) distant metastasis (DM), C) prostate cancer-specific mortality (PCSM), and D) overall survival (OS) per 10% increase in nomogram risk, stratified by type of treatment (RP, radical prostatectomy; EBRT, external beam radiation; BT, brachytherapy). Error bars and numbers in parentheses represent 95% confidence intervals. Point estimates and p-values of each result are provided to the right of the panel. Marker sizes are proportional to the relative number of patients.

A)

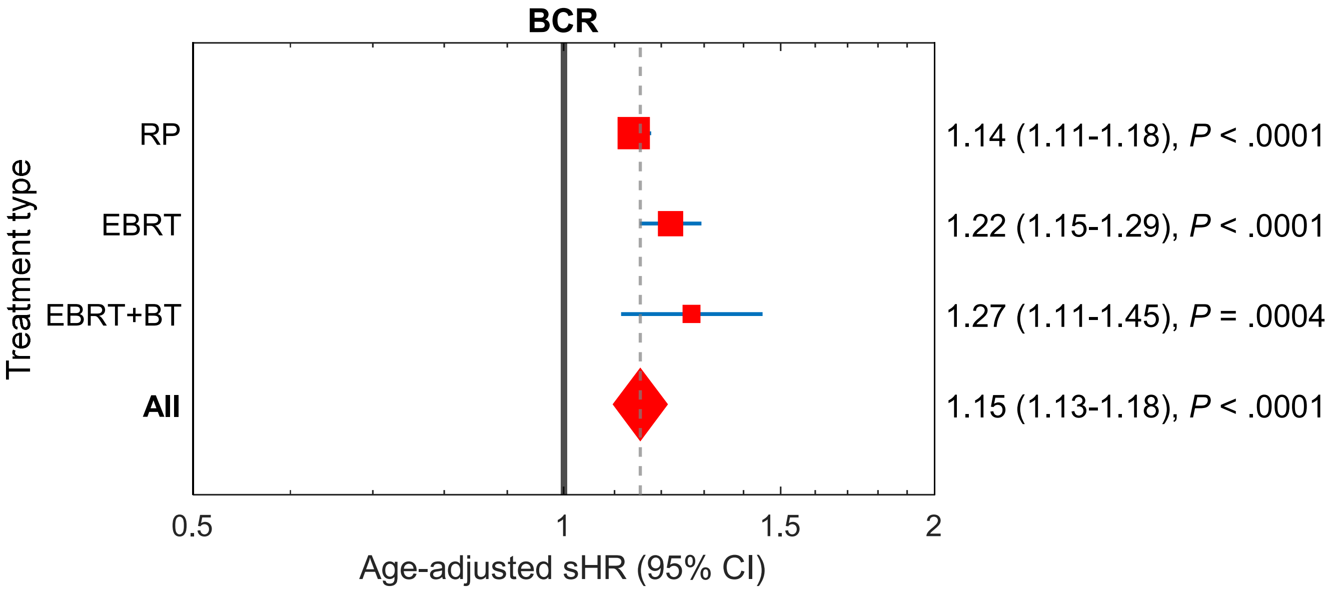

© 2021 Xiang M et al. *JAMA Network Open.*

B)

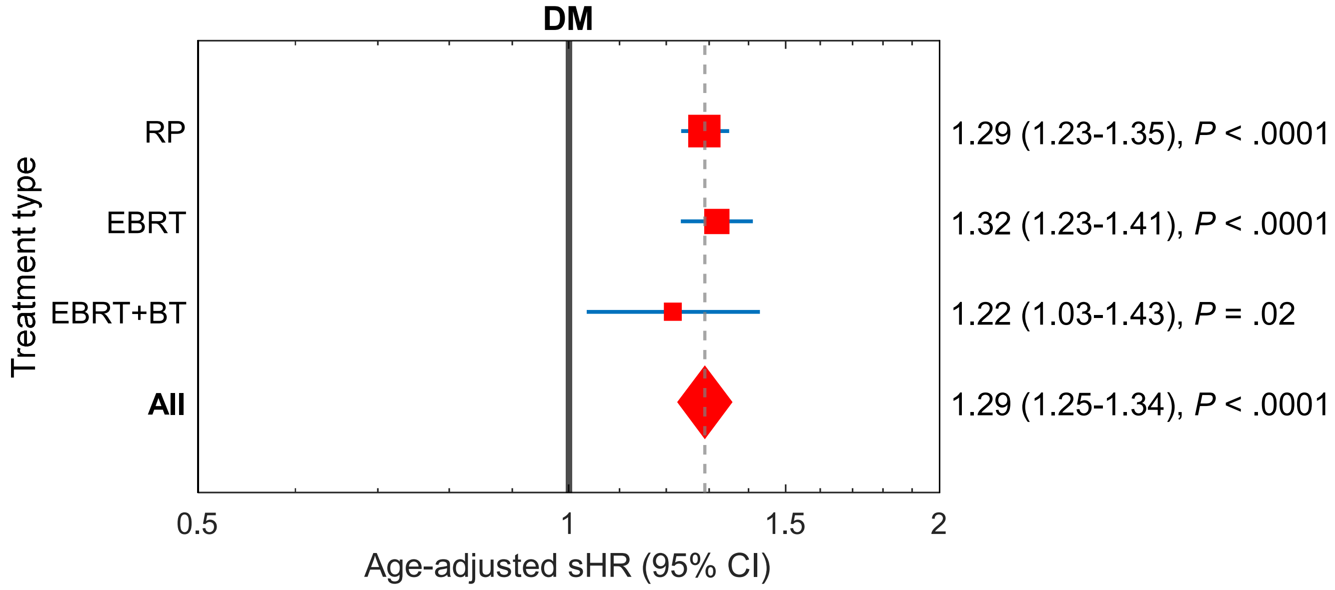

© 2021 Xiang M et al. *JAMA Network Open.*

C)

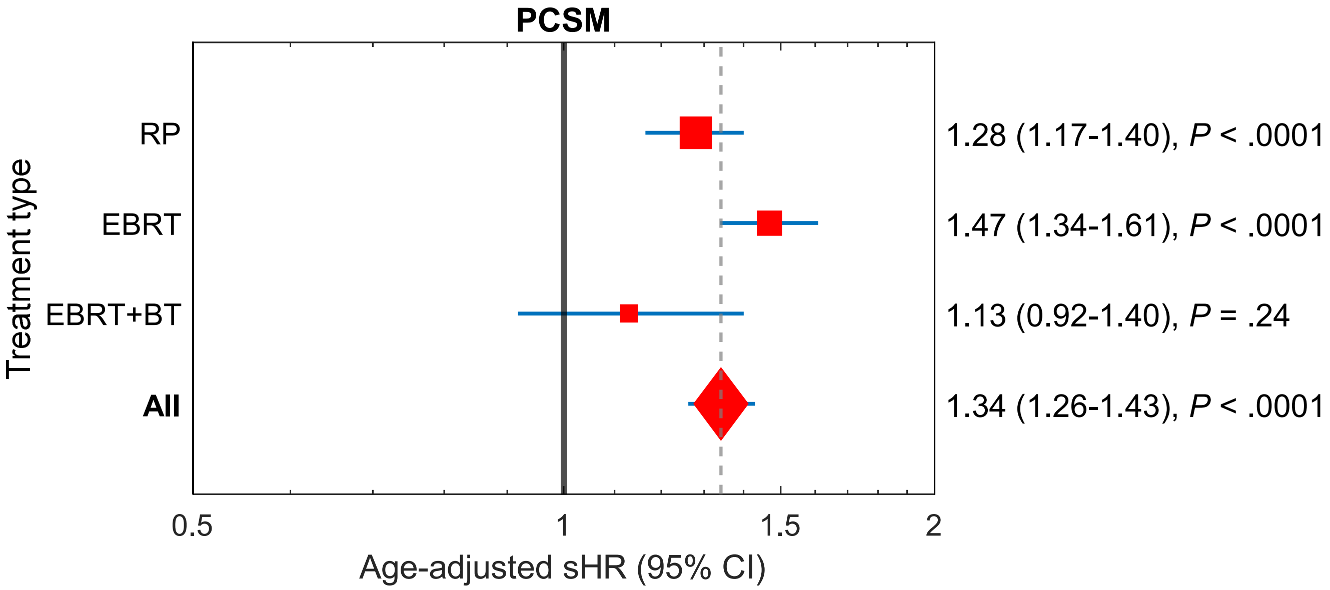

© 2021 Xiang M et al. *JAMA Network Open.*

D)

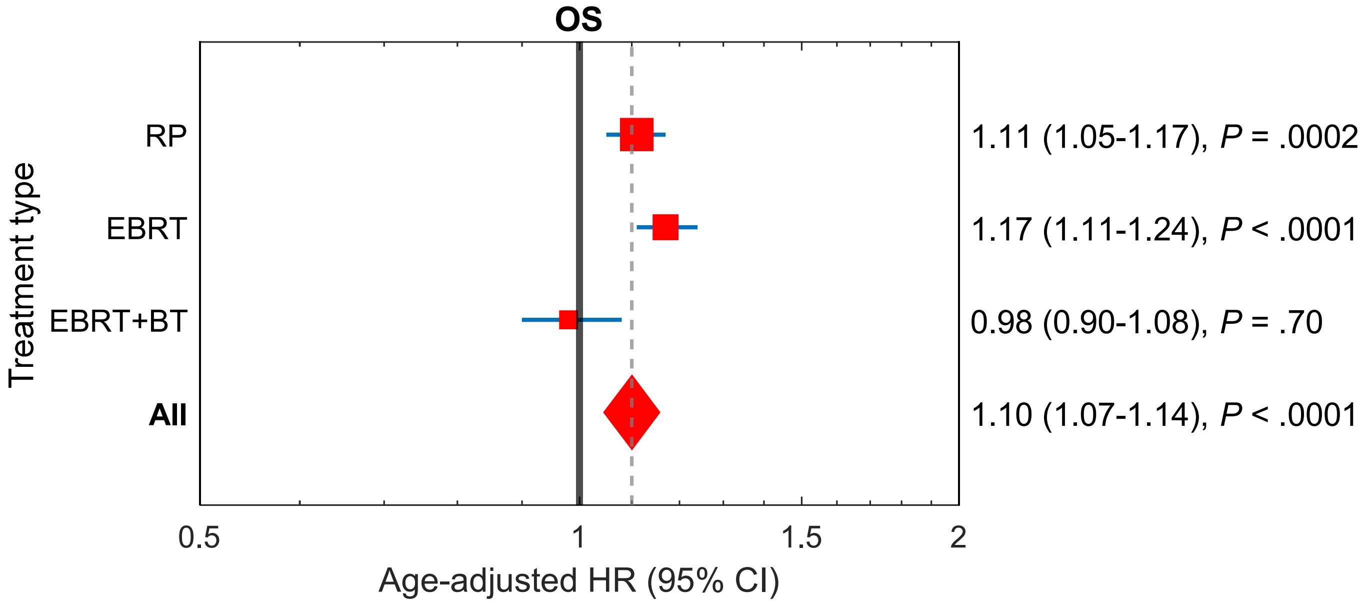

© 2021 Xiang M et al. *JAMA Network Open.*

eFigure 4. Forest Plot of Age-Adjusted Hazard Ratios and Subdistribution Hazard Ratios per 10% Increase in Nomogram Risk in the Registry-Based (SEER and NCDB) Cohorts

Forest plot of age-adjusted hazard ratios (HR) and subdistribution hazard ratios (sHR) for prostate cancer-specific mortality (PCSM) and overall survival (OS) per 10% increase in nomogram risk, stratified by type of treatment (RP, radical prostatectomy; EBRT, external beam radiation; BT, brachytherapy) in the SEER cohort (panels A-B) and the NCDB cohort (panel C). Patients receiving RP in SEER were excluded (see text). Error bars and numbers in parentheses represent 95% confidence intervals. Point estimates and p-values of each result are provided to the right of the panel. Marker sizes are proportional to the relative number of patients.

A)

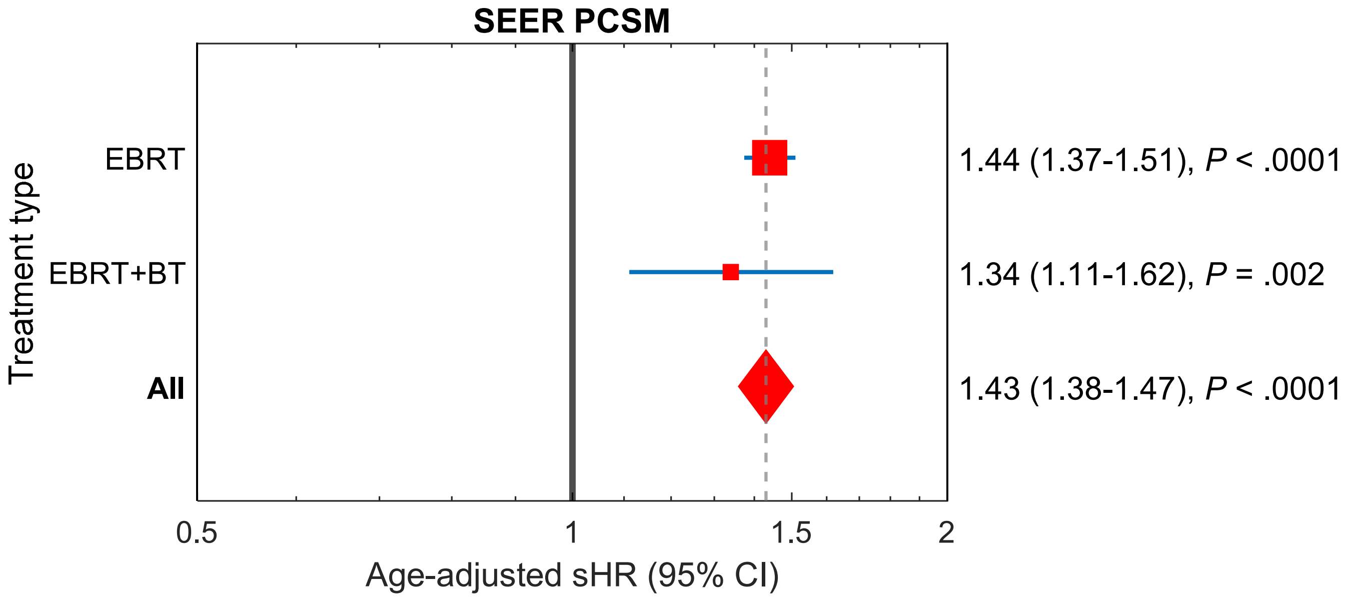

© 2021 Xiang M et al. *JAMA Network Open.*

B)

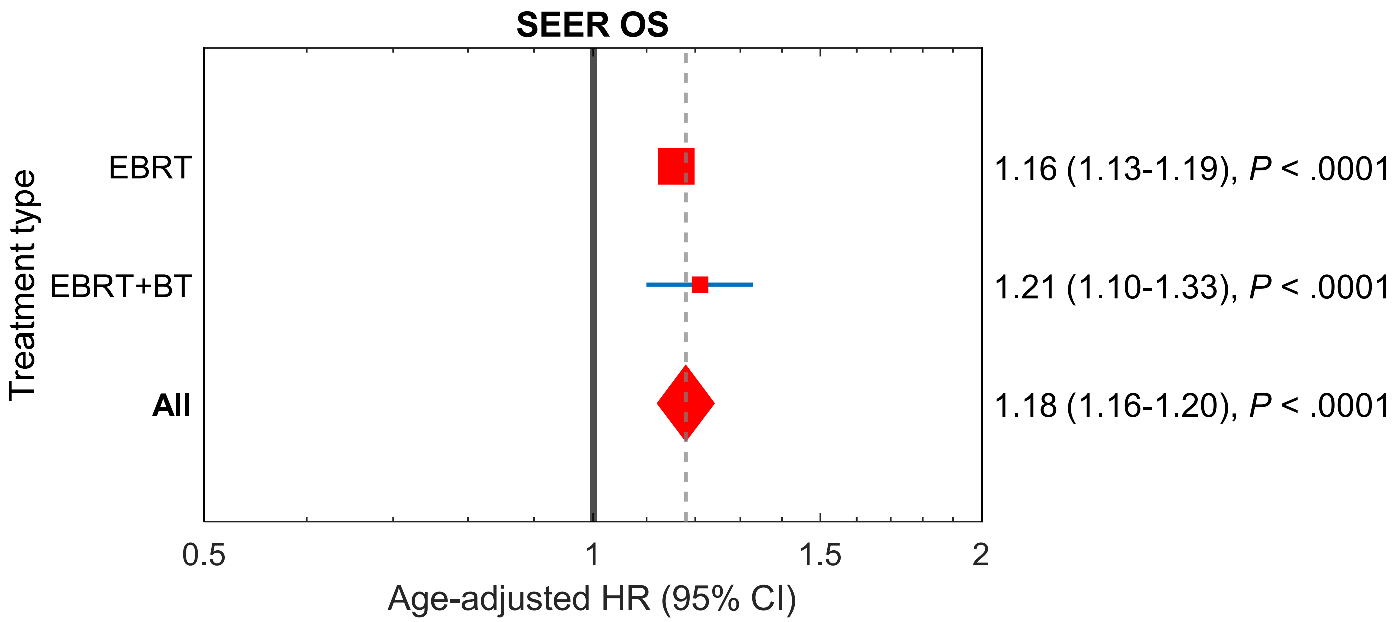

© 2021 Xiang M et al. *JAMA Network Open.*

C)

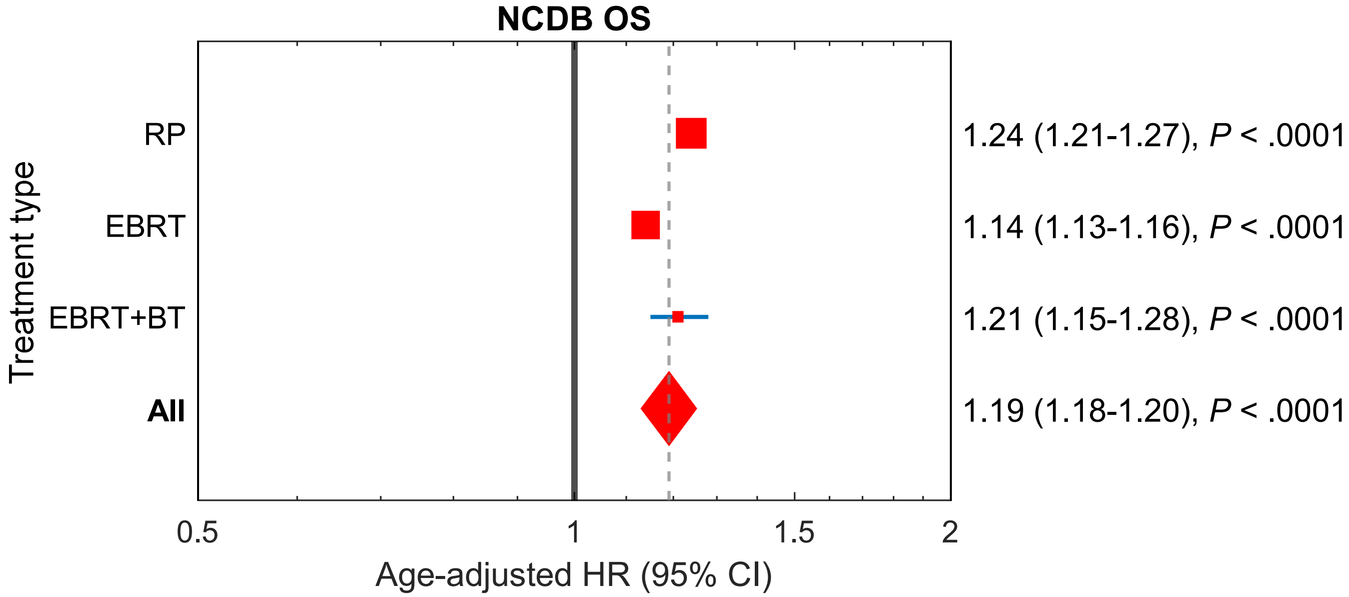

© 2021 Xiang M et al. *JAMA Network Open.*

eFigure 5. Distribution of PSMA Nomogram Upstage Risk According to STAR-CAP Stage Groups

Distribution of PSMA nomogram upstage risk according to STAR-CAP stage group in the A) multi-institutional cohort, B) SEER cohort, and C) NCDB cohort. Boxes denote medians and 25th - 75th percentiles; whiskers span the 2.5th - 97.5th percentiles. Jonckheere-Terpstra test for trend: $P < .0001$ in all cohorts.

A)

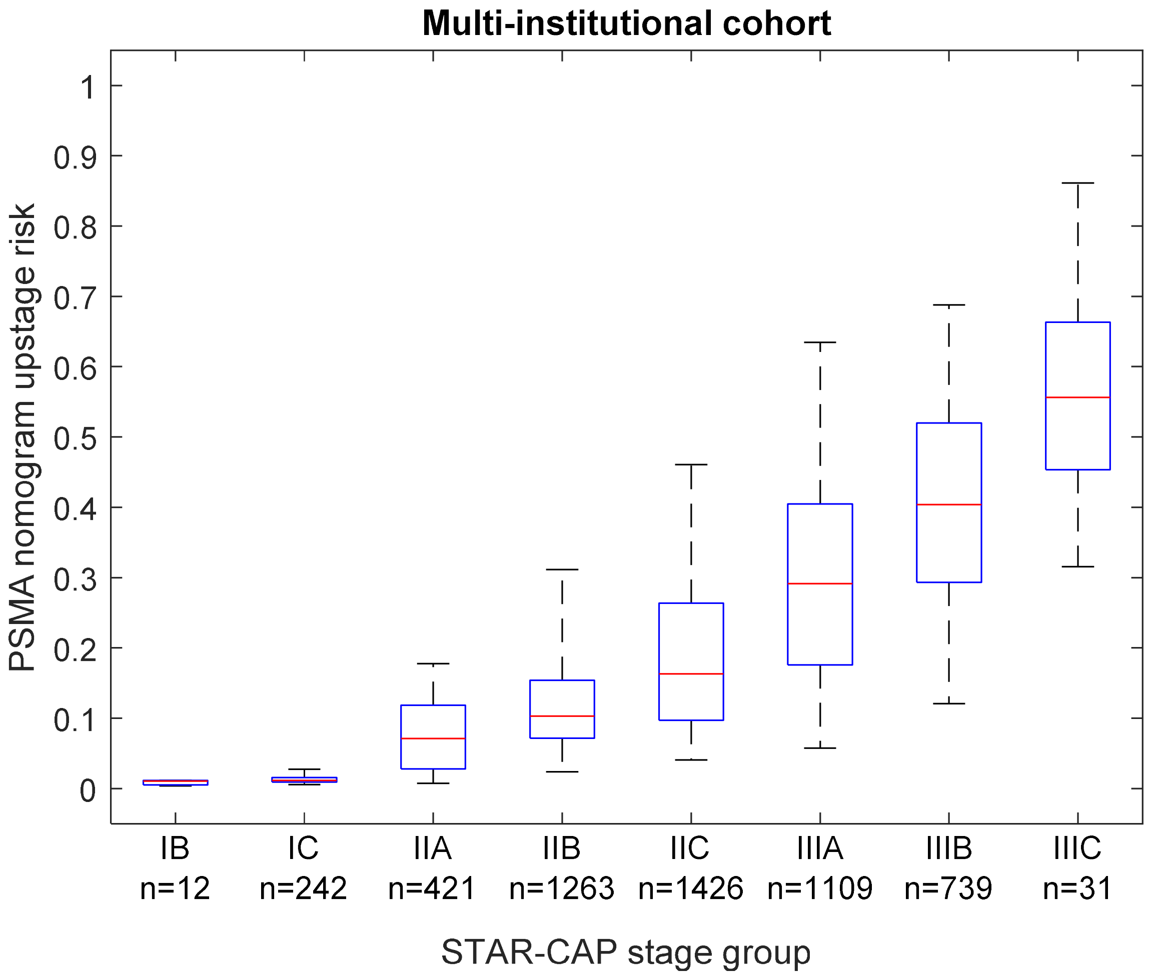

© 2021 Xiang M et al. *JAMA Network Open.*

B)

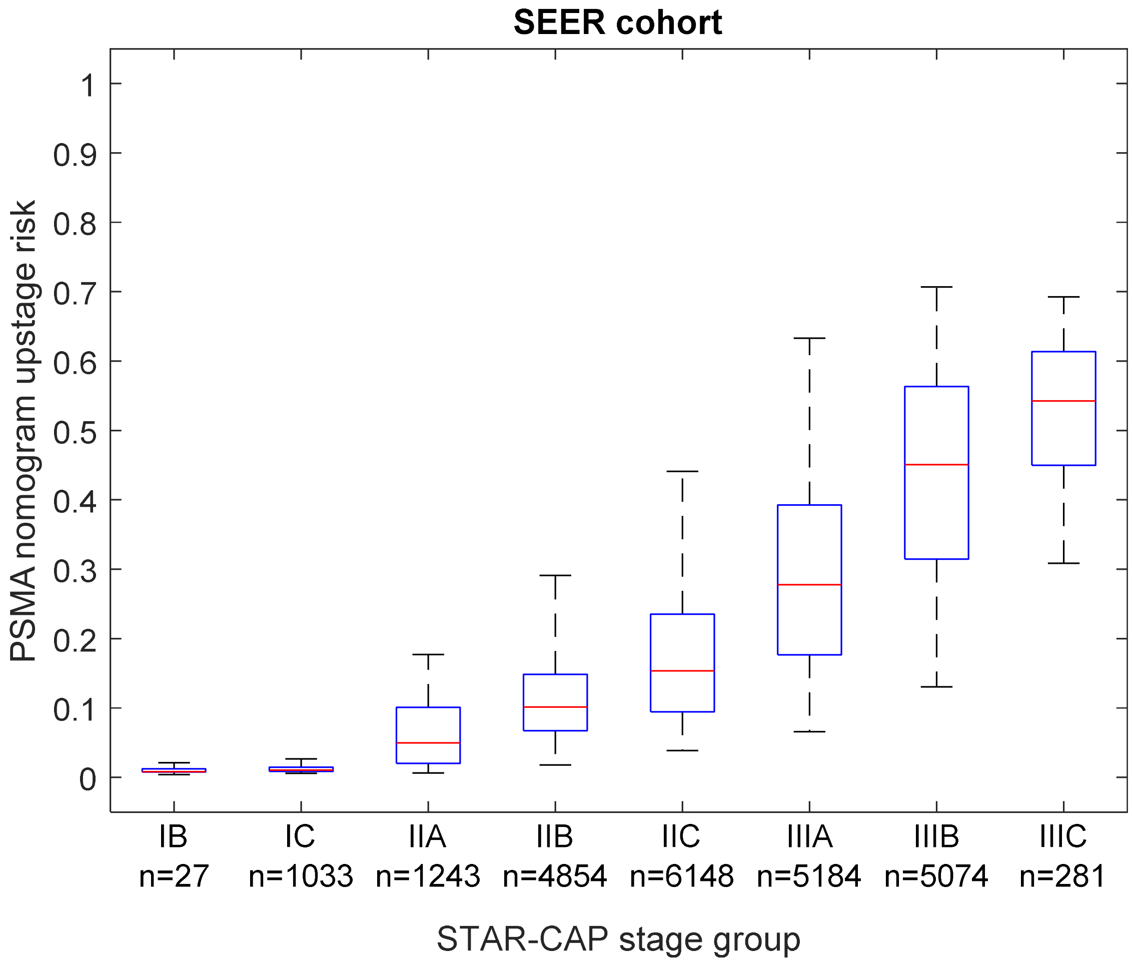

© 2021 Xiang M et al. *JAMA Network Open.*

C)

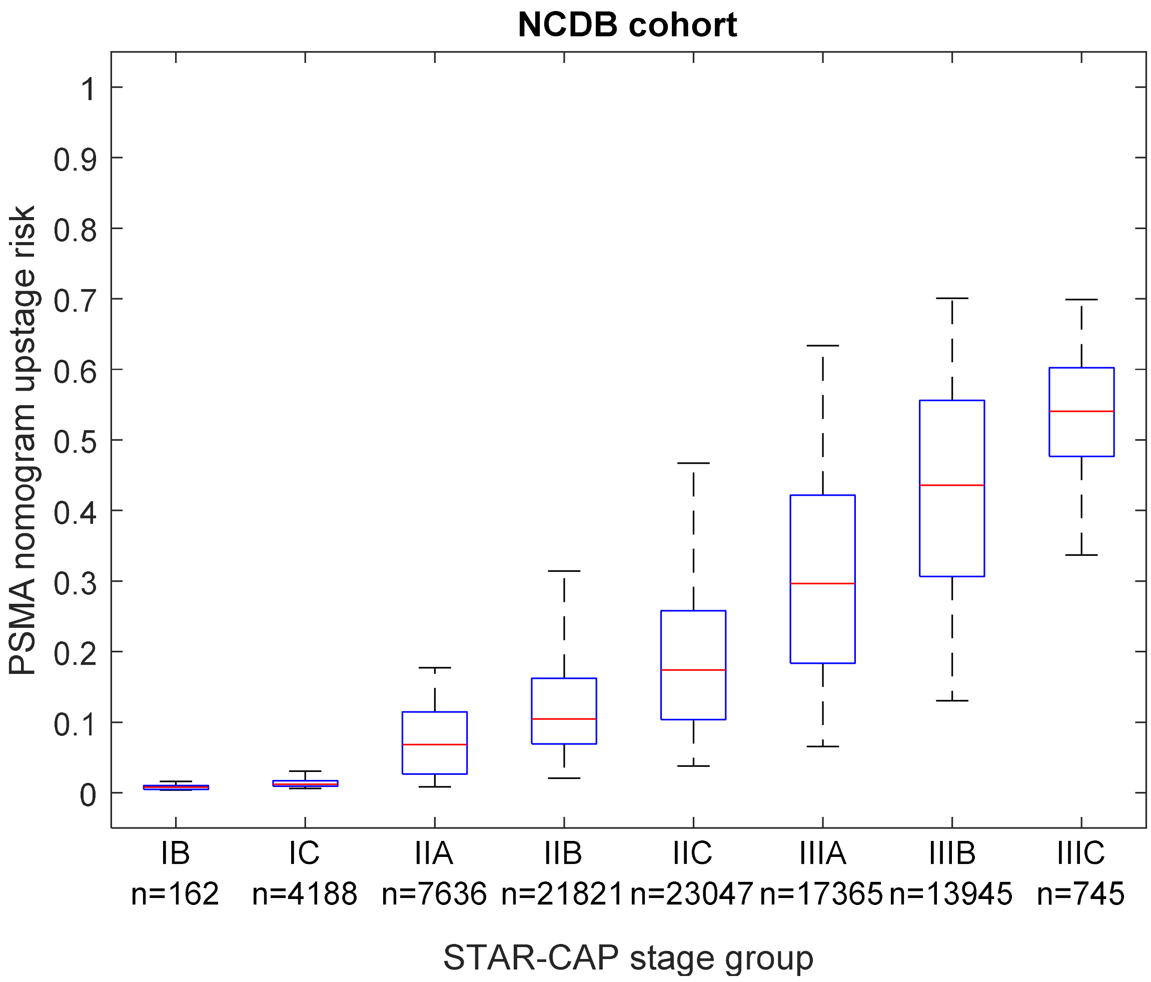

© 2021 Xiang M et al. *JAMA Network Open.*

eFigure 6. Index of Prediction Accuracy (IPA) for the PSMA Nomogram and Other Models (STAR-CAP, CAPRA, and MSKCC Nomogram) in the Multi-institutional Cohort

Endpoints are A) biochemical recurrence (BCR), B) distant metastasis (DM), C) prostate cancer-specific mortality (PCSM), D) overall survival (OS).
A)

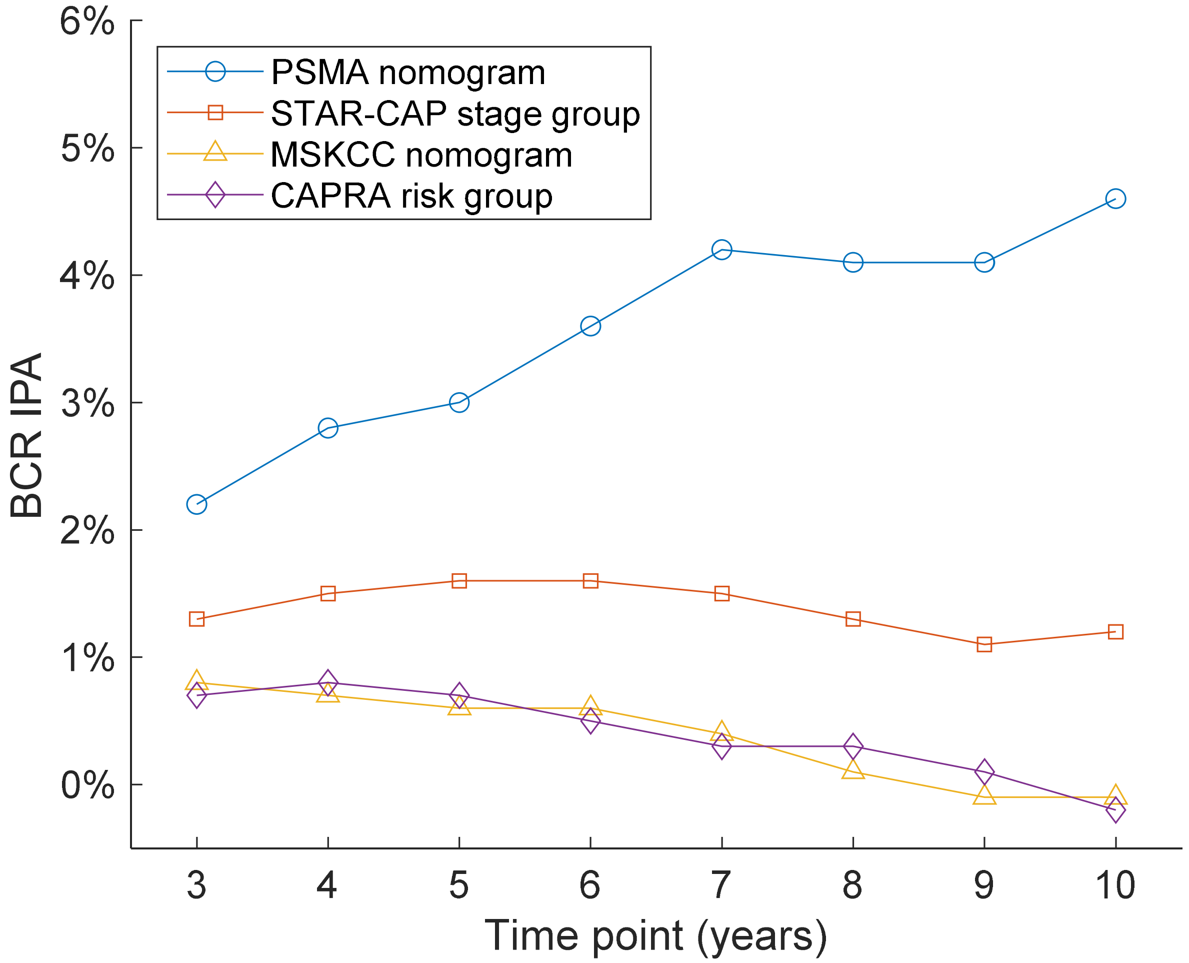

© 2021 Xiang M et al. *JAMA Network Open.*

B)

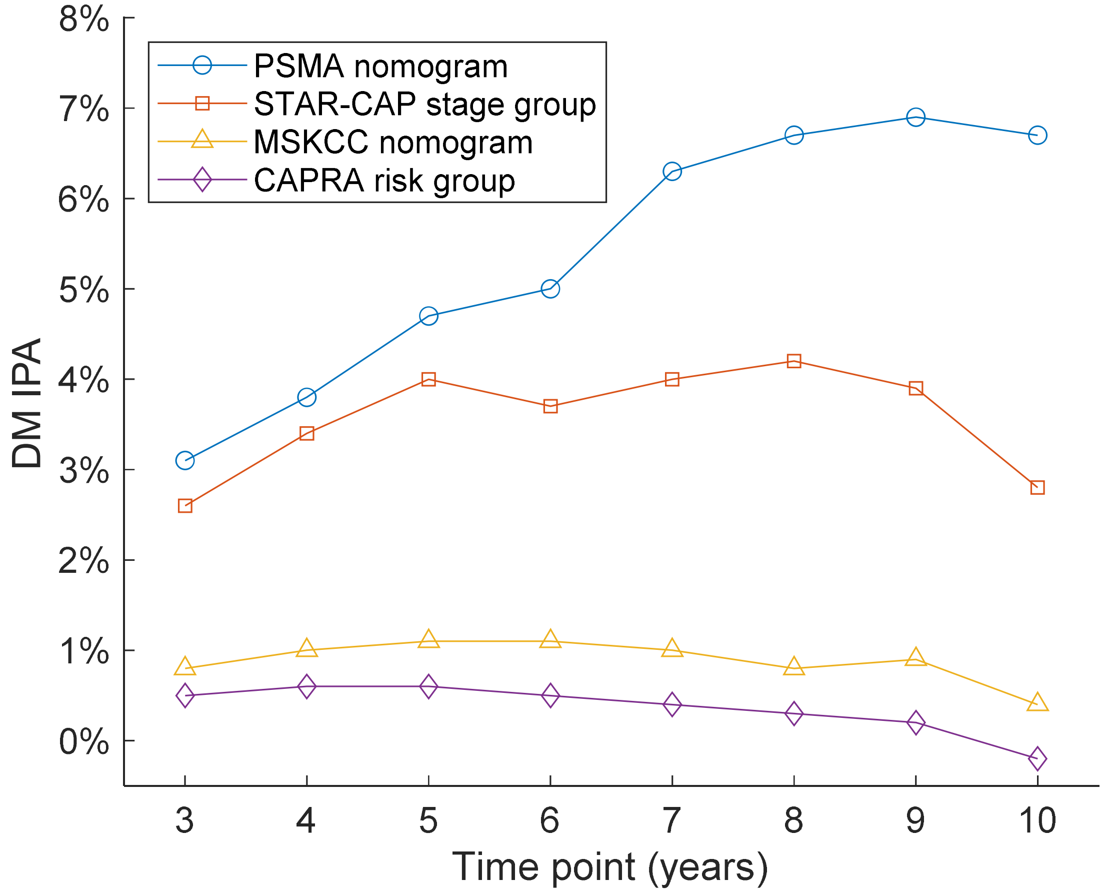

© 2021 Xiang M et al. *JAMA Network Open.*

C)

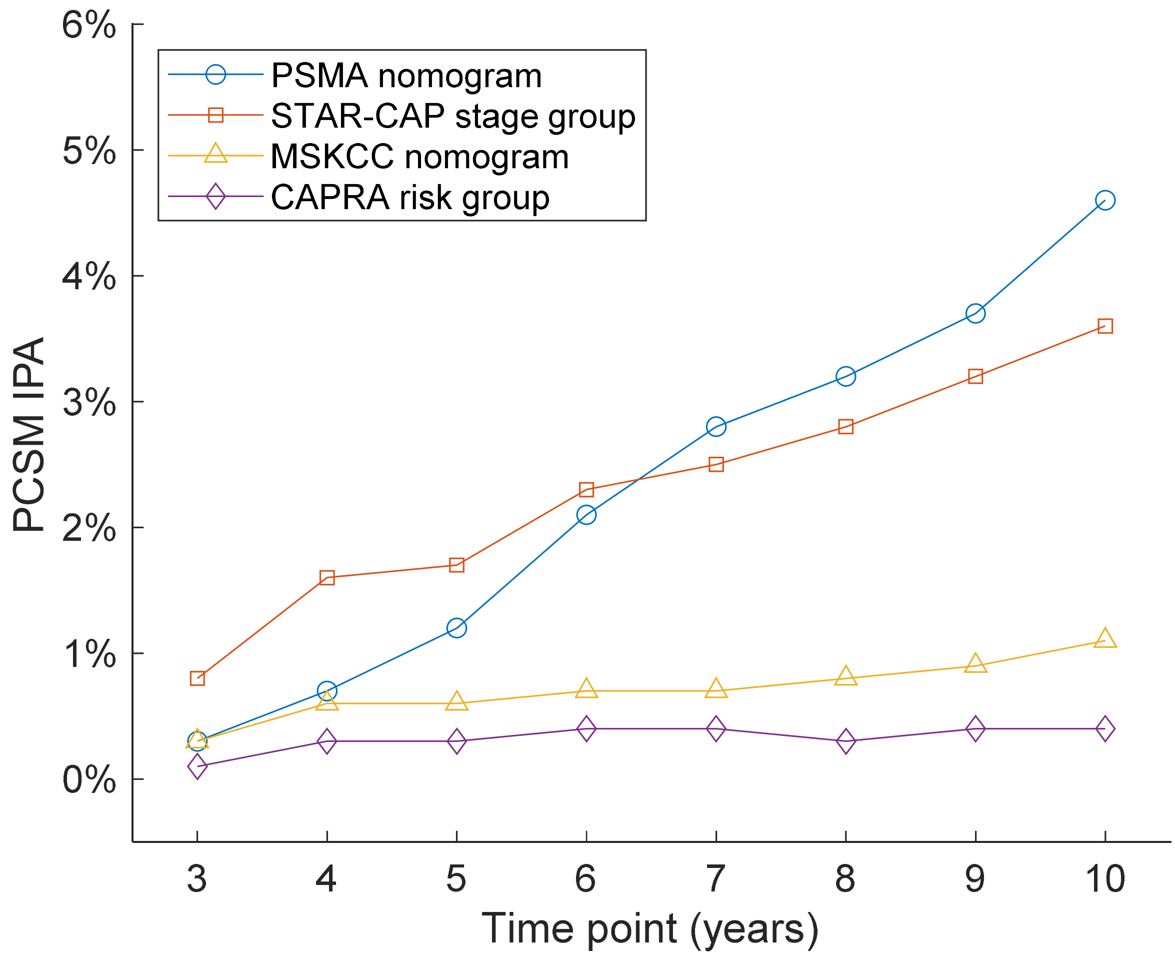

© 2021 Xiang M et al. *JAMA Network Open.*

D)

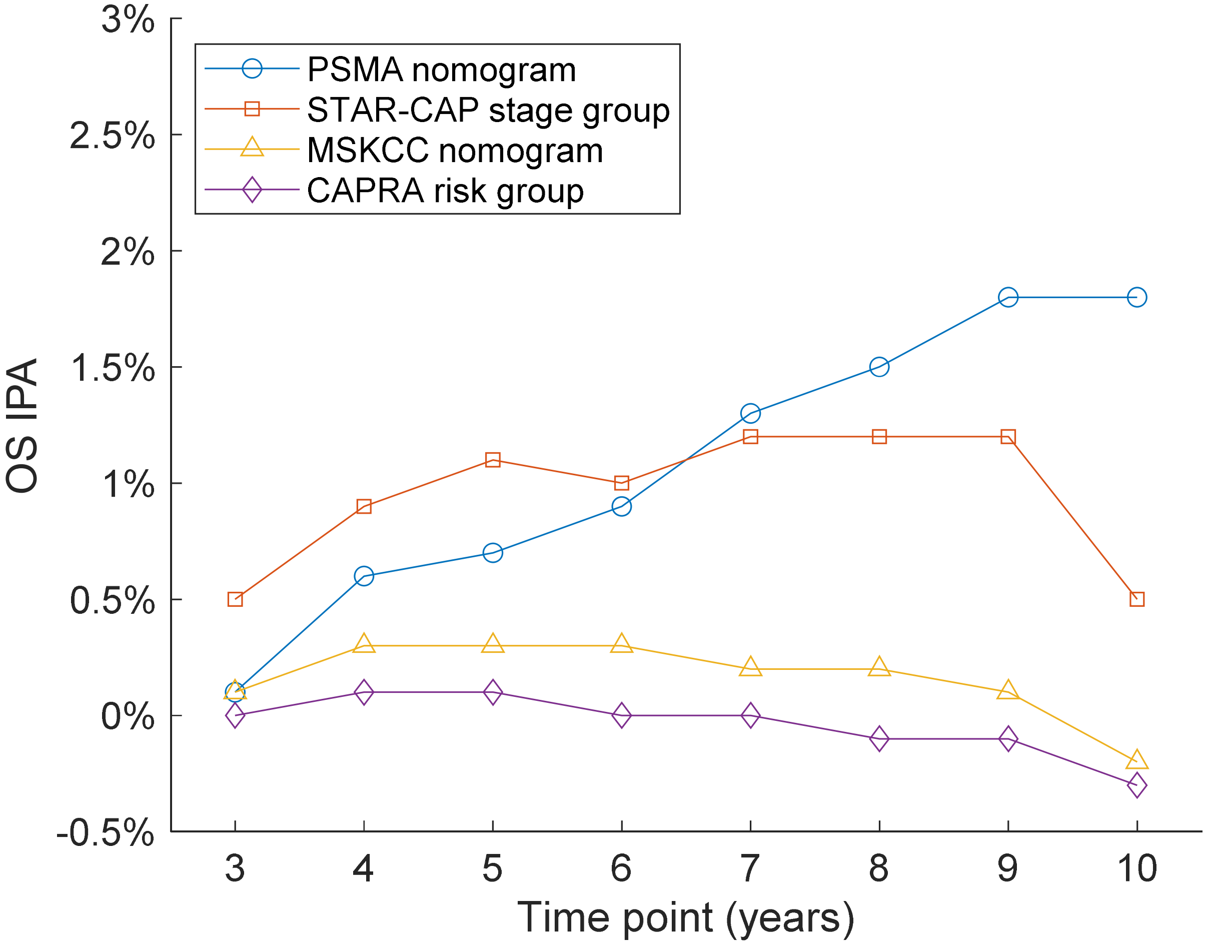

© 2021 Xiang M et al. *JAMA Network Open.*

eFigure 7. Index of Prediction Accuracy (IPA) for the PSMA Nomogram and Other Models in the Registry-Based (SEER and NCDB) Cohorts

Endpoints are A) prostate cancer-specific mortality (PCSM) in the SEER database, B) overall survival (OS) in the SEER database, and C) OS in the NCDB.
A)

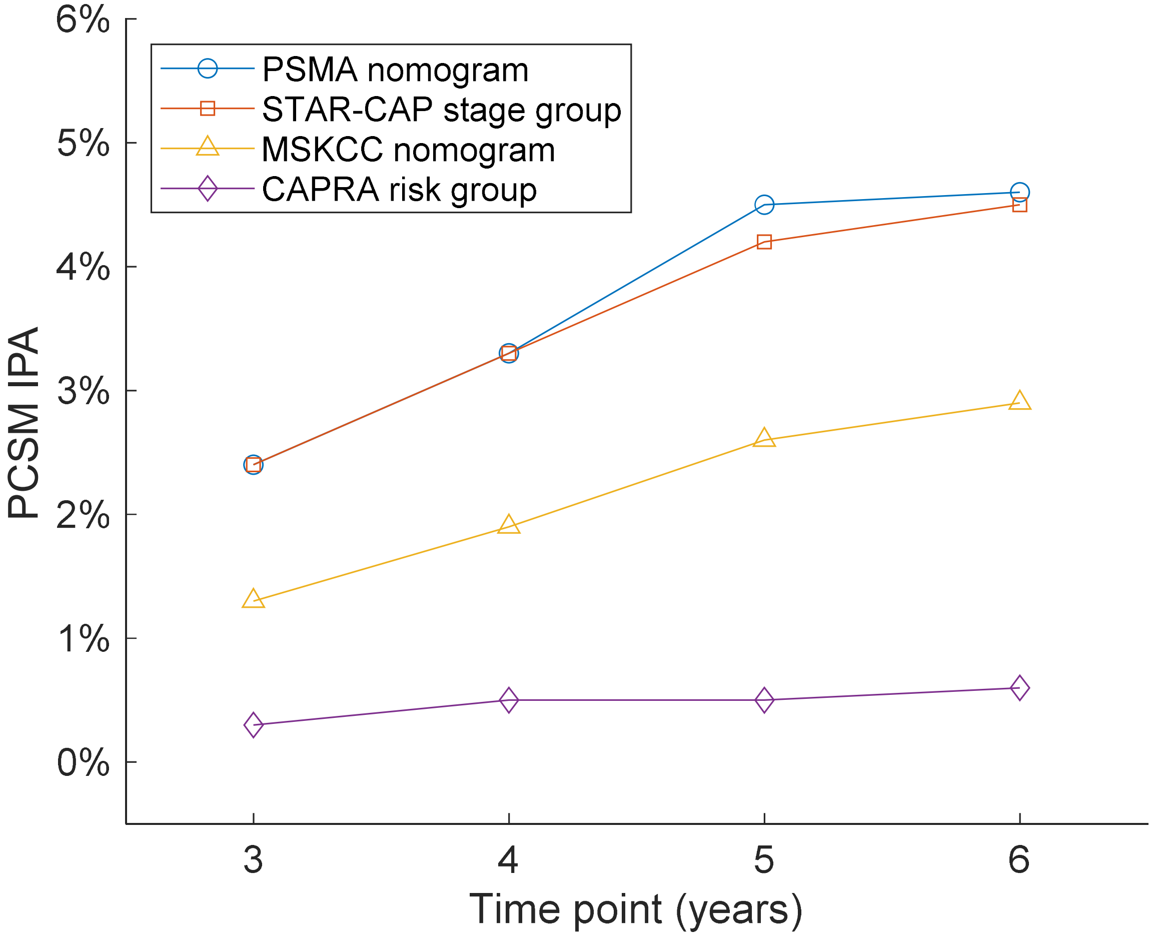

© 2021 Xiang M et al. *JAMA Network Open.*

B)

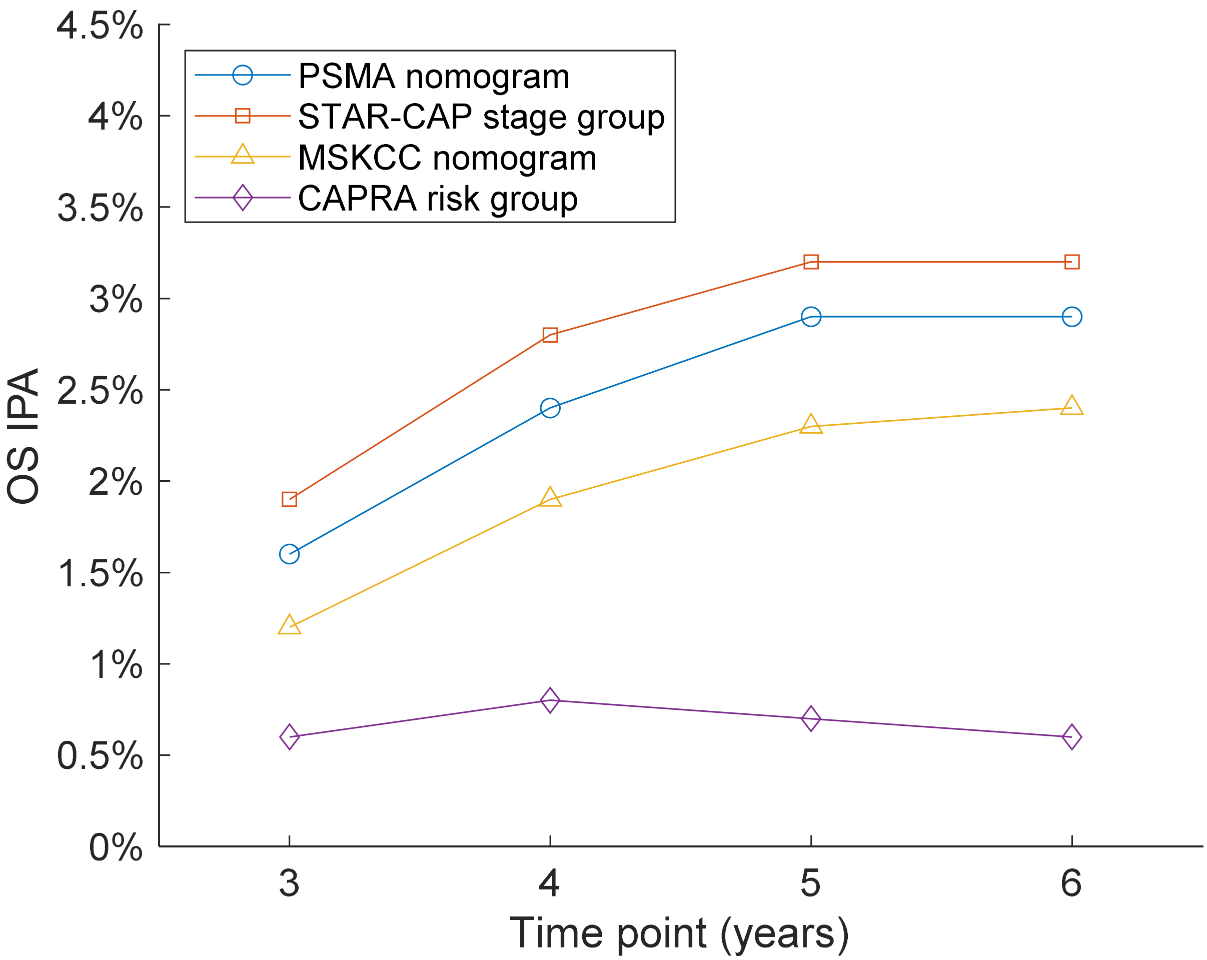

© 2021 Xiang M et al. *JAMA Network Open.*

C)

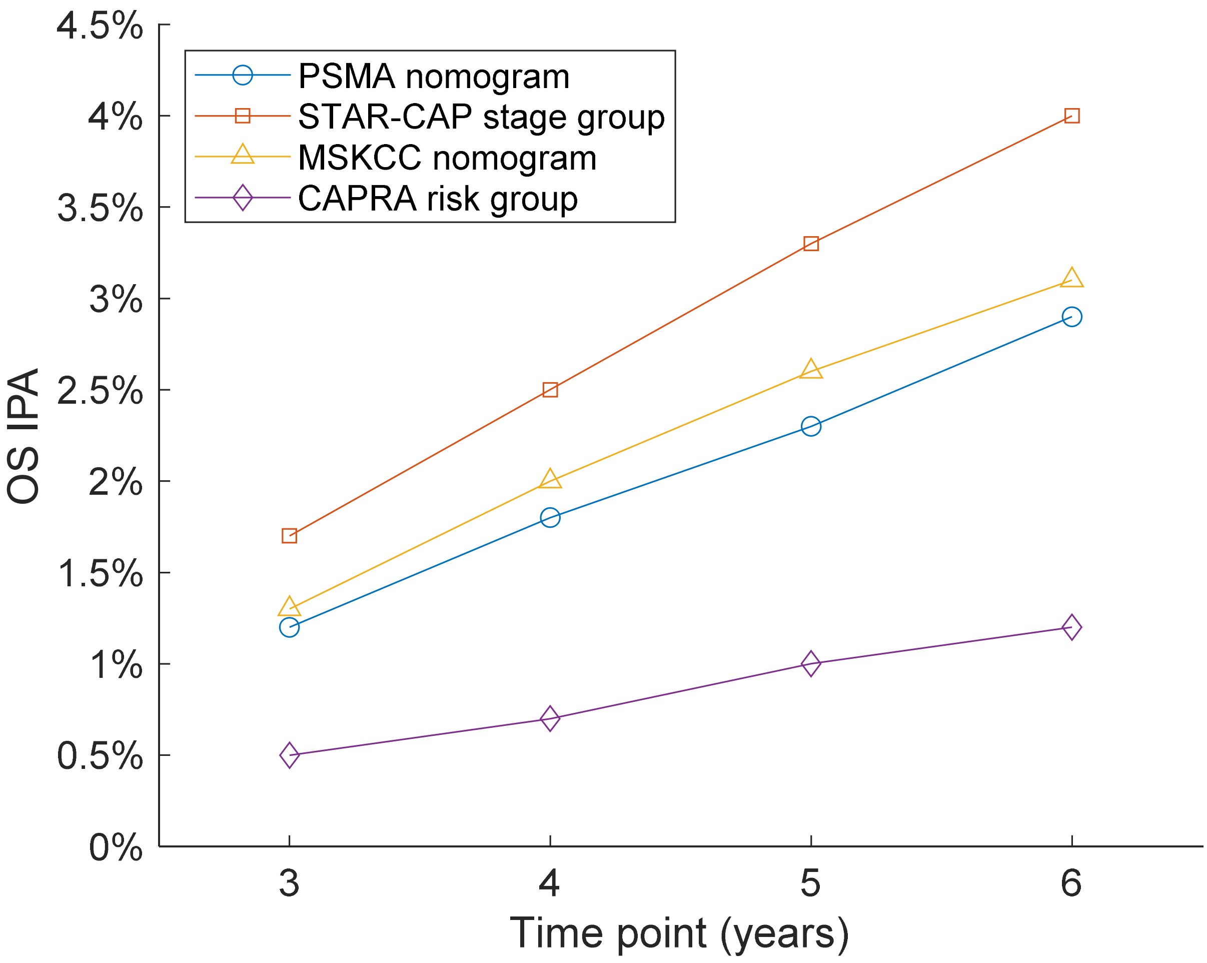

© 2021 Xiang M et al. *JAMA Network Open.*

eFigure 8. Performance of the PSMA Nomogram and Other Models in the Multi-institutional Cohort for Patients Treated With Radical Prostatectomy

Performance assessed by the concordance indices (C-indices). Endpoints are A) biochemical recurrence (BCR), B) distant metastasis (DM), C) prostate cancer-specific mortality (PCSM), D) overall survival (OS). Error bars represent 95% confidence intervals (CI). P-values are for comparisons versus the PSMA nomogram at the 5- and 8-year time points.

A)

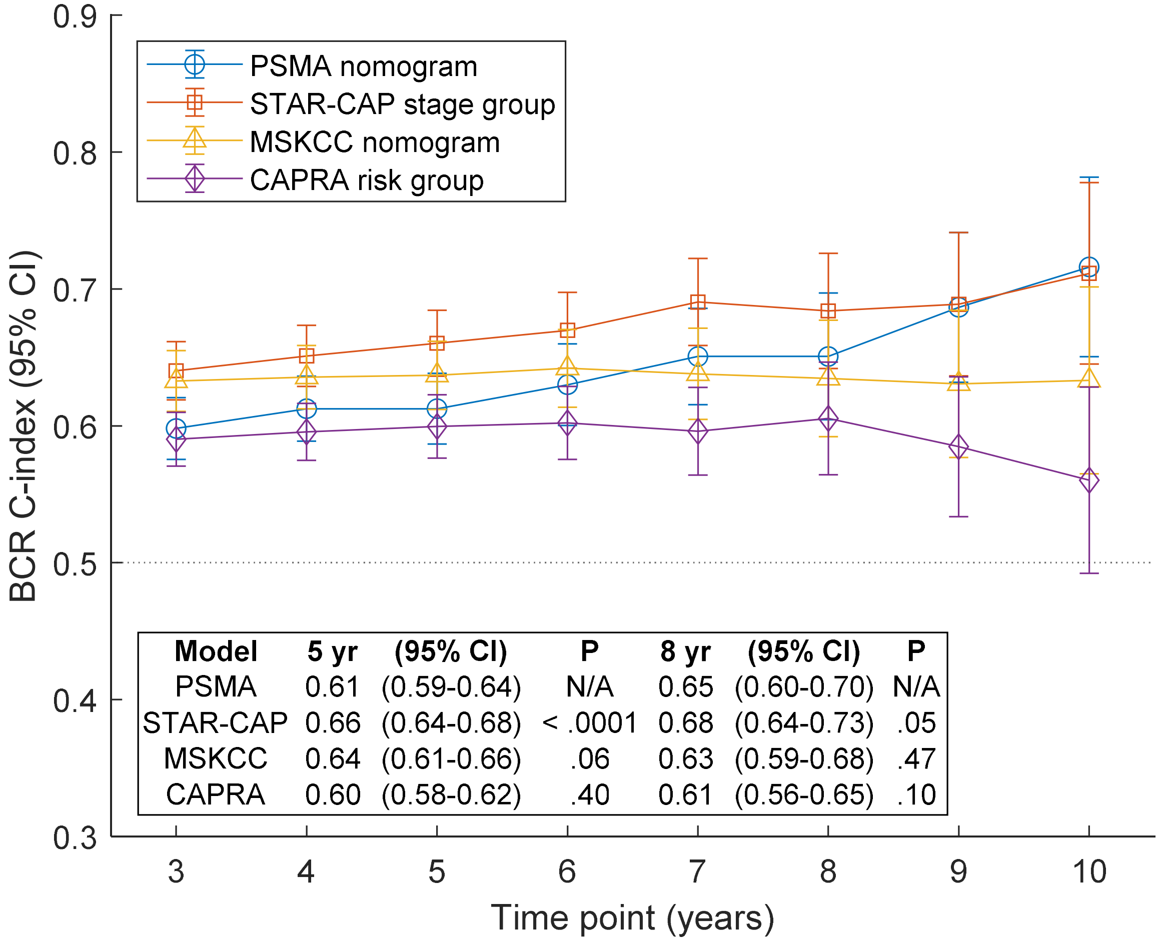

**Model**	**5 yr**	**(95% CI)**	**P**	**8 yr**	**(95% CI)**	**P**
PSMA	0.61	(0.59-0.64)	N/A	0.65	(0.60-0.70)	N/A
STAR-CAP	0.66	(0.64-0.68)	< .0001	0.68	(0.64-0.73)	.05
MSKCC	0.64	(0.61-0.66)	.06	0.63	(0.59-0.68)	.47
CAPRA	0.60	(0.58-0.62)	.40	0.61	(0.56-0.65)	.10

© 2021 Xiang M et al. *JAMA Network Open.*

B)

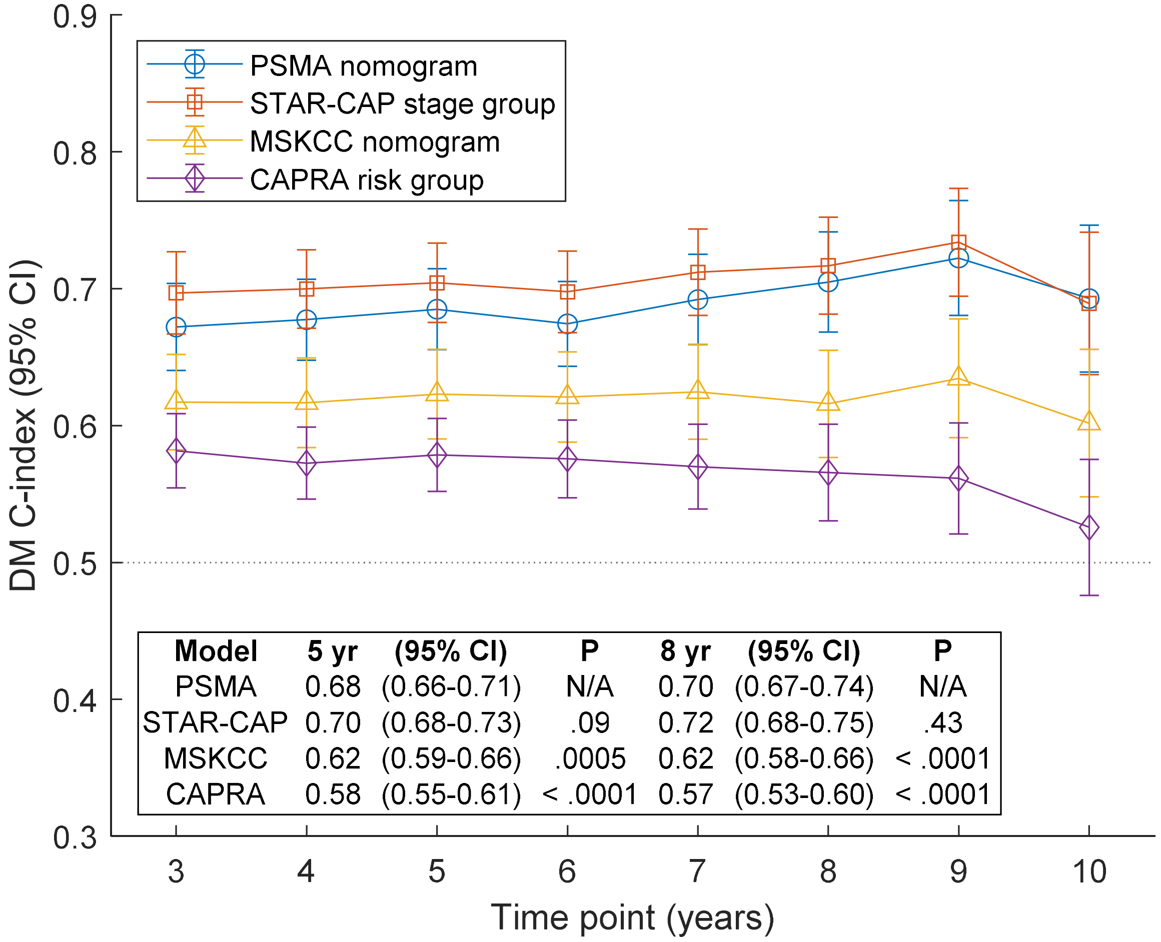

© 2021 Xiang M et al. *JAMA Network Open*.

C)

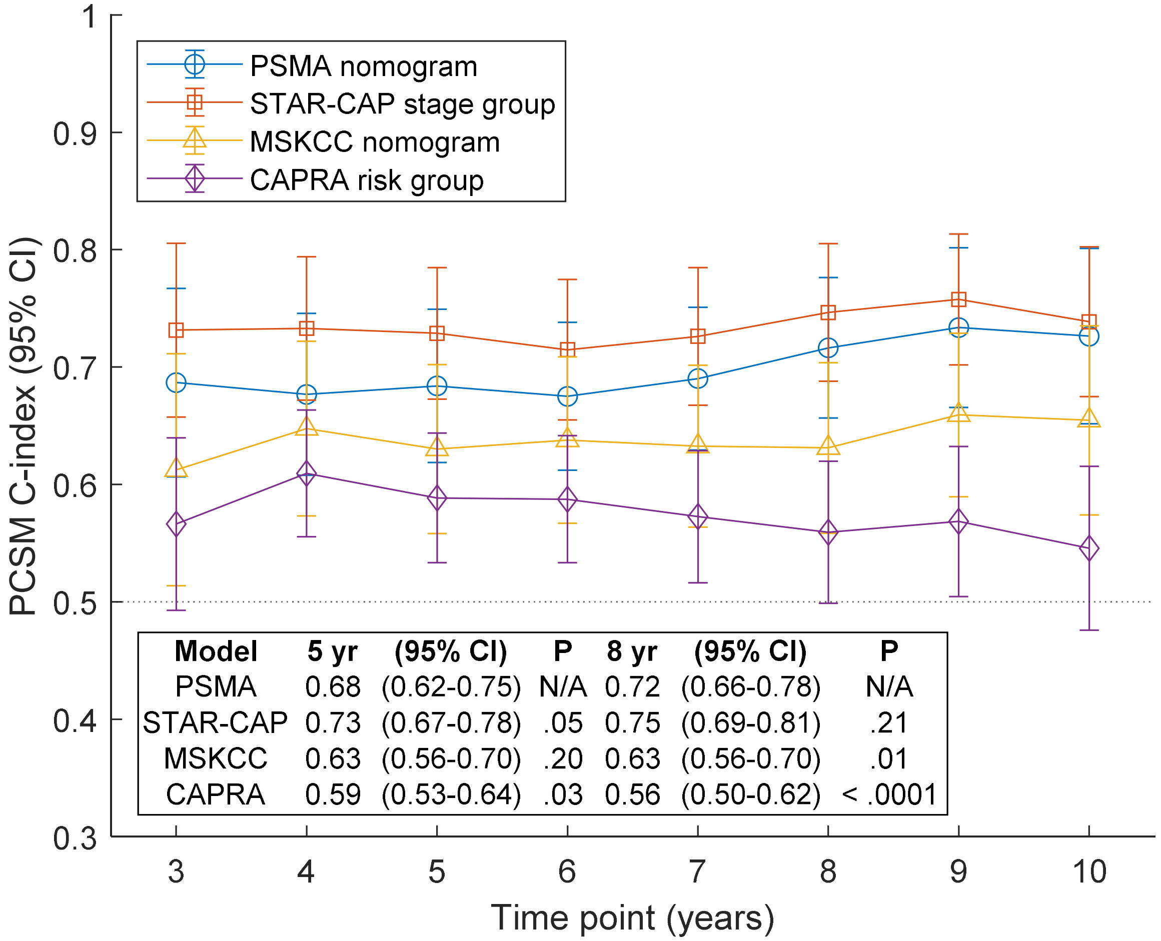

Model	5 yr	(95% CI)	P	8 yr	(95% CI)	P
PSMA	0.68	(0.62-0.75)	N/A	0.72	(0.66-0.78)	N/A
STAR-CAP	0.73	(0.67-0.78)	.05	0.75	(0.69-0.81)	.21
MSKCC	0.63	(0.56-0.70)	.20	0.63	(0.56-0.70)	.01
CAPRA	0.59	(0.53-0.64)	.03	0.56	(0.50-0.62)	< .0001

© 2021 Xiang M et al. *JAMA Network Open.*

D)

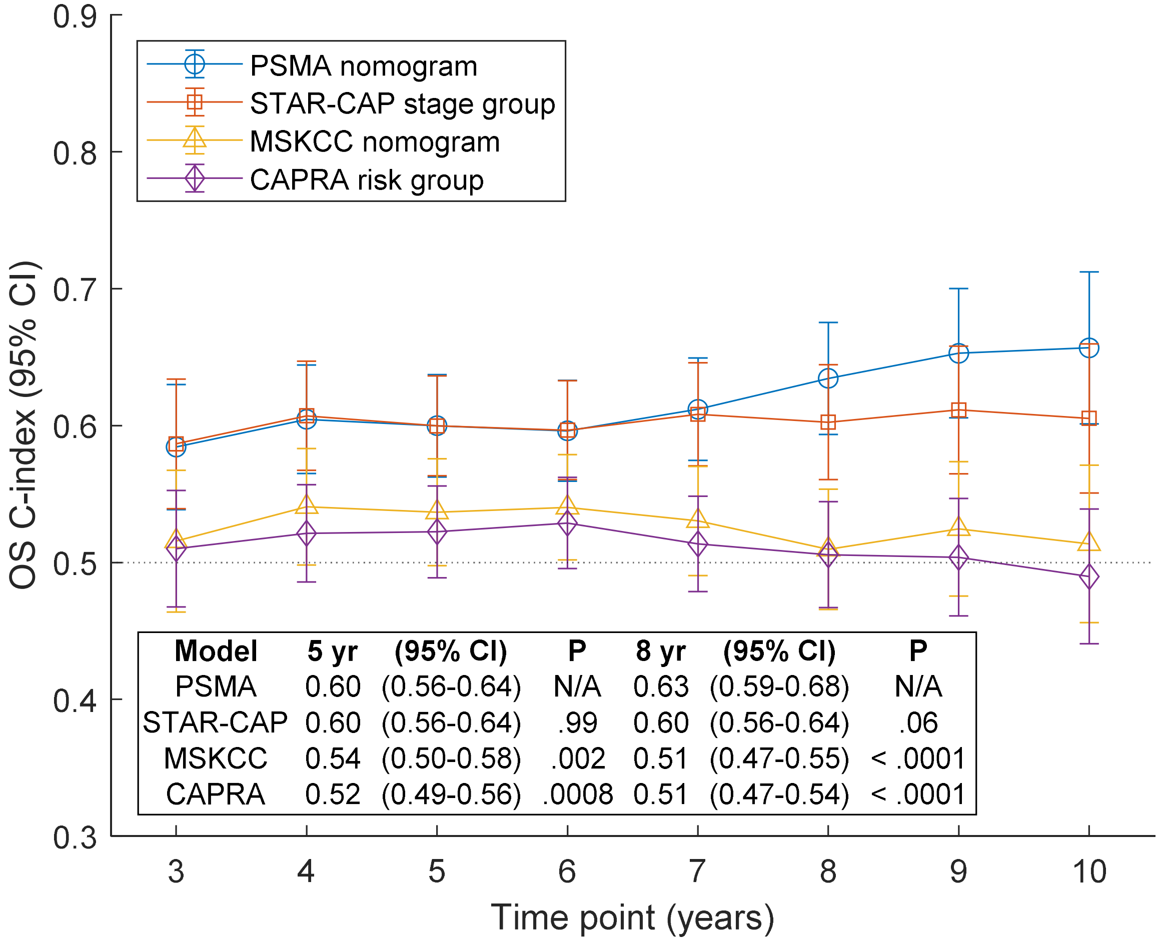

© 2021 Xiang M et al. *JAMA Network Open.*

eFigure 9. Performance of the PSMA Nomogram and Other Models in the Multi-institutional Cohort for Patients Treated With External Beam Radiation

Performance assessed by the concordance indices (C-indices). Endpoints are A) biochemical recurrence (BCR), B) distant metastasis (DM), C) prostate cancer-specific mortality (PCSM), D) overall survival (OS). Error bars represent 95% confidence intervals (CI). P-values are for comparisons versus the PSMA nomogram at the 5- and 8-year time points.

A)

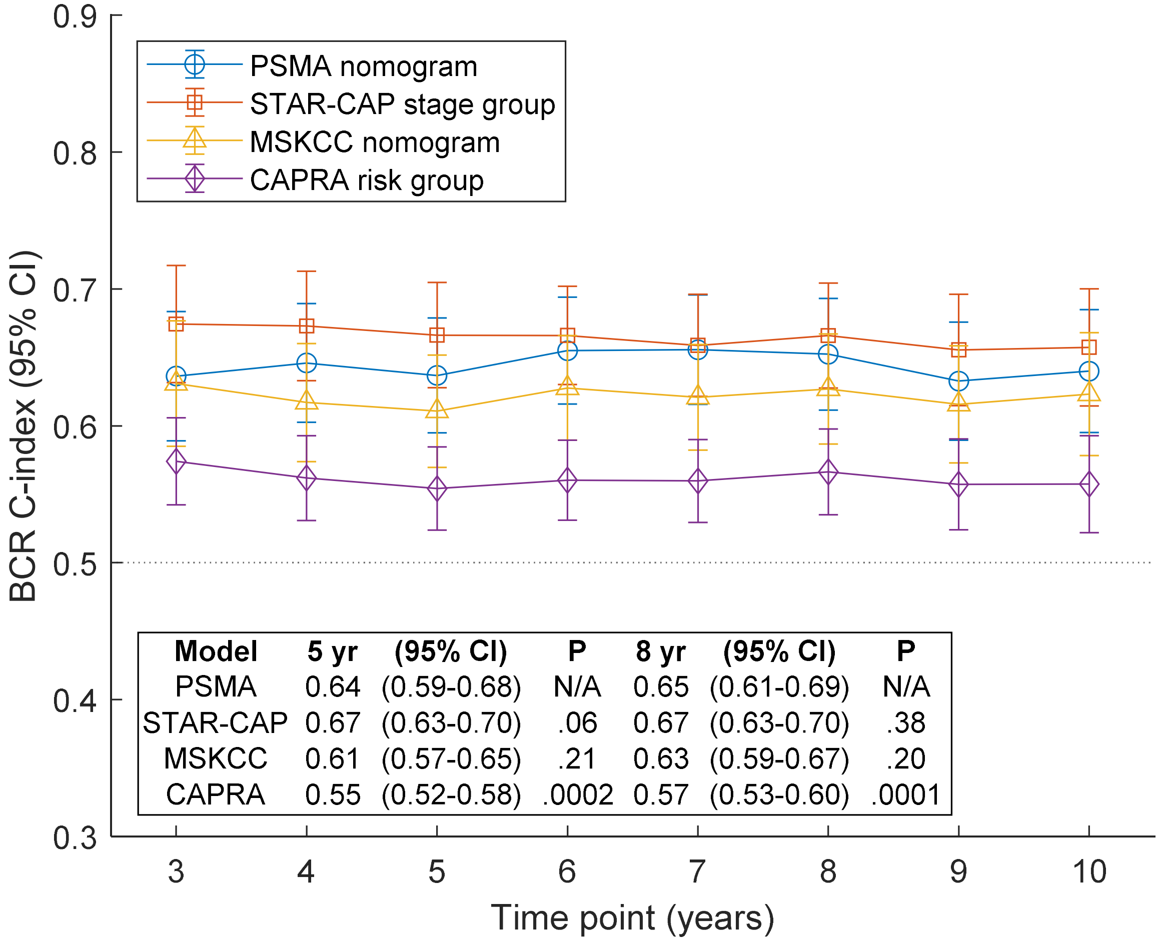

Model	5 yr	(95% CI)	P	8 yr	(95% CI)	P
PSMA	0.64	(0.59-0.68)	N/A	0.65	(0.61-0.69)	N/A
STAR-CAP	0.67	(0.63-0.70)	.06	0.67	(0.63-0.70)	.38
MSKCC	0.61	(0.57-0.65)	.21	0.63	(0.59-0.67)	.20
CAPRA	0.55	(0.52-0.58)	.0002	0.57	(0.53-0.60)	.0001

© 2021 Xiang M et al. *JAMA Network Open.*

B)

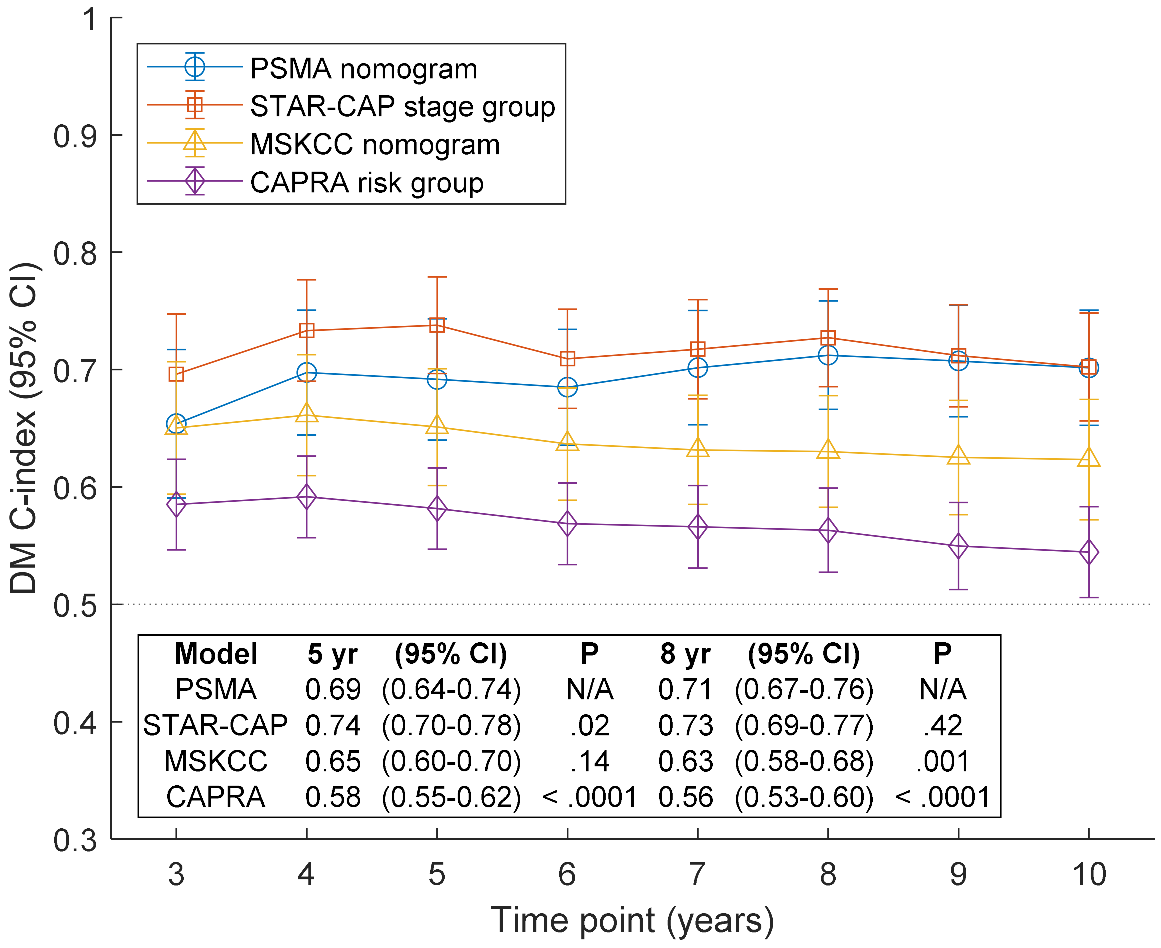

© 2021 Xiang M et al. *JAMA Network Open.*

C)

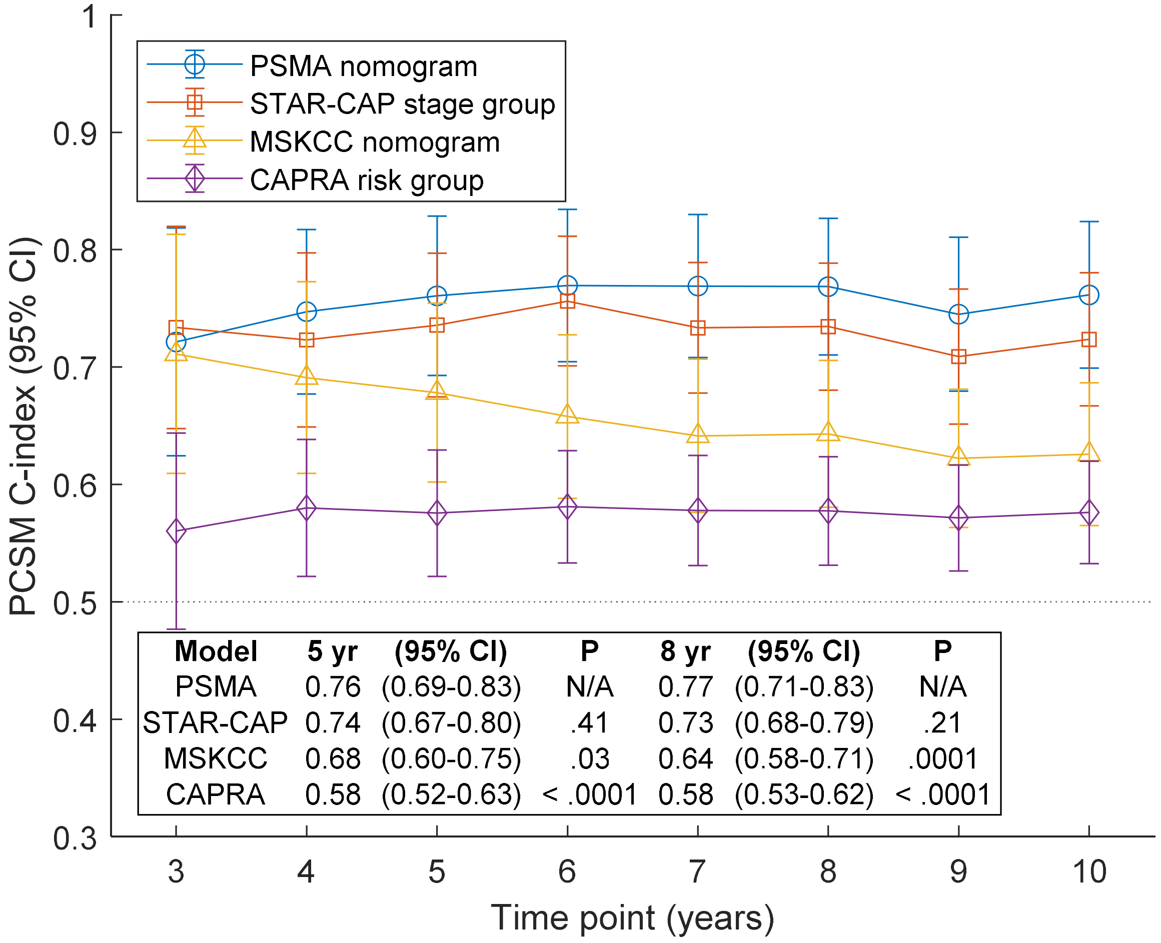

Model	5 yr	(95% CI)	P	8 yr	(95% CI)	P
PSMA	0.76	(0.69-0.83)	N/A	0.77	(0.71-0.83)	N/A
STAR-CAP	0.74	(0.67-0.80)	.41	0.73	(0.68-0.79)	.21
MSKCC	0.68	(0.60-0.75)	.03	0.64	(0.58-0.71)	.0001
CAPRA	0.58	(0.52-0.63)	< .0001	0.58	(0.53-0.62)	< .0001

© 2021 Xiang M et al. *JAMA Network Open.*

D)

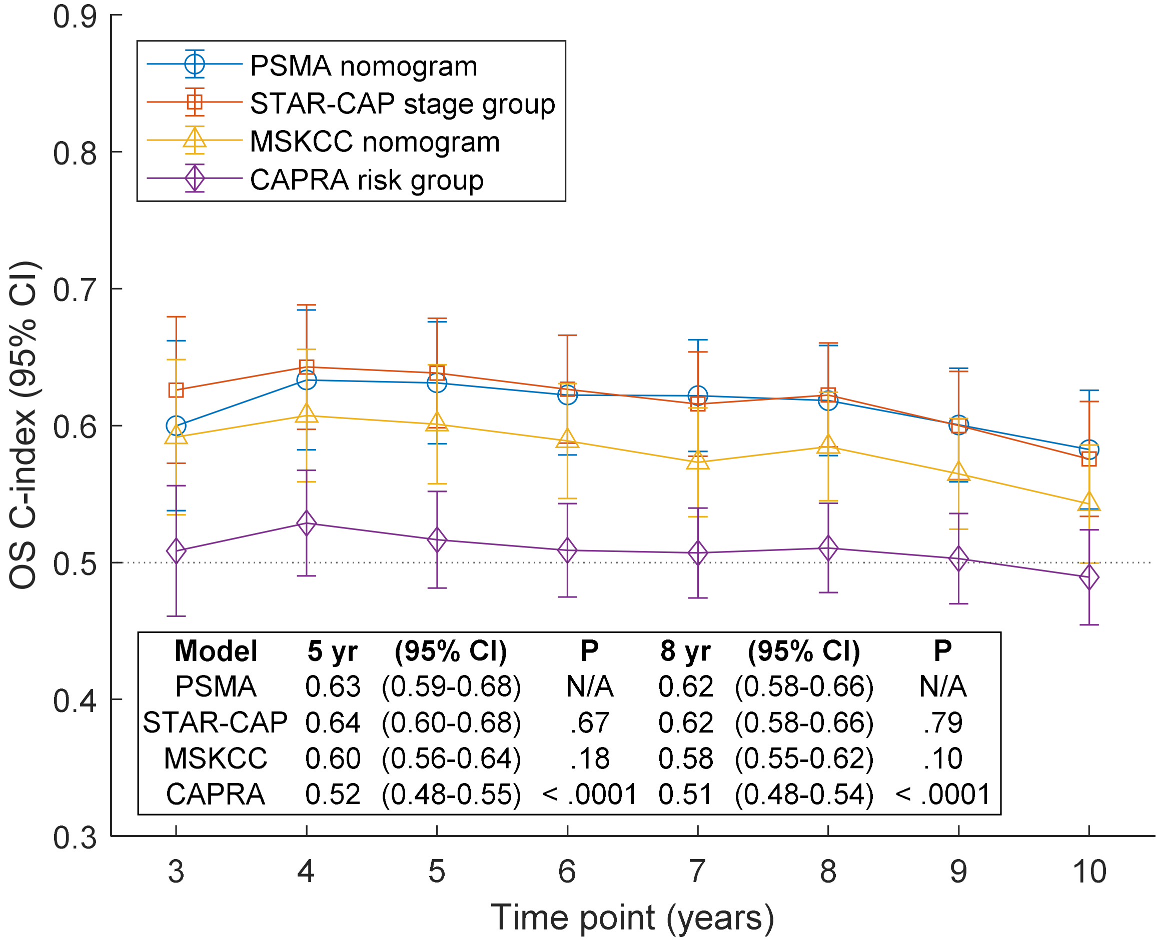

© 2021 Xiang M et al. *JAMA Network Open.*

eFigure 10. Performance of the PSMA Nomogram and Other Models in the Multi-institutional Cohort for Patients Treated With External Beam Radiation Plus Brachytherapy

Performance assessed by concordance (C-) indices. Endpoints are A) biochemical recurrence (BCR), B) distant metastasis (DM), C) prostate cancer-specific mortality (PCSM), D) overall survival (OS). Error bars represent 95% confidence intervals (CI). P-values are for comparisons versus the PSMA nomogram at the 5- and 8-year time points.

A)

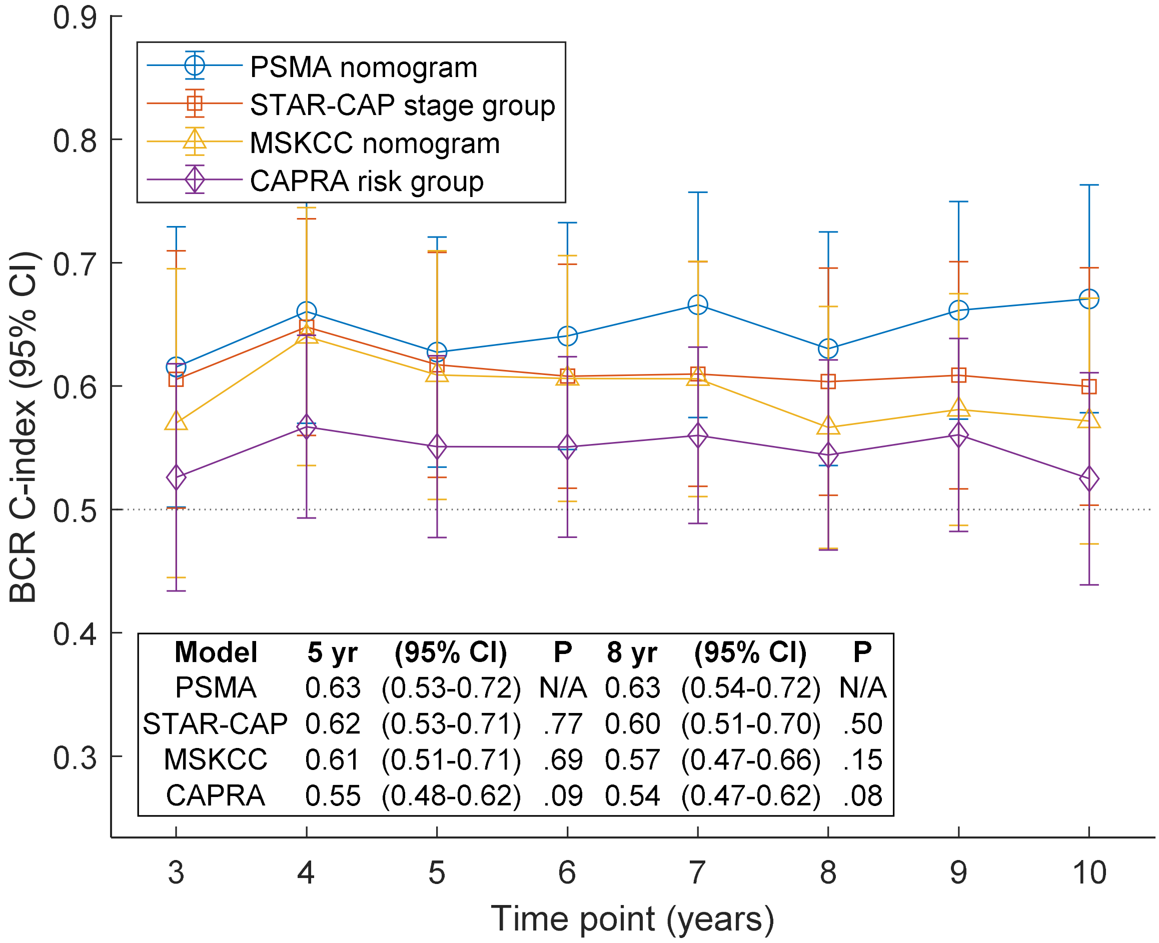

Model	5 yr	(95% CI)	P	8 yr	(95% CI)	P
PSMA	0.63	(0.53-0.72)	N/A	0.63	(0.54-0.72)	N/A
STAR-CAP	0.62	(0.53-0.71)	.77	0.60	(0.51-0.70)	.50
MSKCC	0.61	(0.51-0.71)	.69	0.57	(0.47-0.66)	.15
CAPRA	0.55	(0.48-0.62)	.09	0.54	(0.47-0.62)	.08

© 2021 Xiang M et al. *JAMA Network Open.*

B)

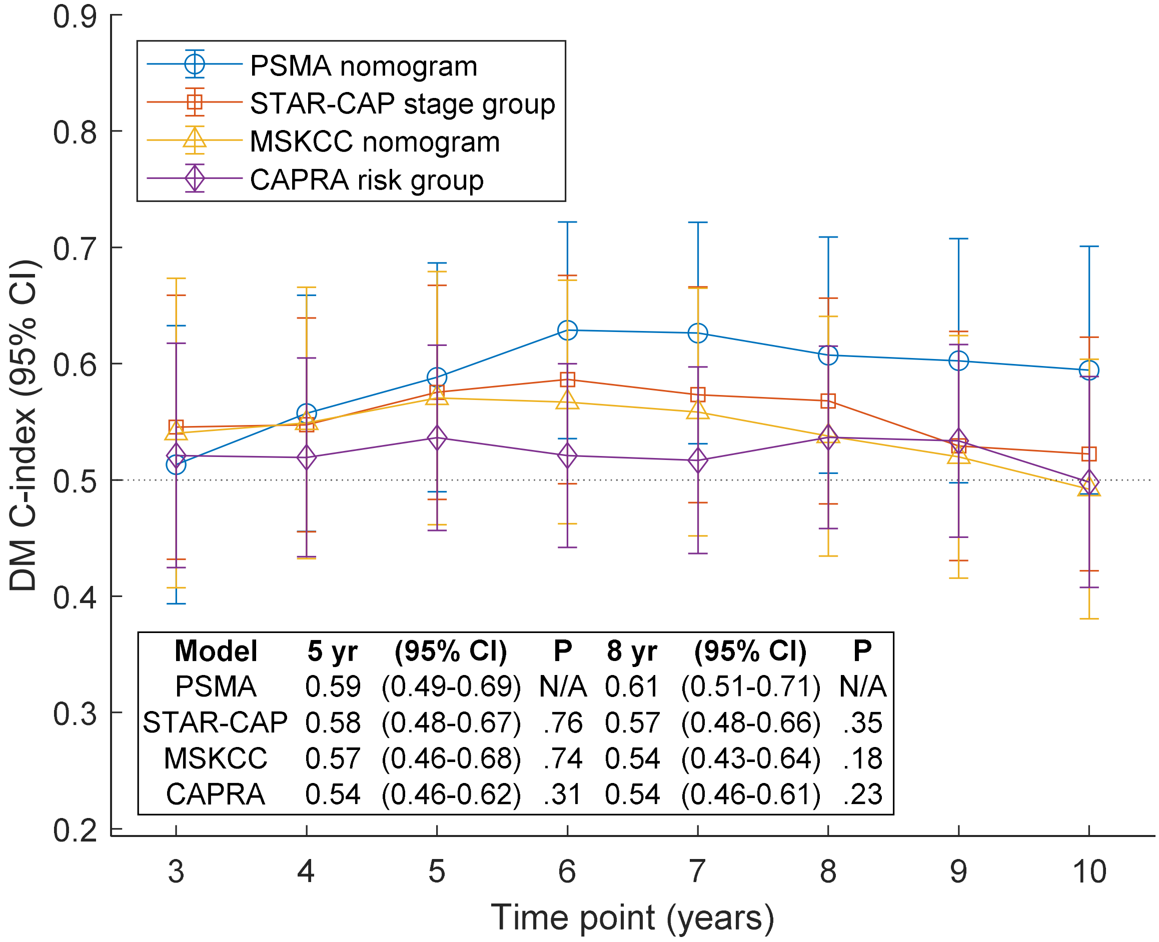

© 2021 Xiang M et al. *JAMA Network Open.*

C)

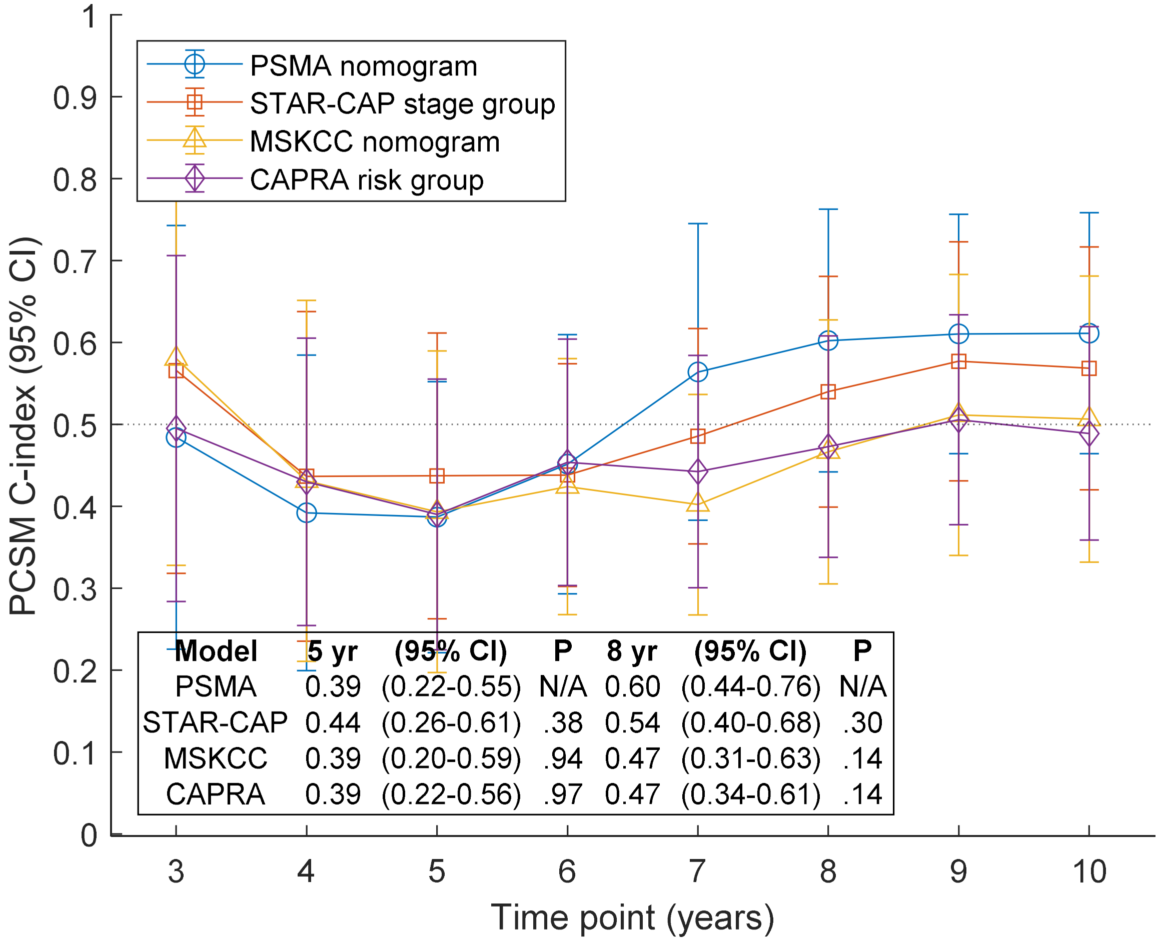

© 2021 Xiang M et al. *JAMA Network Open.*

D)

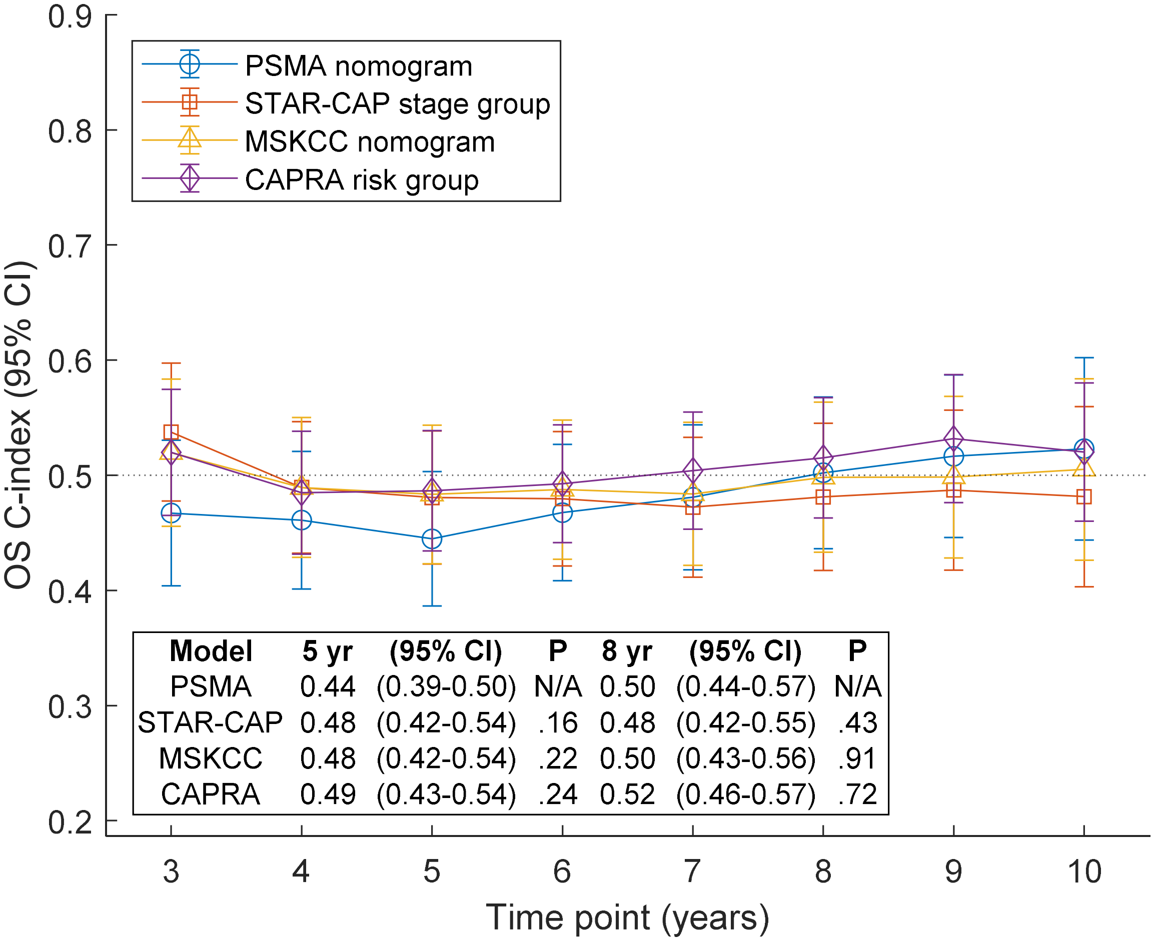

Model	5 yr	(95% CI)	P	8 yr	(95% CI)	P
PSMA	0.44	(0.39-0.50)	N/A	0.50	(0.44-0.57)	N/A
STAR-CAP	0.48	(0.42-0.54)	.16	0.48	(0.42-0.55)	.43
MSKCC	0.48	(0.42-0.54)	.22	0.50	(0.43-0.56)	.91
CAPRA	0.49	(0.43-0.54)	.24	0.52	(0.46-0.57)	.72

© 2021 Xiang M et al. *JAMA Network Open.*

eFigure 11. Performance of the PSMA Nomogram and Other Models in the SEER Cohort, Further Stratified by Type of Treatment

Performance assessed by the concordance (C-) indices for A) prostate cancer-specific mortality (PCSM) in patients treated with external beam radiation (EBRT), B) overall survival (OS) in patients treated with EBRT, C) PCSM in patients treated with EBRT + brachytherapy (BT), D) OS in patients treated with EBRT+BT. P-values are for comparisons versus the PSMA nomogram at the 3- and 5-year time points.

A)

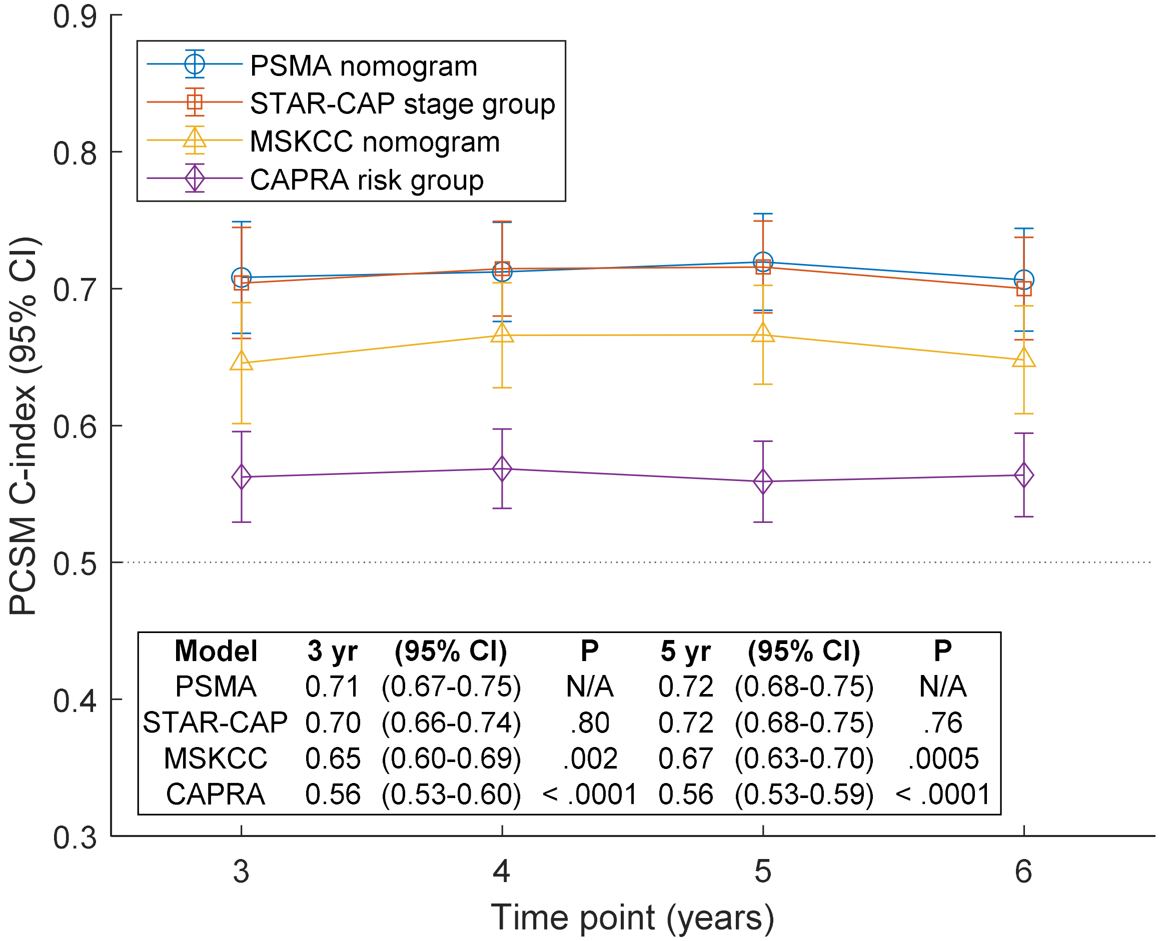

© 2021 Xiang M et al. *JAMA Network Open.*

B)

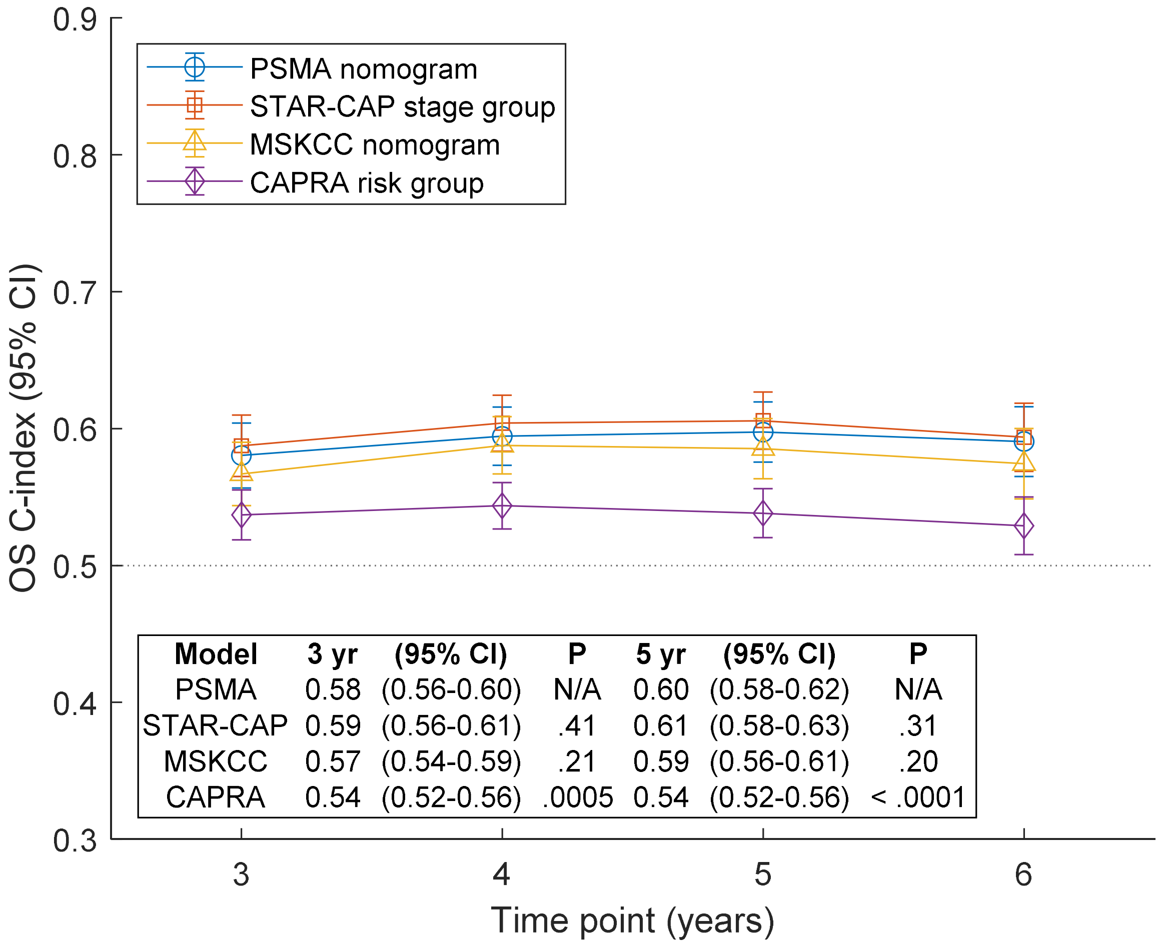

© 2021 Xiang M et al. *JAMA Network Open.*

C)

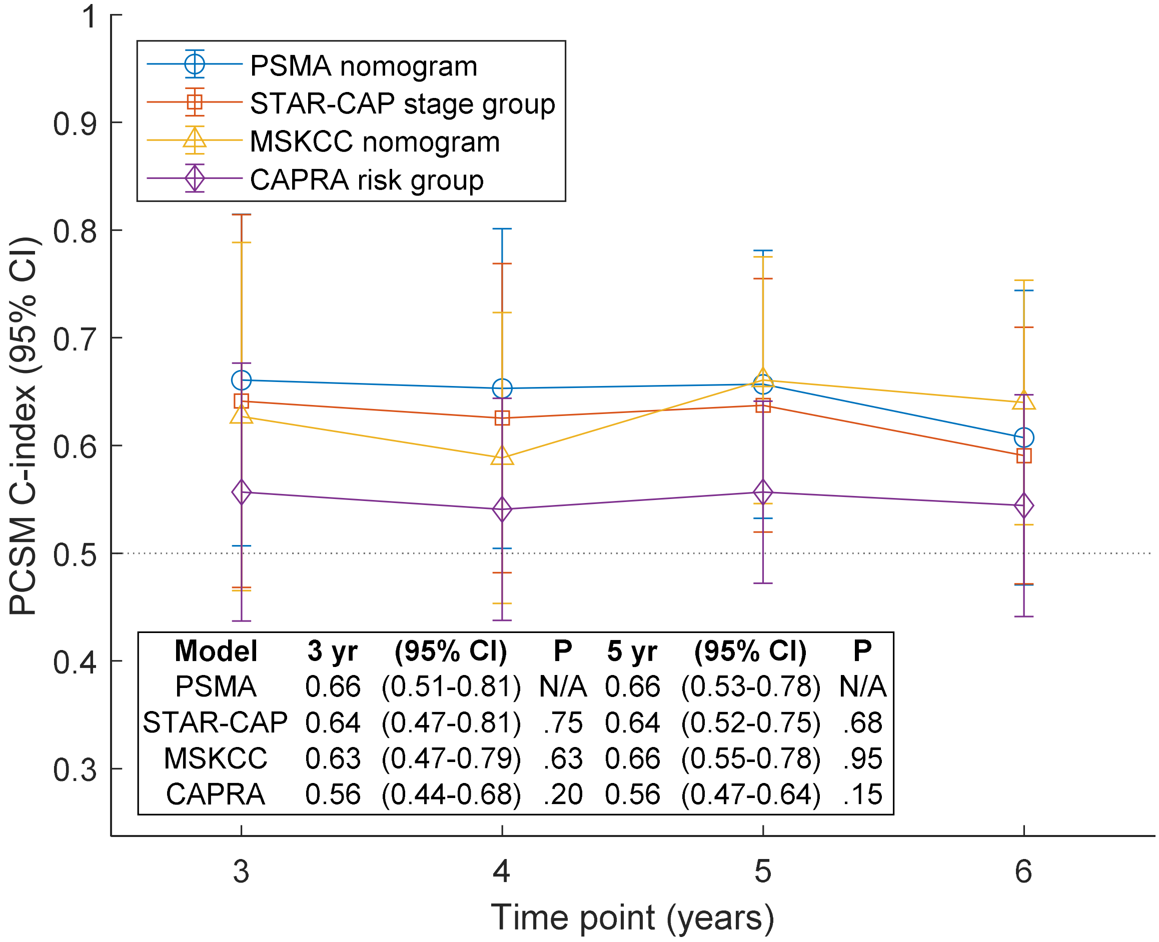

Model	3 yr	(95% CI)	P	5 yr	(95% CI)	P
PSMA	0.66	(0.51-0.81)	N/A	0.66	(0.53-0.78)	N/A
STAR-CAP	0.64	(0.47-0.81)	.75	0.64	(0.52-0.75)	.68
MSKCC	0.63	(0.47-0.79)	.63	0.66	(0.55-0.78)	.95
CAPRA	0.56	(0.44-0.68)	.20	0.56	(0.47-0.64)	.15

© 2021 Xiang M et al. *JAMA Network Open.*

D)

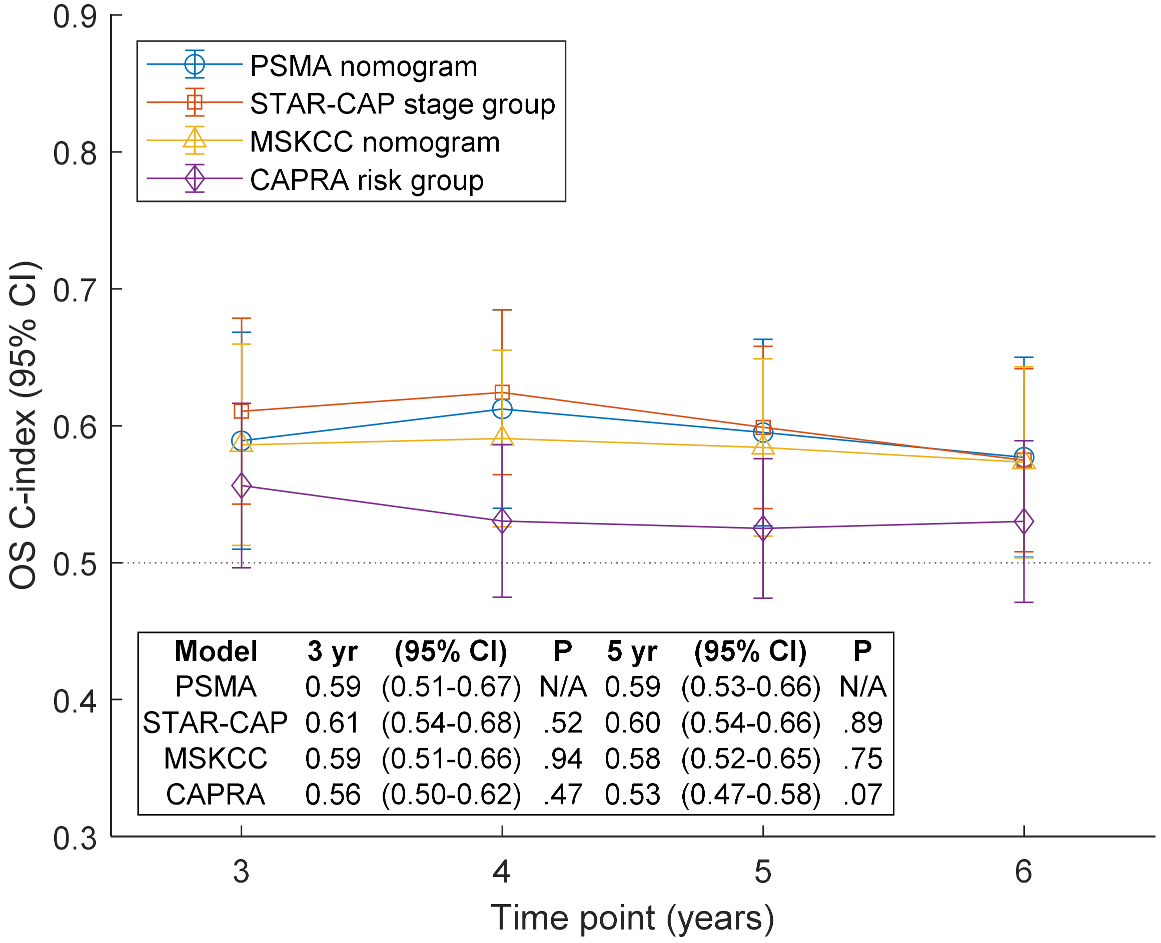

© 2021 Xiang M et al. *JAMA Network Open.*

eFigure 12. Performance of the PSMA Nomogram and Other Models in the NCDB Cohort, Further Stratified by Type of Treatment

Performance assessed by the concordance indices (C-indices) for overall survival (OS) in patients treated with A) radical prostatectomy, B) external beam radiation, or C) external beam radiation plus brachytherapy. P-values are for comparisons versus the PSMA nomogram at the 3- and 5-year time points.

A)

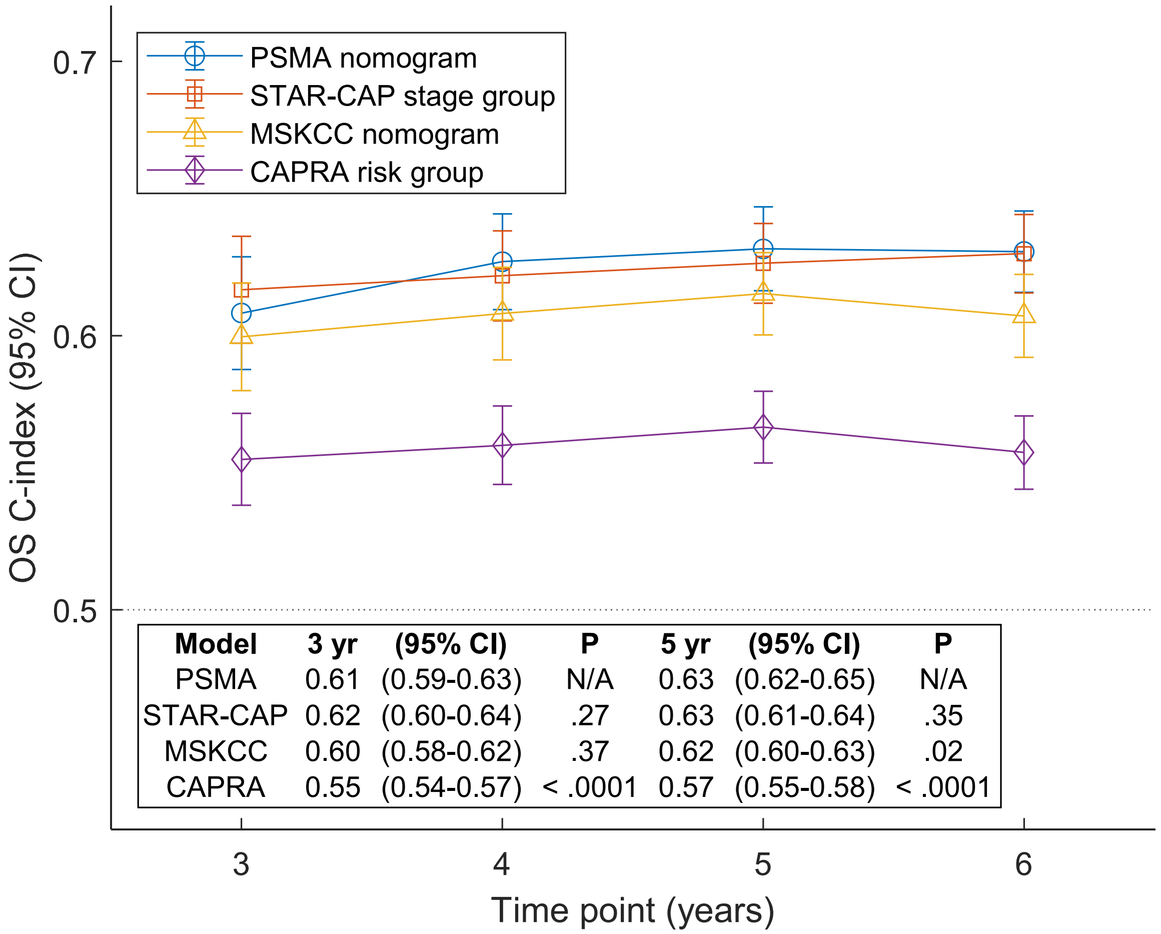

Model	3 yr	(95% CI)	P	5 yr	(95% CI)	P
PSMA	0.61	(0.59-0.63)	N/A	0.63	(0.62-0.65)	N/A
STAR-CAP	0.62	(0.60-0.64)	.27	0.63	(0.61-0.64)	.35
MSKCC	0.60	(0.58-0.62)	.37	0.62	(0.60-0.63)	.02
CAPRA	0.55	(0.54-0.57)	< .0001	0.57	(0.55-0.58)	< .0001

© 2021 Xiang M et al. *JAMA Network Open.*

B)

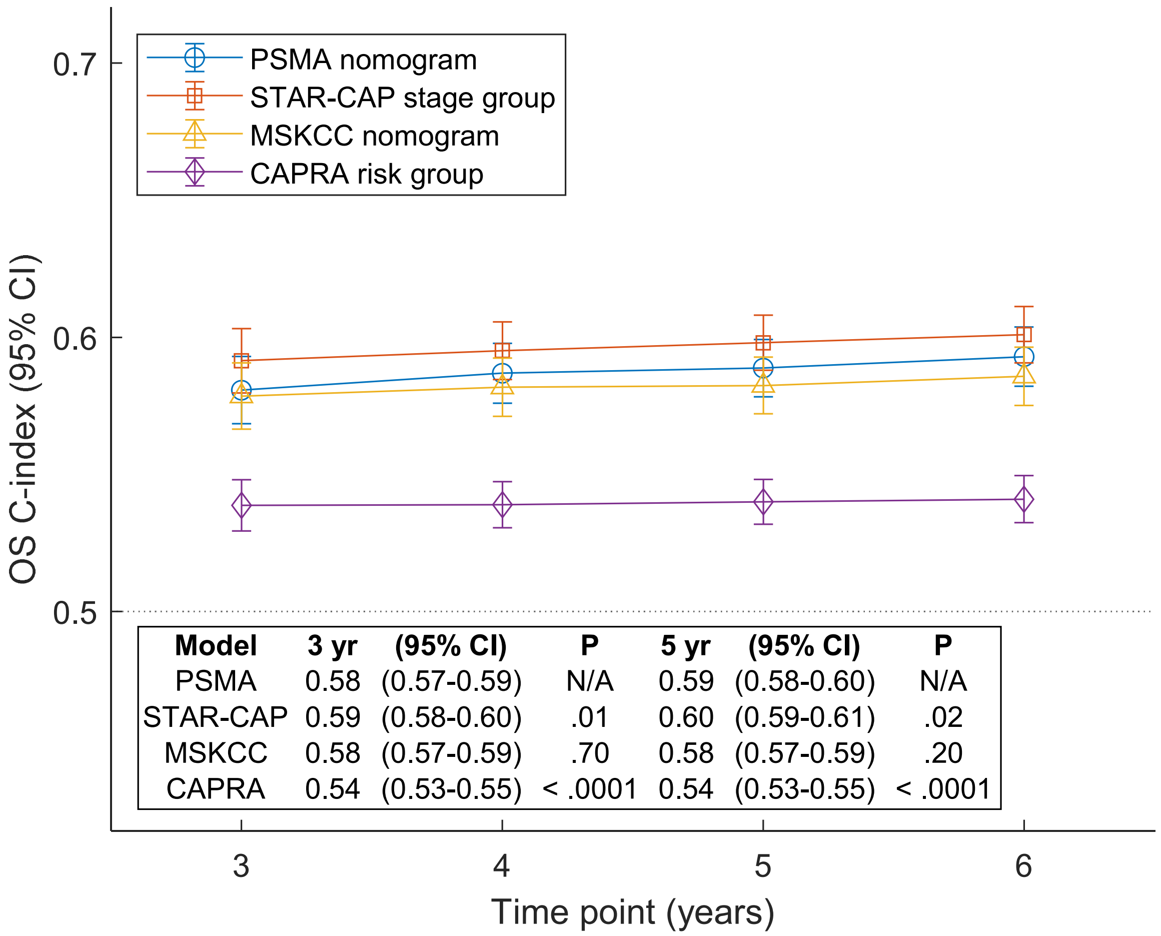

© 2021 Xiang M et al. *JAMA Network Open.*

C)

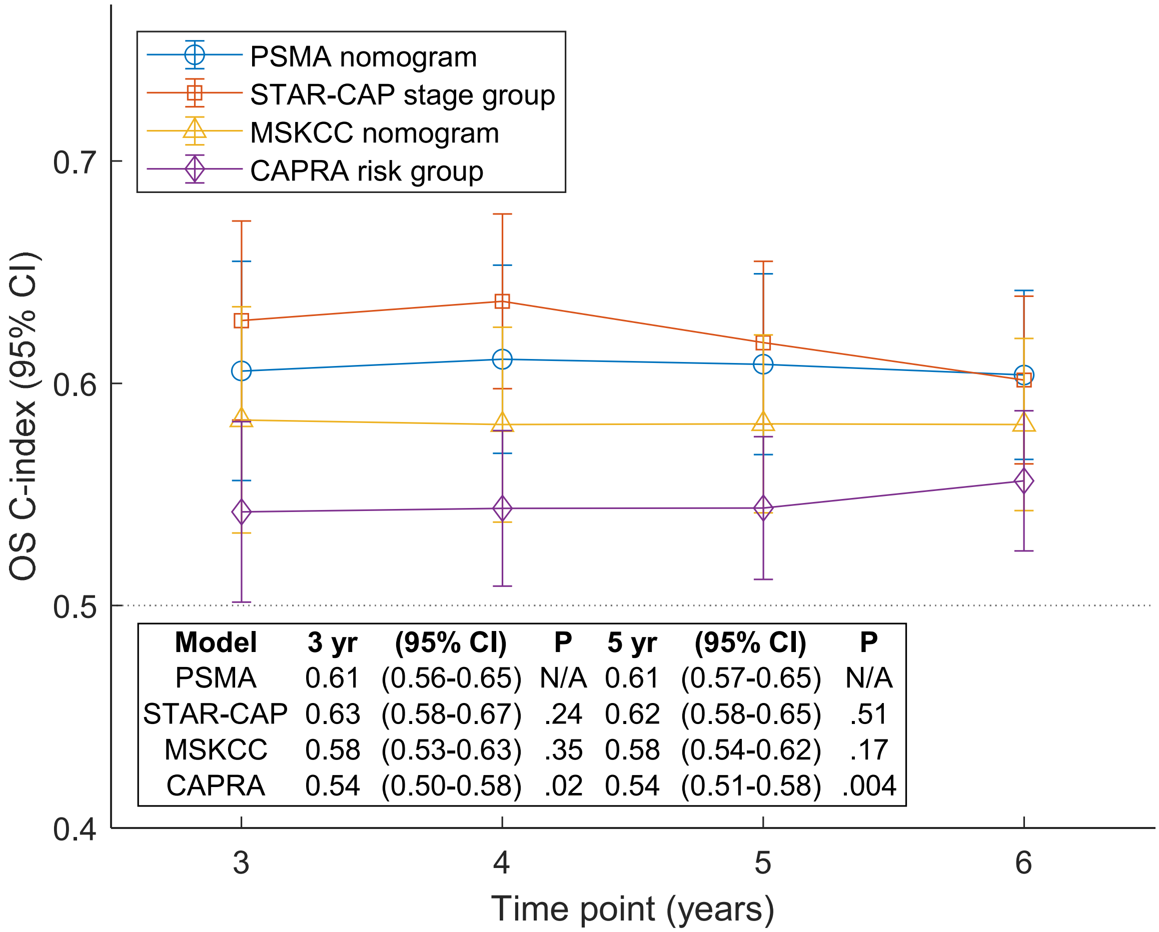

© 2021 Xiang M et al. *JAMA Network Open.*

eFigure 13. Calibration Plots for the PSMA Nomogram and the Other Models

Calibration plots for the PSMA nomogram, STAR-CAP stage groups, CAPRA risk groups, and MSKCC nomogram in the multi-institutional cohort, for the endpoints of A) biochemical recurrence (BCR), B) distant metastasis (DM), C) prostate cancer-specific mortality (PCSM), and D) overall survival. For the models with continuous values (PSMA nomogram and MSKCC nomogram), model values were binned according to deciles for smoothing.

A)

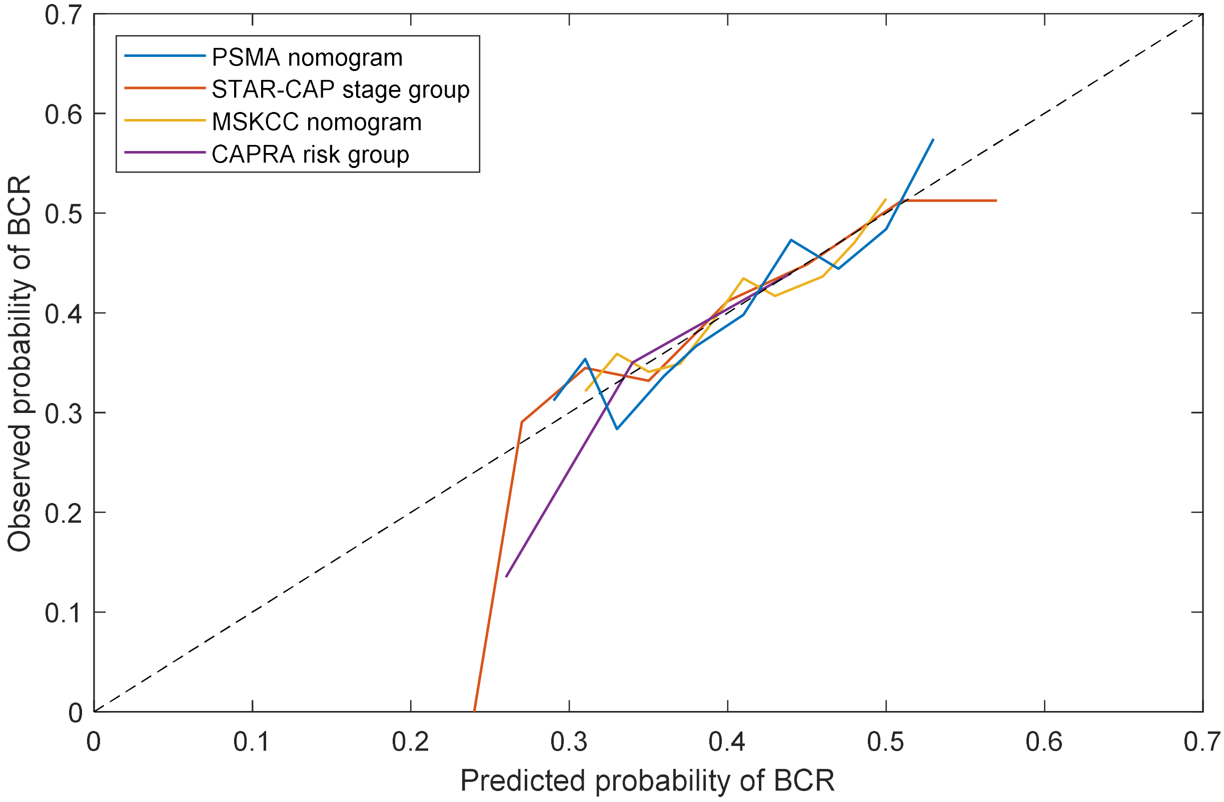

© 2021 Xiang M et al. *JAMA Network Open.*

B)

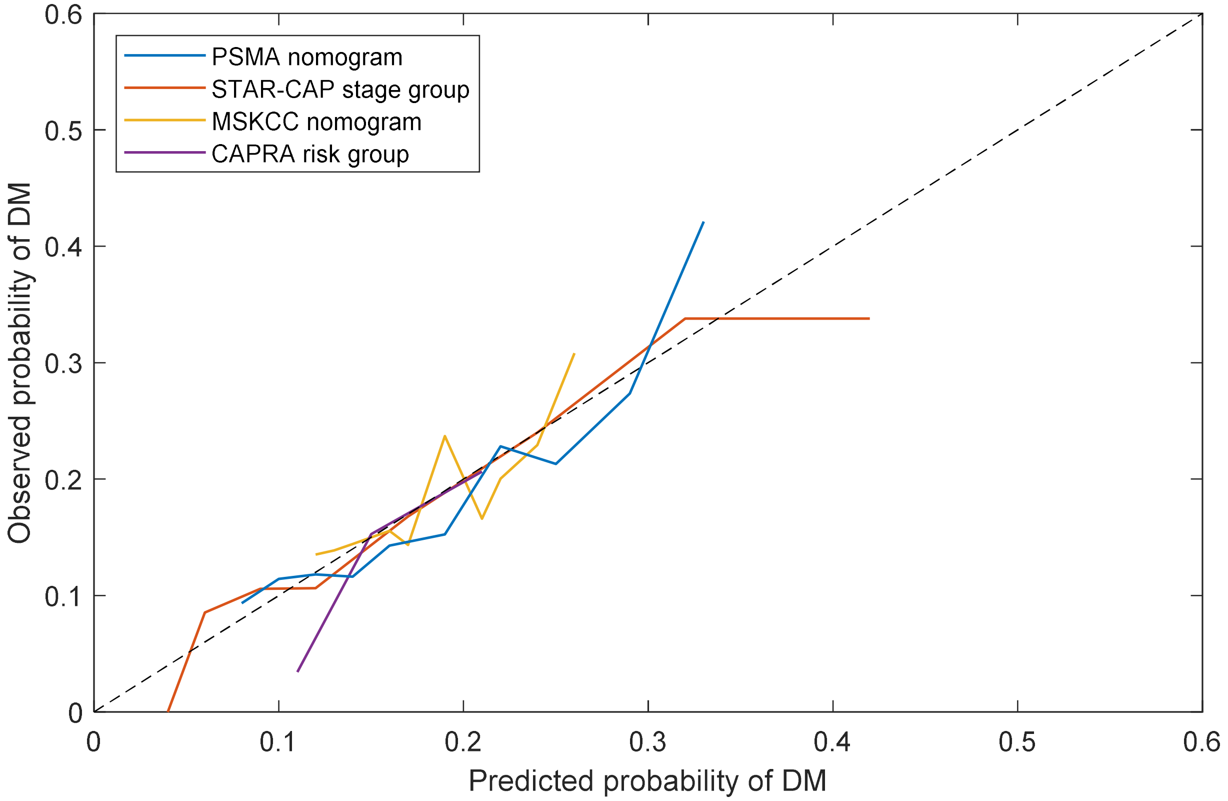

© 2021 Xiang M et al. *JAMA Network Open.*

C)

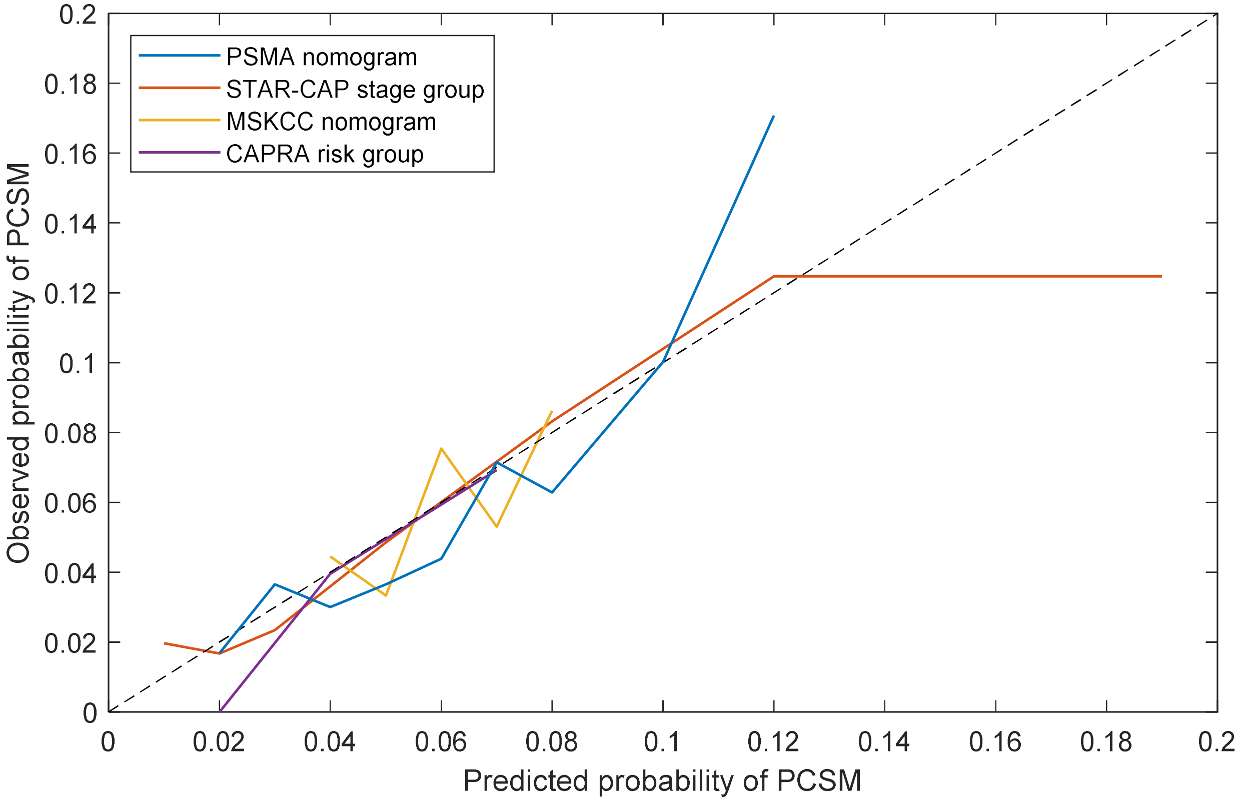

© 2021 Xiang M et al. *JAMA Network Open.*

D)

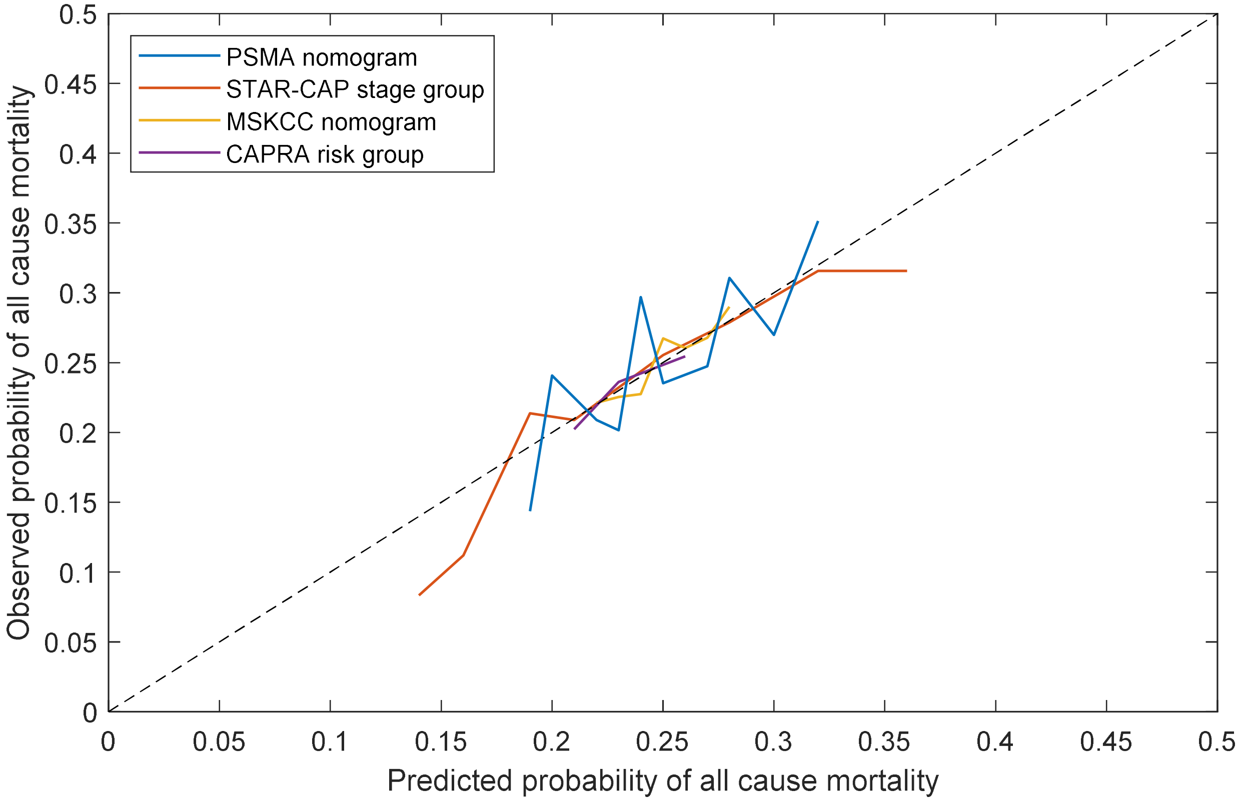

© 2021 Xiang M et al. *JAMA Network Open.*
